# Supplementary material for: Childhood Hearing Impairment in Senegal
Source: Genes (Basel). 2023 Feb 23;14(3):562. doi: 10.3390/genes14030562 (PMC10048257; doi:10.3390/genes14030562)
Supplement: Supplementary file 1 [file genes-14-00562-s001.zip › genes-2197823-supplementary.pdf]

Table S1. Distribution of the participants according to the settings across the 13 administrative regions

	Settings			
	Schools for the deaf	Children hospital	Community	Total
Regions				
DAKAR	115	33	35	183
THIES	74	0	12	86
DIOURBEL	12	0	22	34
KAOLACK	34	0	7	41
MATAM	5	0	5	10
SAINT-LOUIS	1	0	4	5
KAFFRINE	8	0	1	9
FATICK	3	0	5	8
TAMBA COUNDA	0	0	3	3
KOLDA	0	0	7	7
ZIGUINCHOR	4	0	4	8
KEDOUGOU	0	0	2	2
LOUGA	6	0	4	10
Total	262	33	111	406

Figure S1. 103 Pedigrees of ascertained families segregating Hearing Impairment

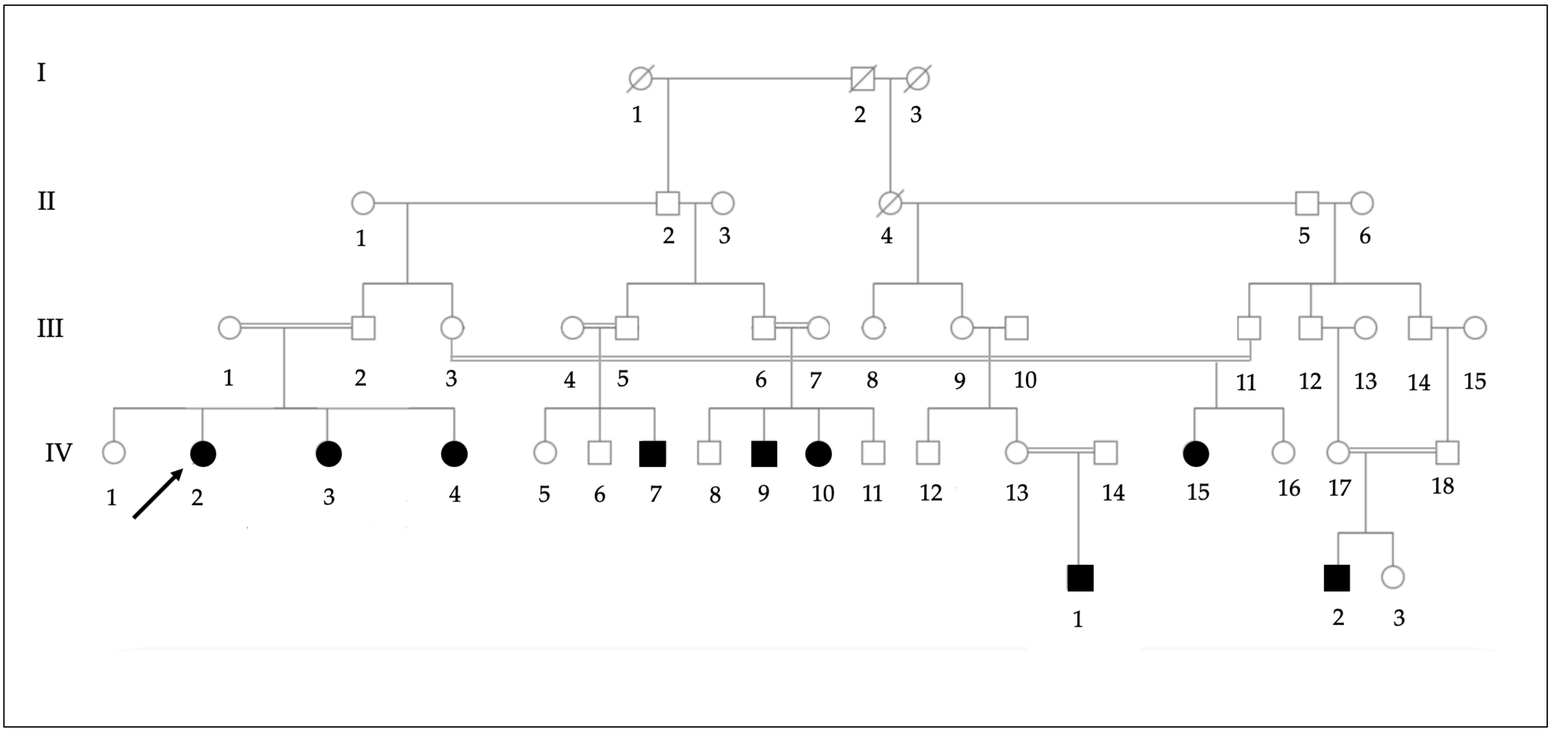

Family1. Showing nine affected individuals from consanguineous marriages segregating autosomal recessive non-syndromic hearing impairment.

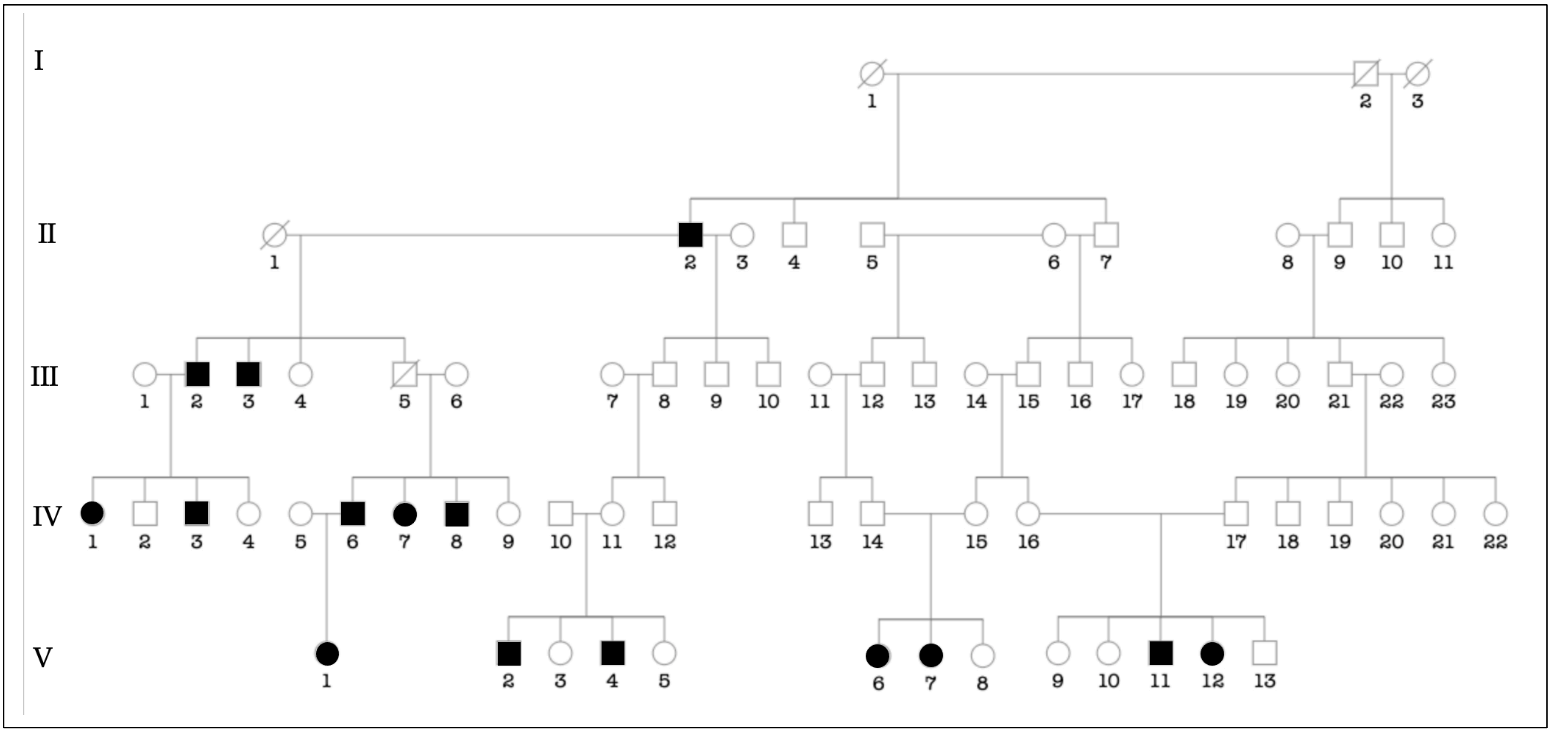

Family2. Showing fifteen affected individuals from consanguineous marriages segregating autosomal recessive non-syndromic hearing impairment.

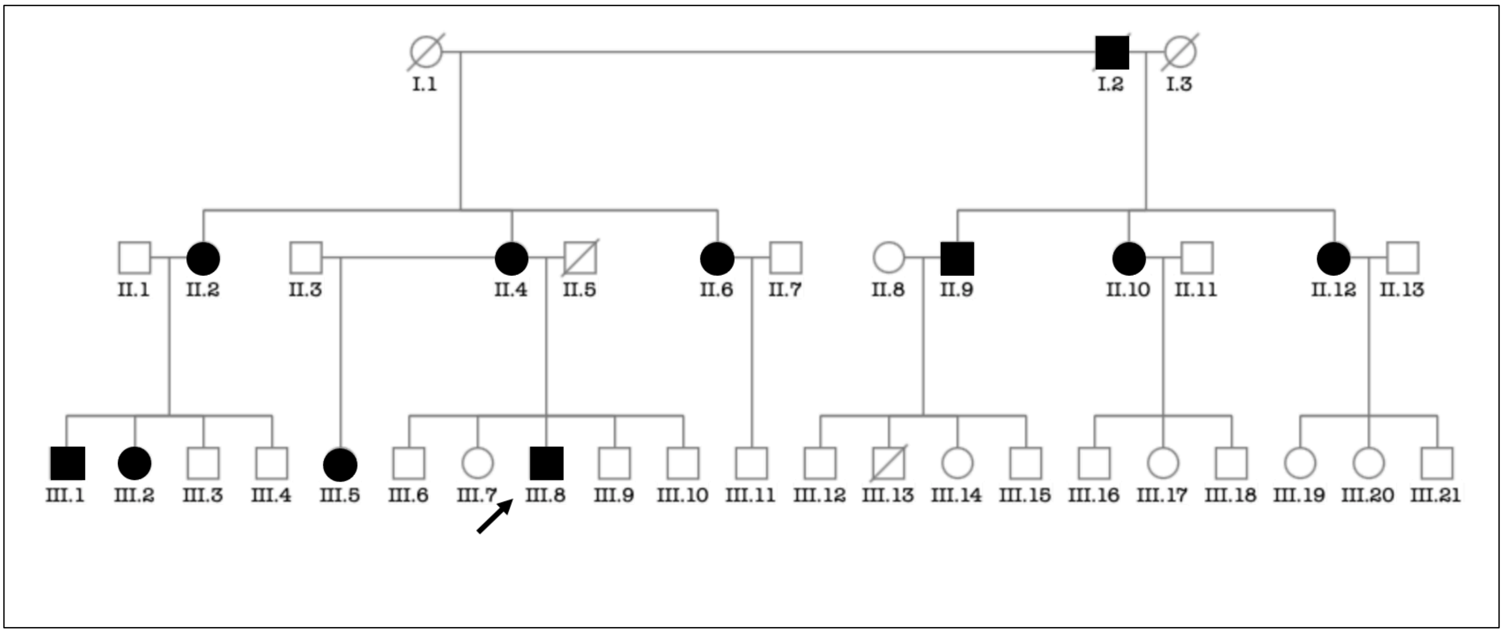

Family3. Showing eleven affected individuals segregating autosomal dominant post lingual hearing impairment.

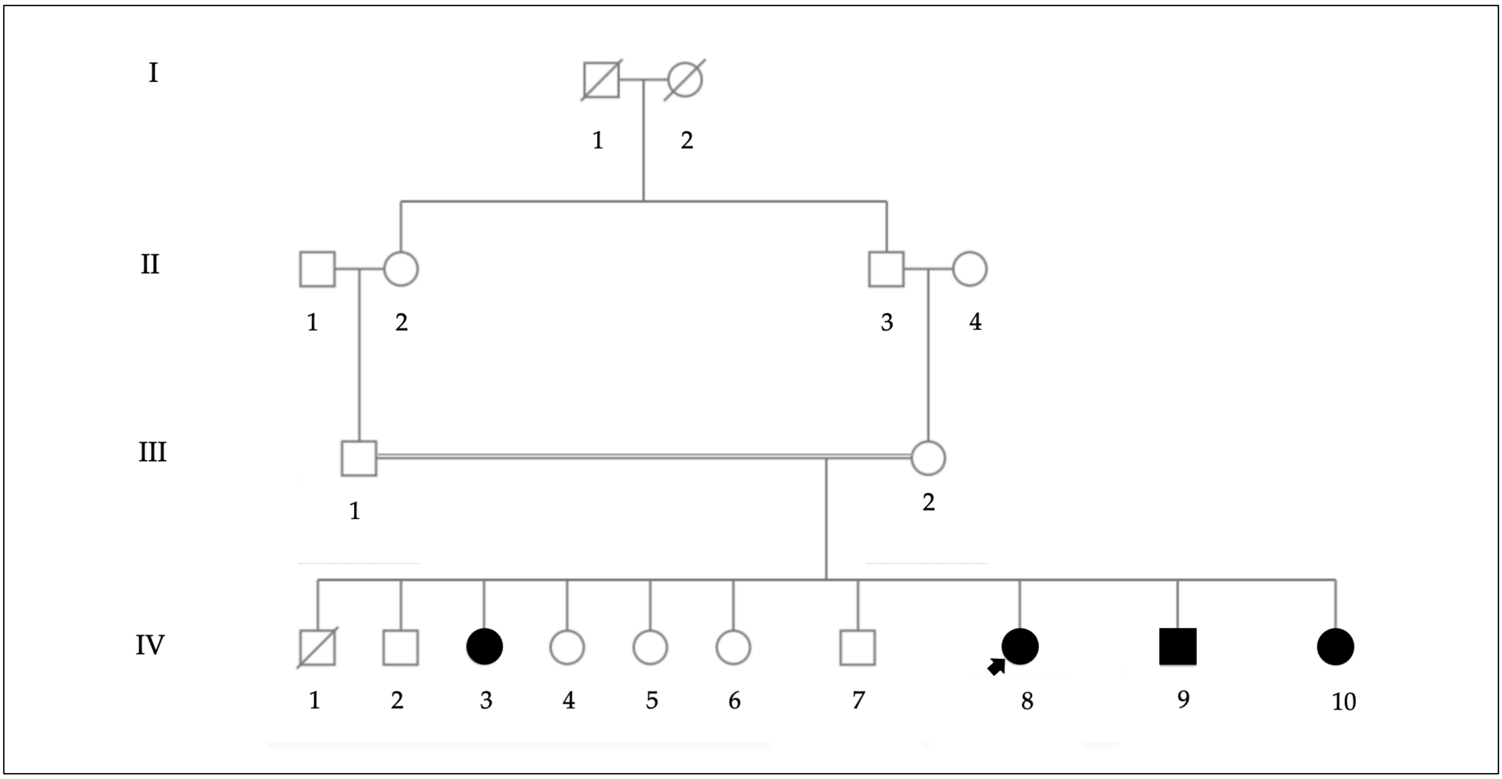

Family4. Showing four affected individuals from a consanguineous marriage segregating autosomal recessive non-syndromic hearing impairment

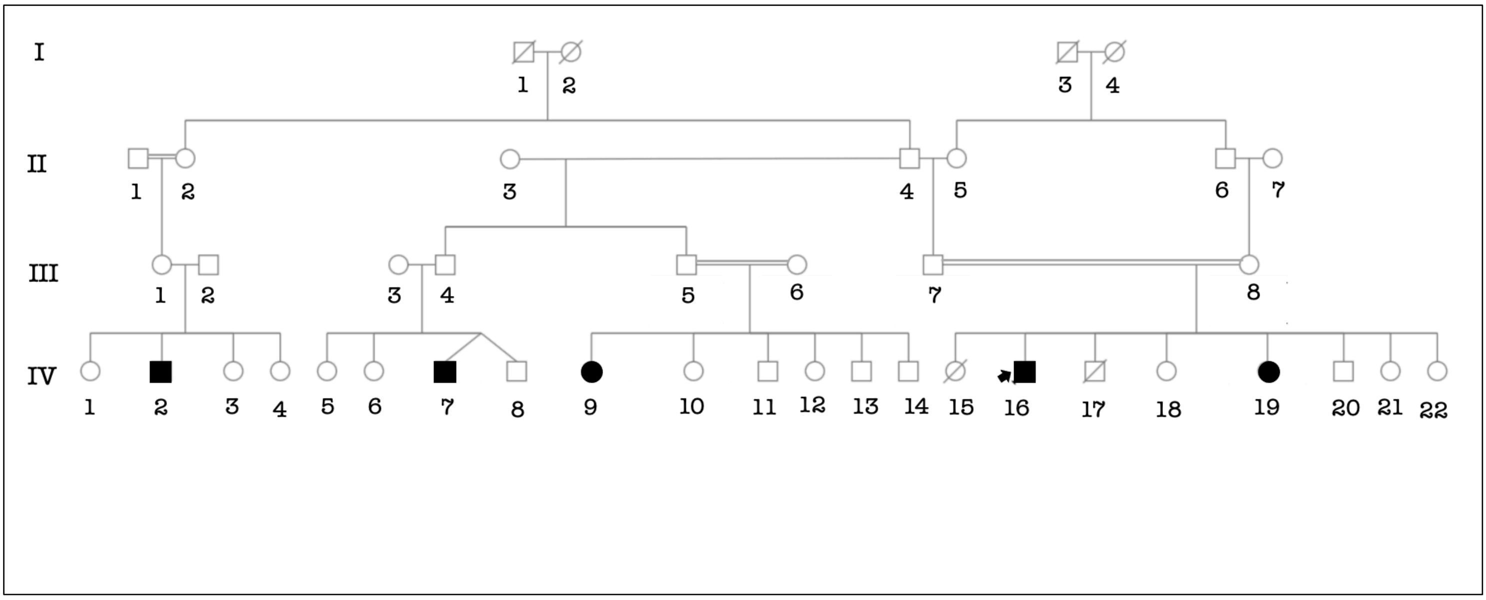

Family5. Showing five affected individuals from two consanguineous families and two affected individuals from non-consanguineous marriages.

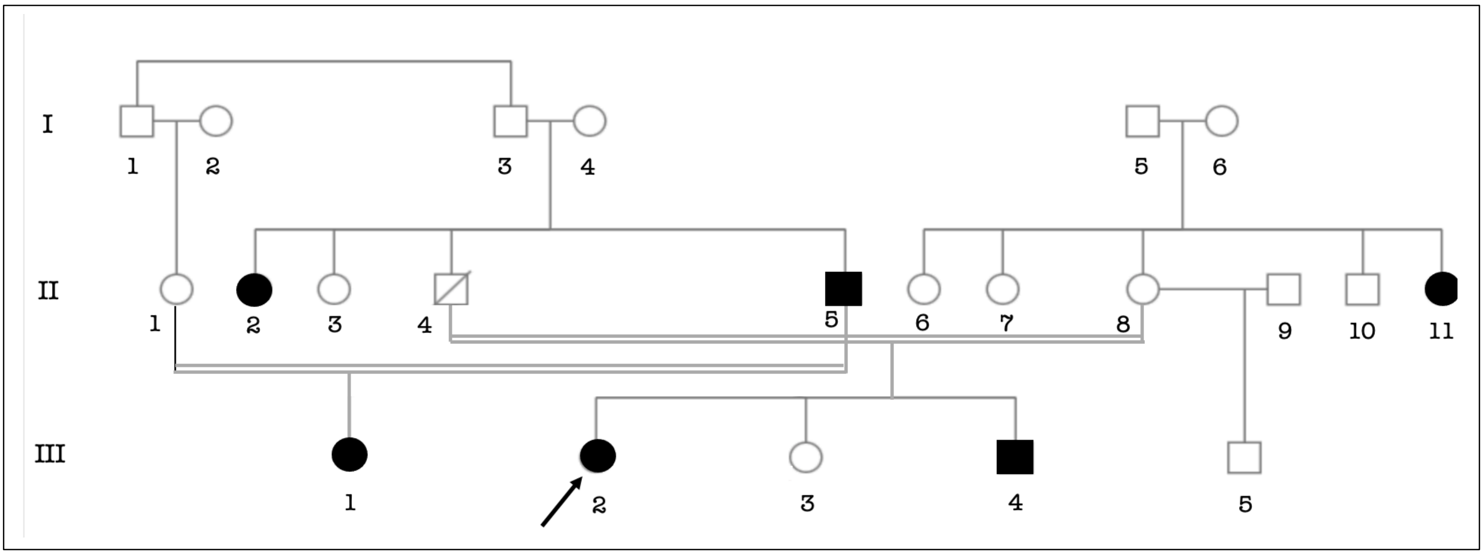

Family6. Showing six affected individuals from consanguineous marriages segregating autosomal recessive non syndromic hearing impairment.

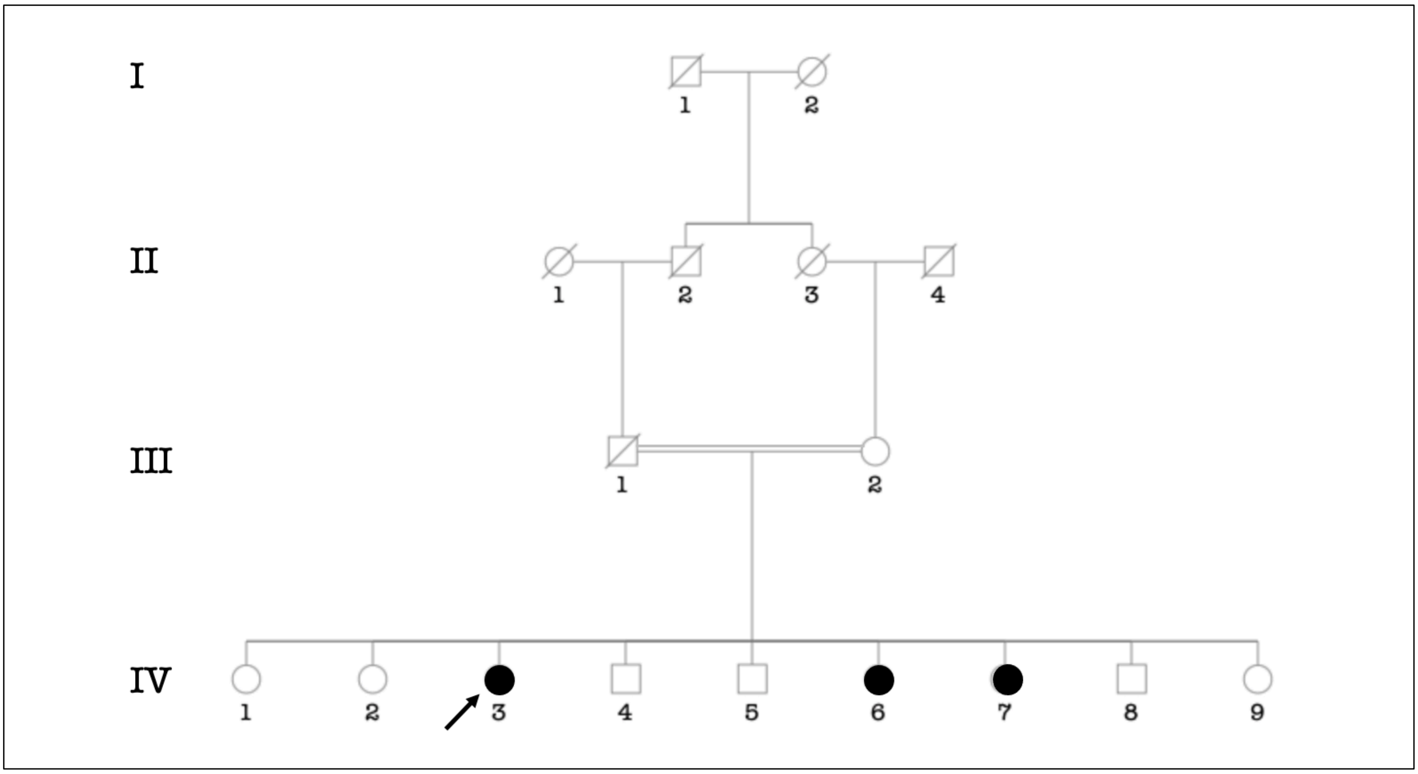

Family7. Showing three affected individuals from a consanguineous marriage segregating autosomal recessive non-syndromic

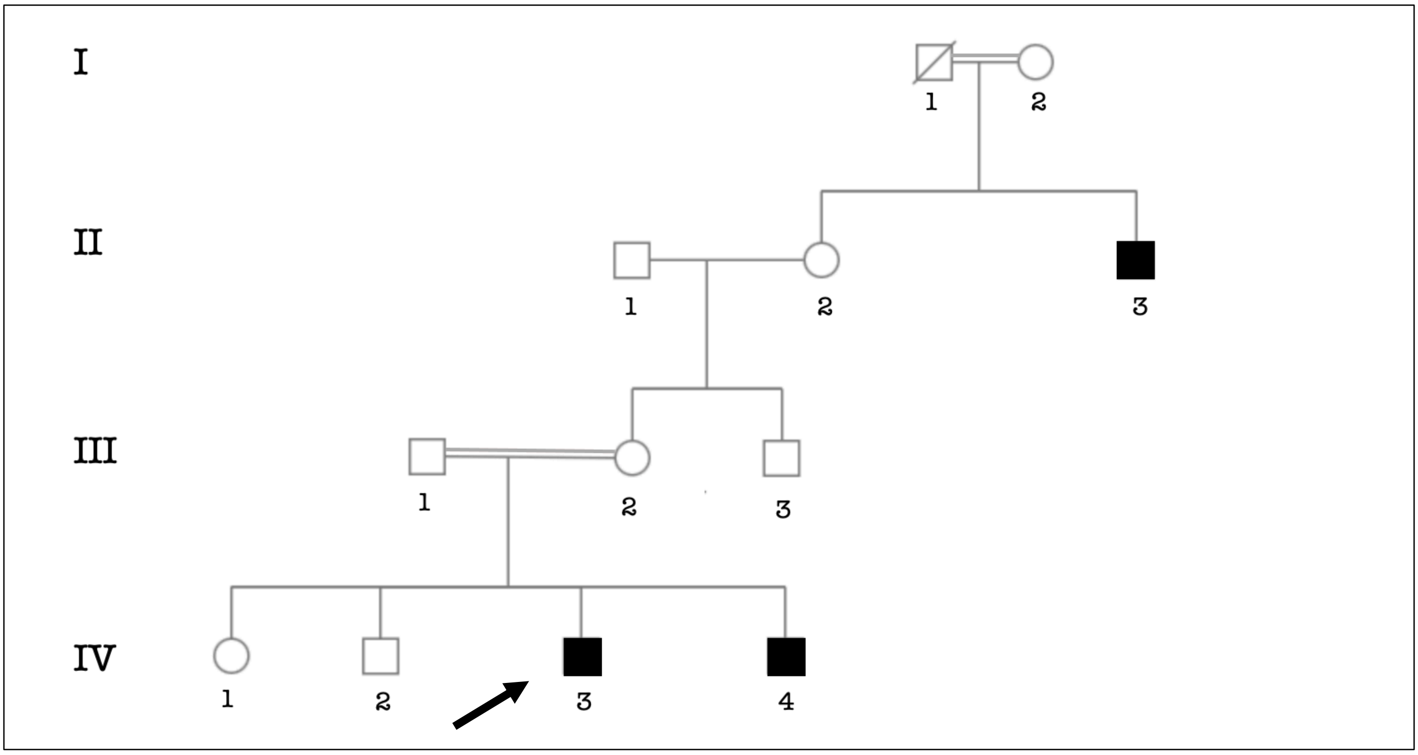

Family8. Showing three affected individuals from consanguineous marriages segregating recessive X-linked non-syndromic hearing impairment.

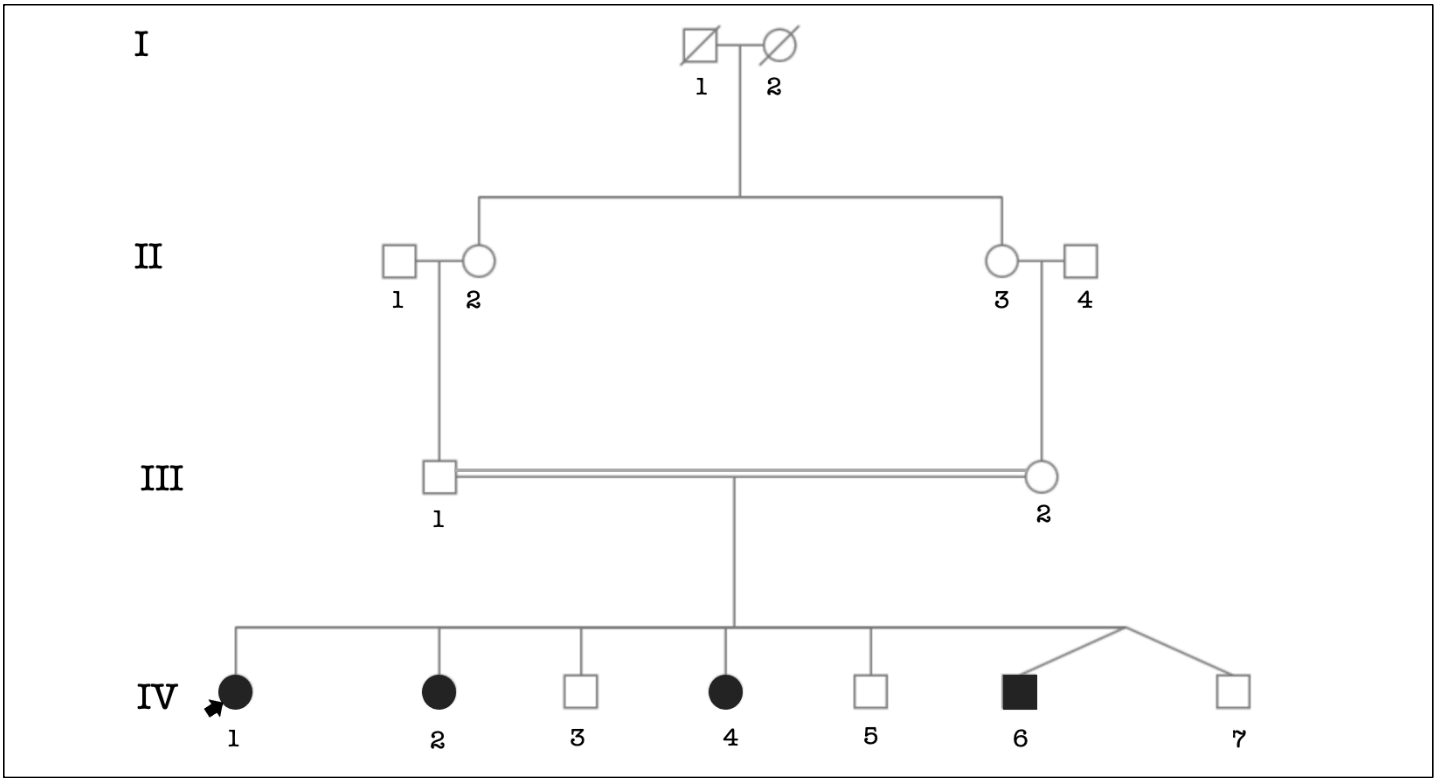

Family9. Showing four affected individuals from a consanguineous marriage segregating autosomal recessive non-syndromic hearing impairment.

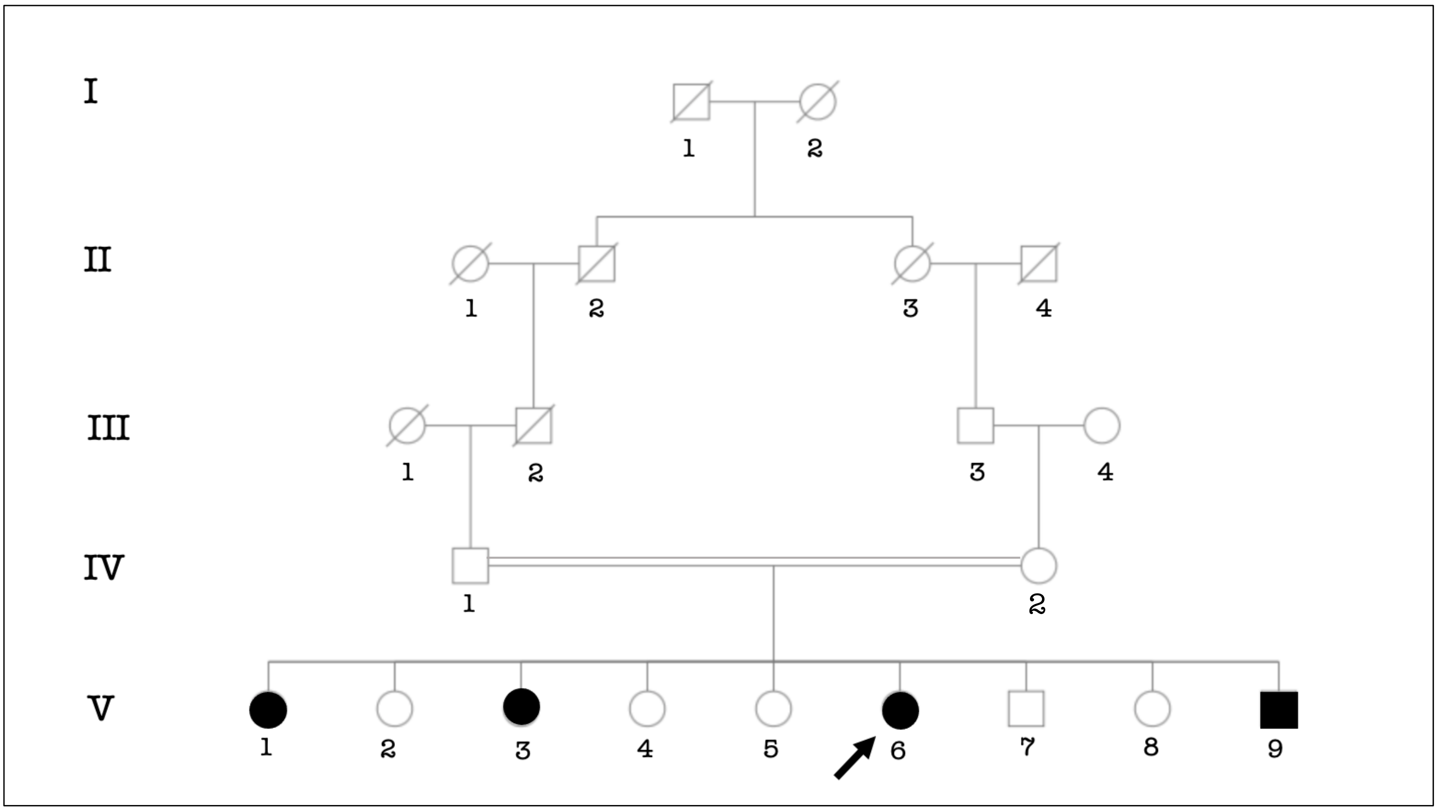

Family10. Showing four affected individuals from a consanguineous marriage segregating autosomal recessive non-syndromic hearing impairment

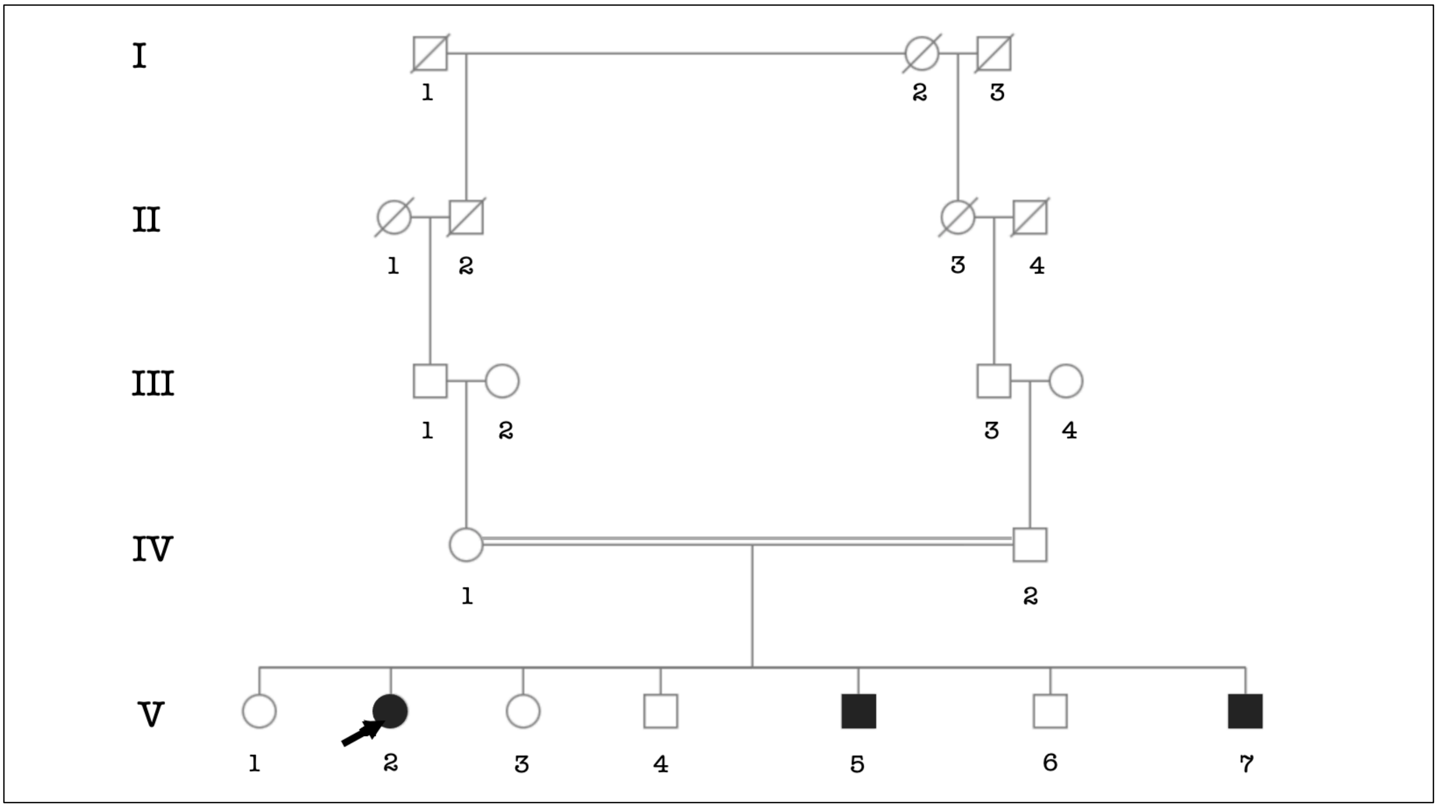

Figure11. Showing three affected individuals from a consanguineous marriage segregating autosomal recessive non-syndromic hearing impairment.

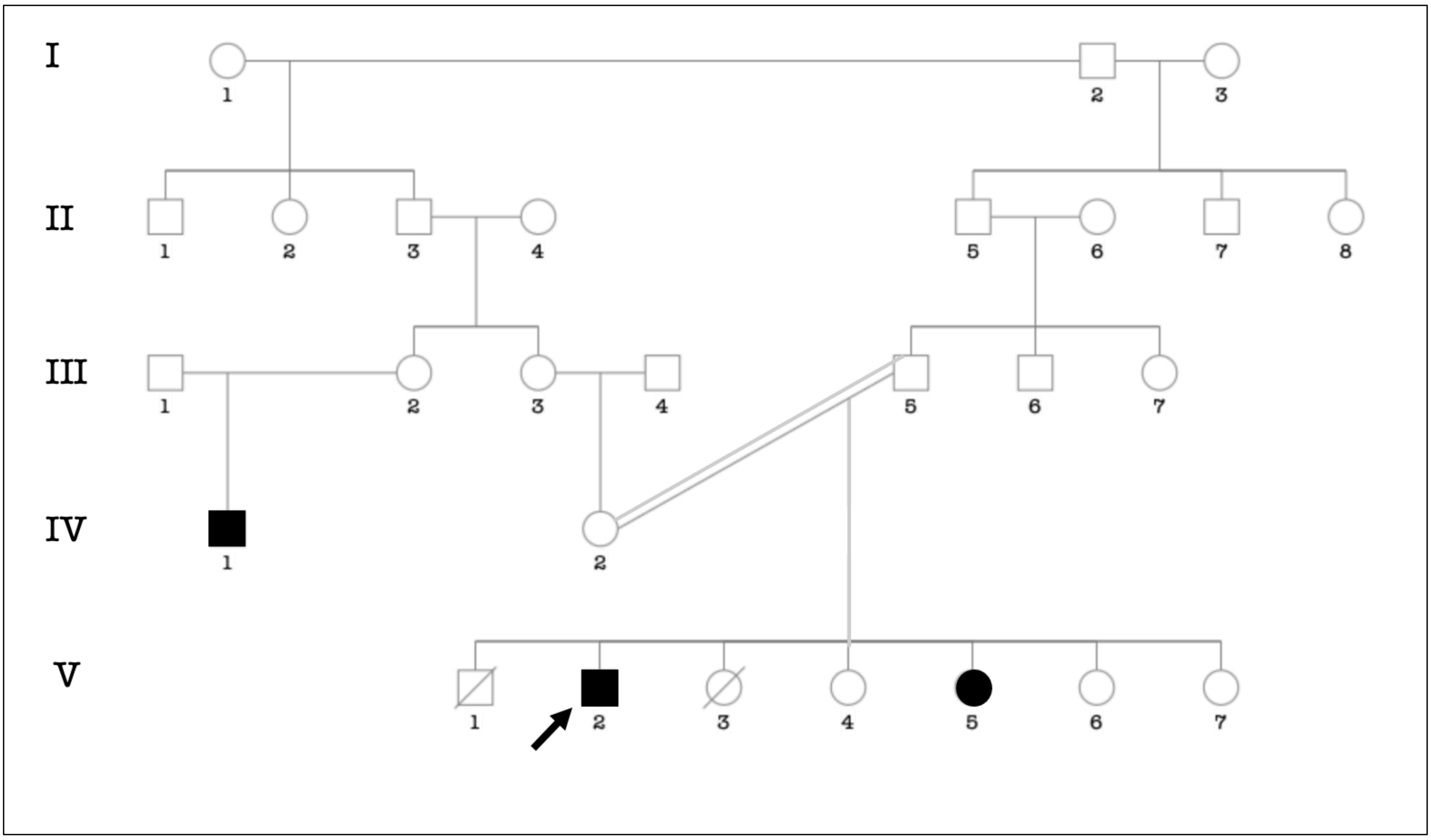

Family12. Showing three affected individuals from consanguineous marriages segregating autosomal recessive non-syndromic hearing impairment.

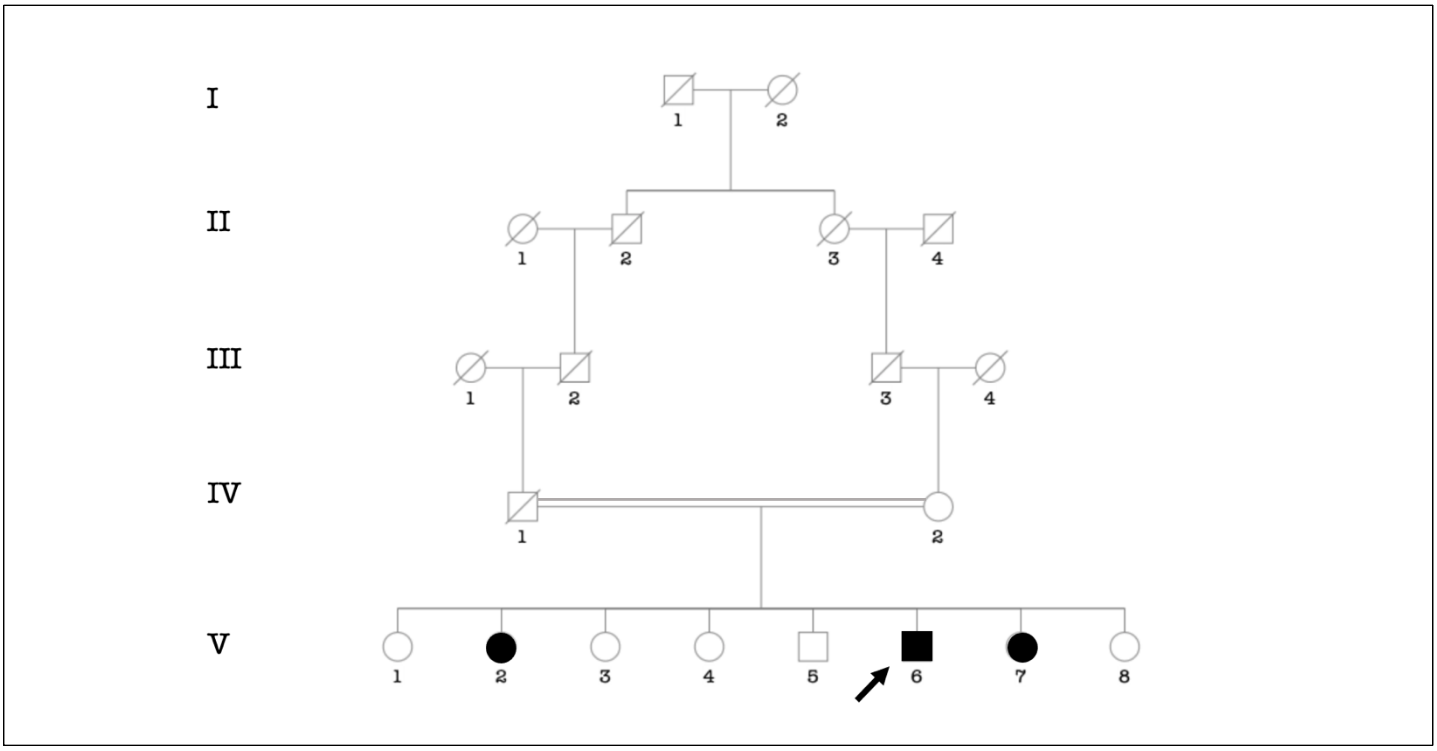

Family13. Showing three affected individuals from a consanguineous marriage segregating autosomal recessive non-syndromic hearing impairment.

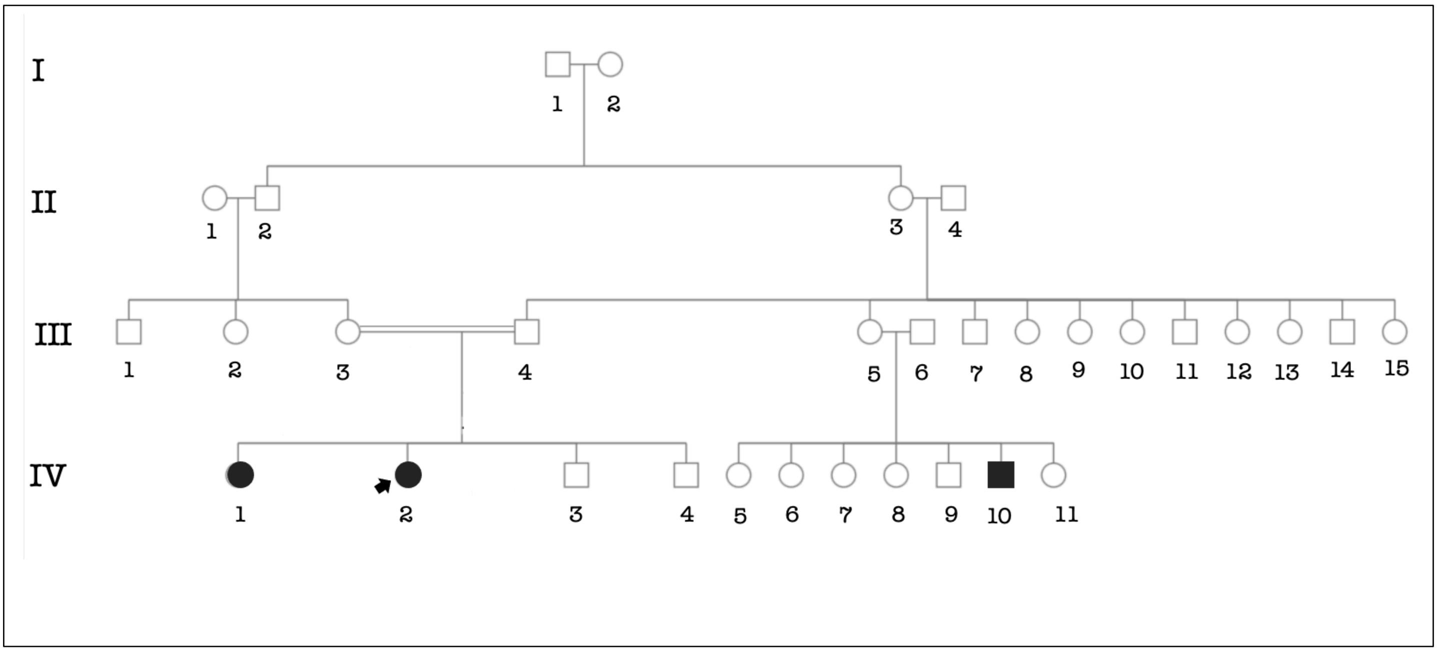

Family14. Showing three affected individuals segregating autosomal recessive non-syndromic hearing impairment.

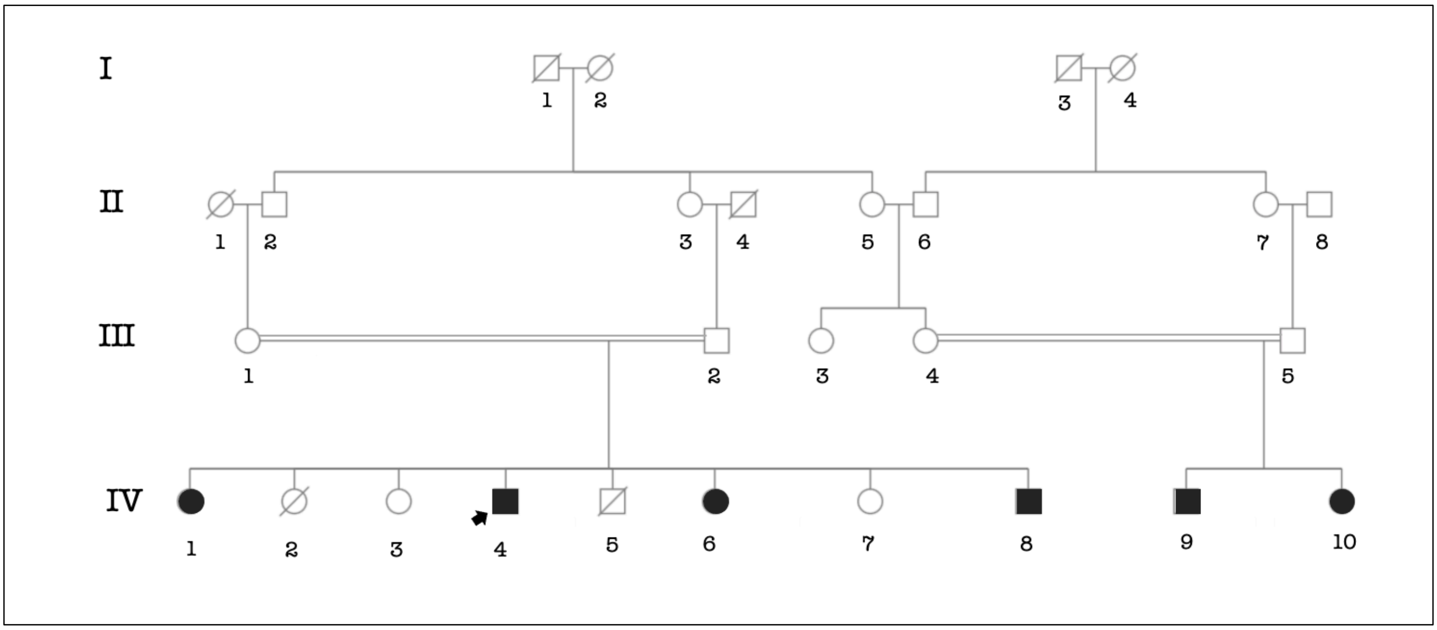

Family15. Showing six affected individuals from consanguineous marriages segregating autosomal recessive non-syndromic hearing impairment.

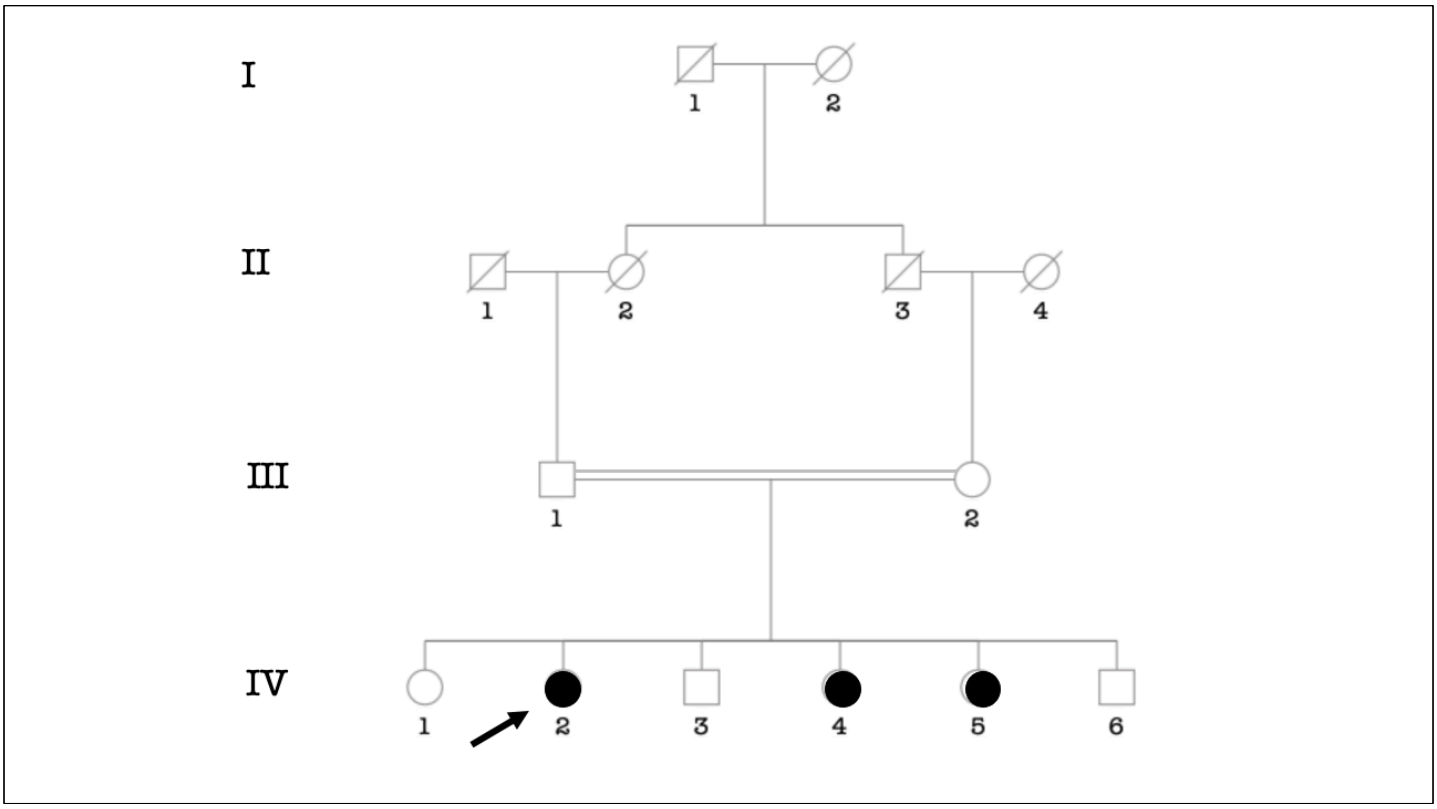

Family16. Showing three affected individuals from a consanguineous marriage segregating autosomal recessive non-syndromic hearing impairment.

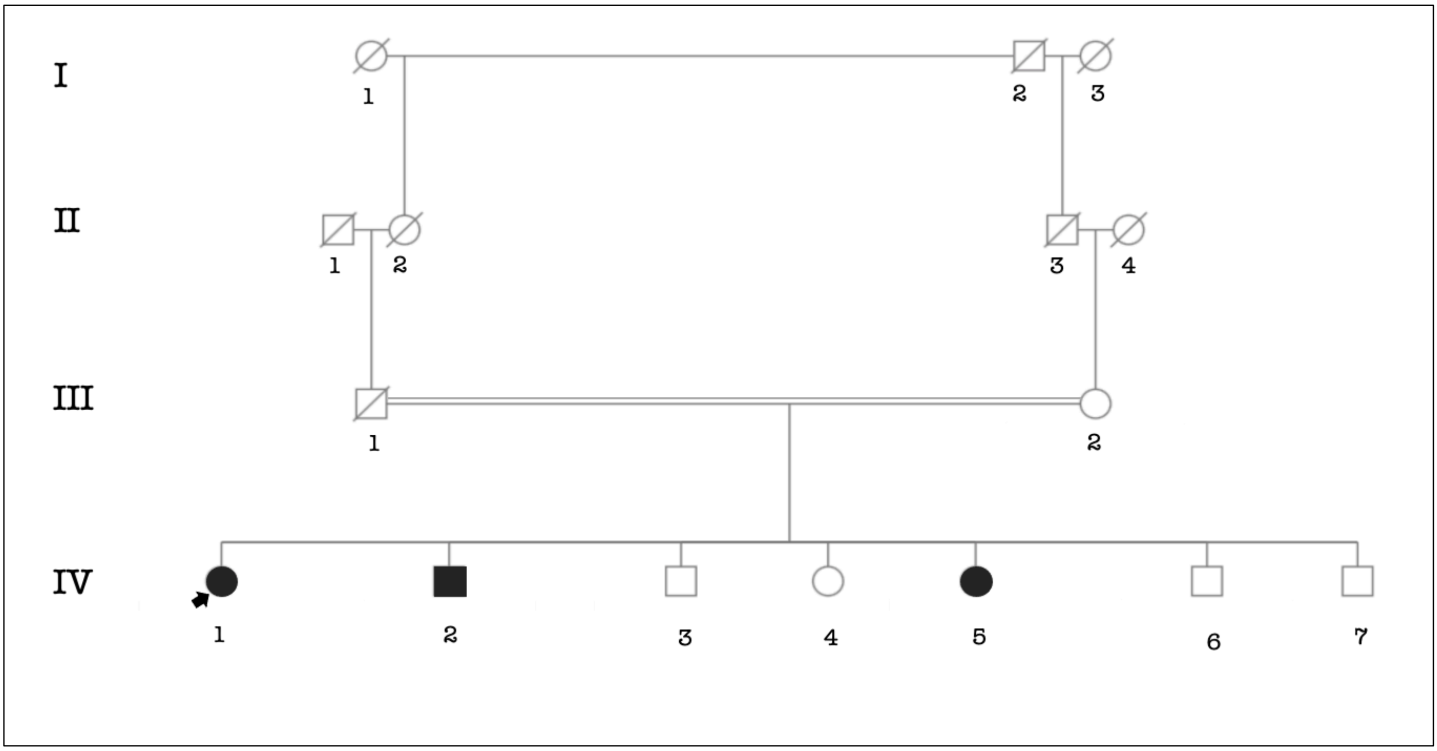

Family17. Showing three affected individuals from a consanguineous marriage segregating autosomal recessive non-syndromic hearing impairment.

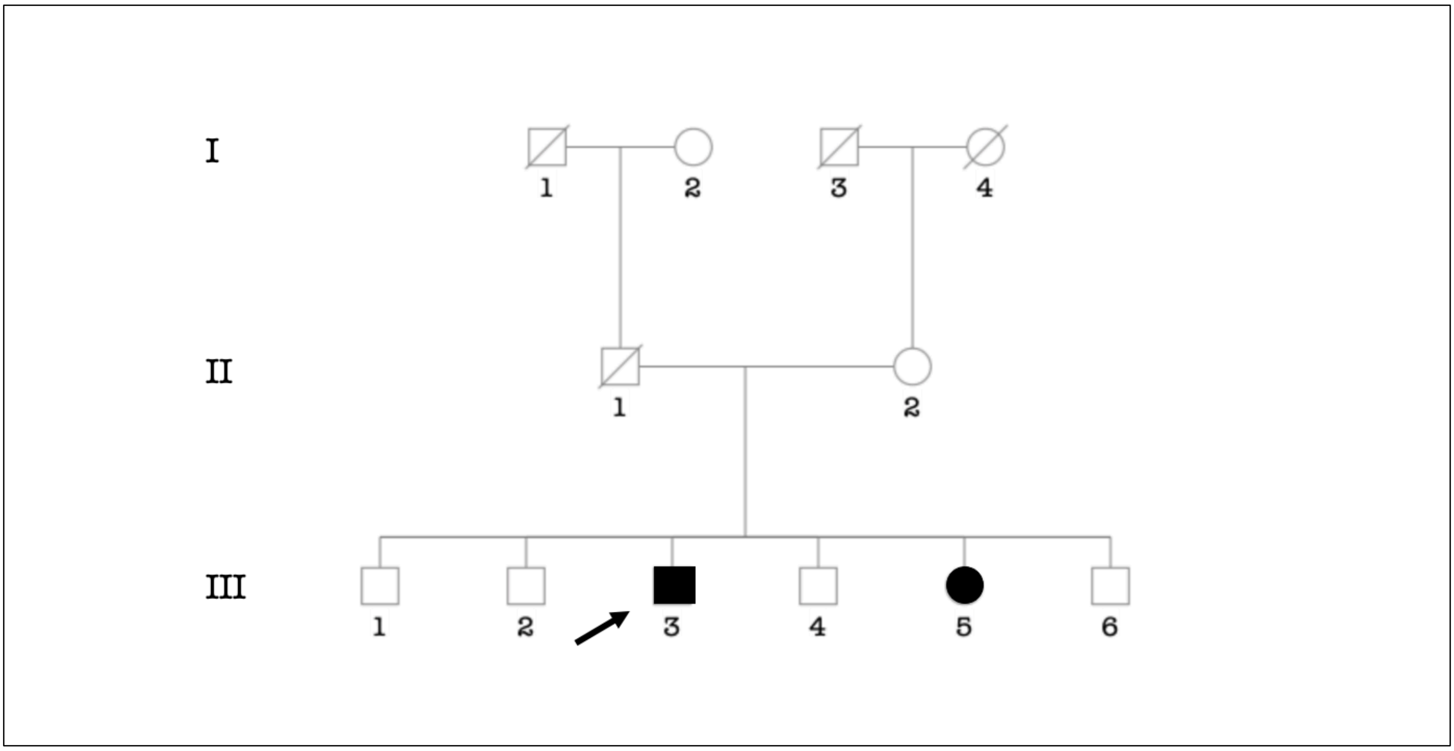

Family18. Showing two affected individuals from a non-consanguineous marriage segregating autosomal recessive non-syndromic hearing impairment.

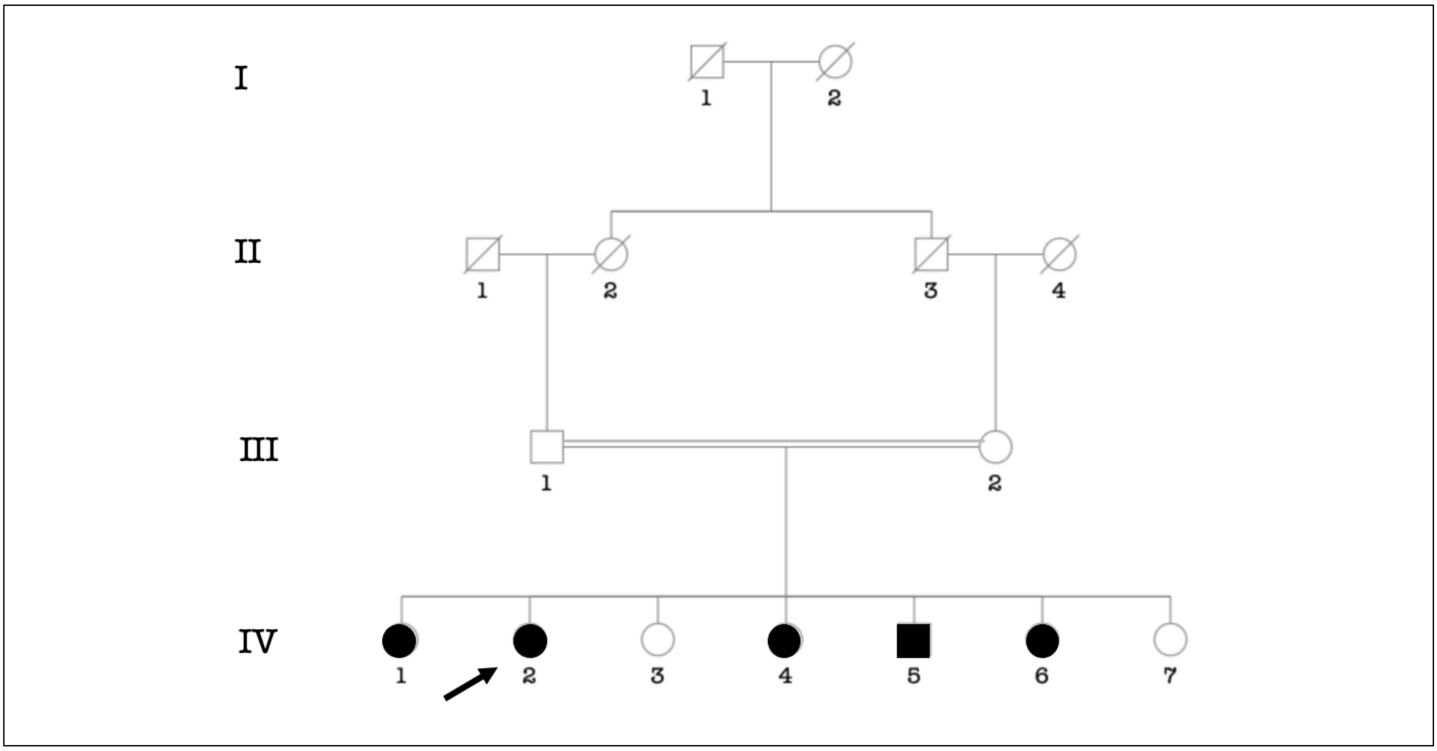

Family19. Showing five affected individuals from a consanguineous marriage segregating autosomal recessive non-syndromic hearing impairment.

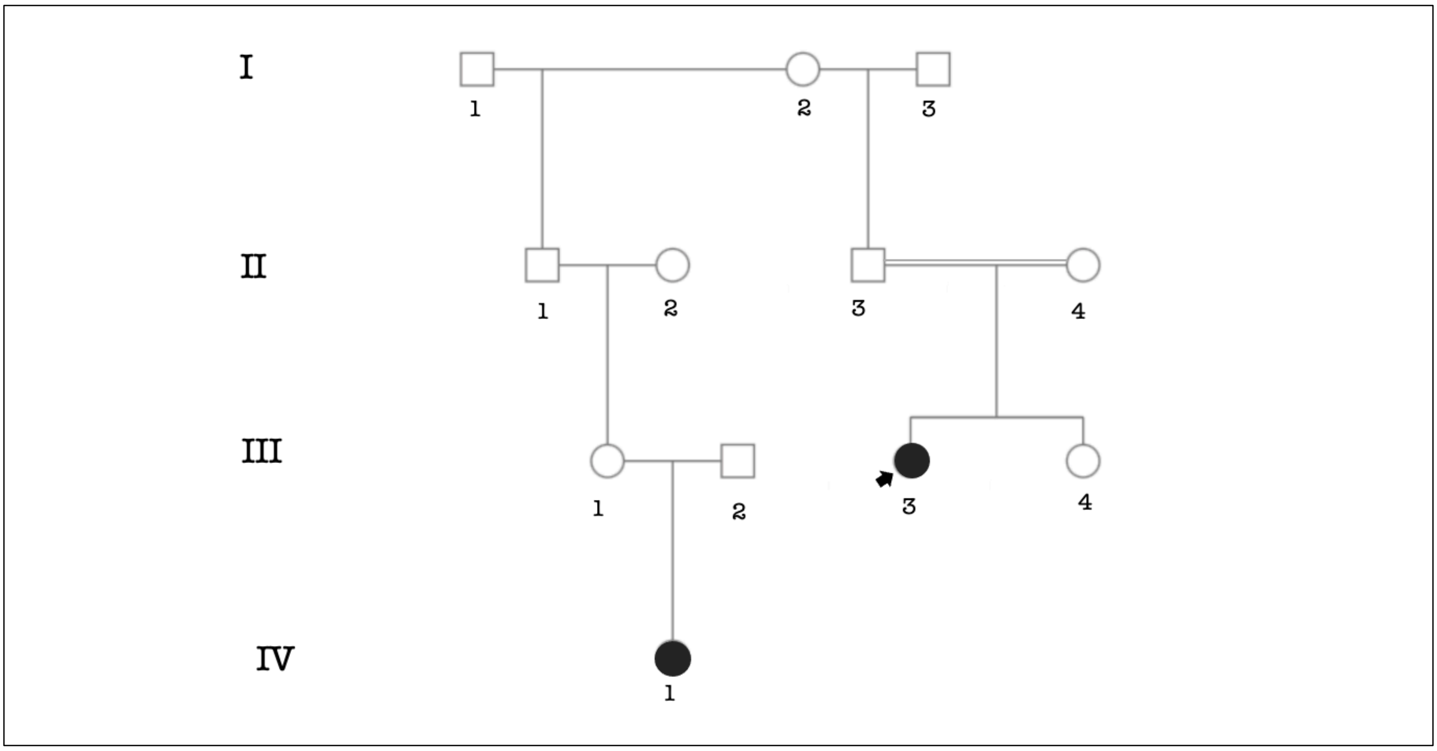

Family20. Showing two affected individuals segregating autosomal recessive non-syndromic hearing impairment.

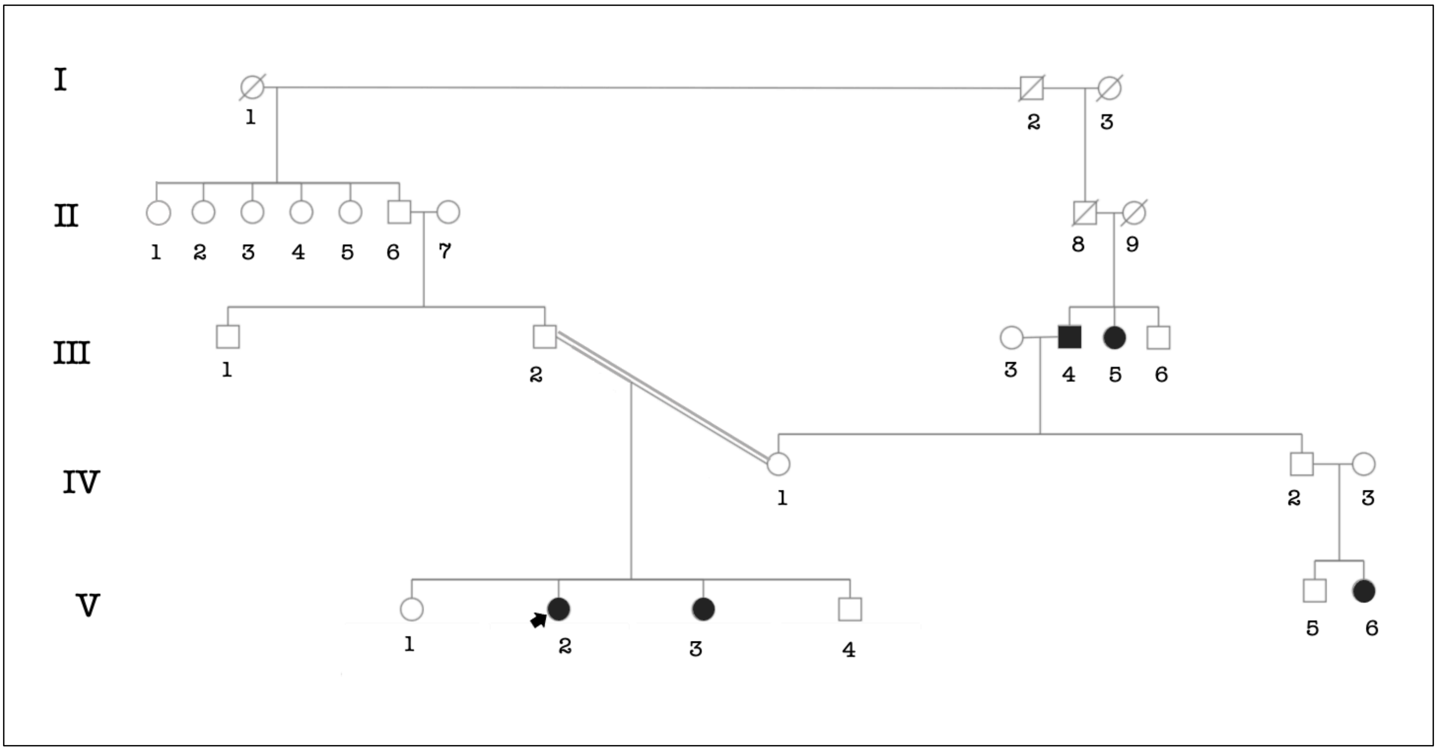

Family21. Showing five affected individuals segregating autosomal recessive non-syndromic hearing impairment.

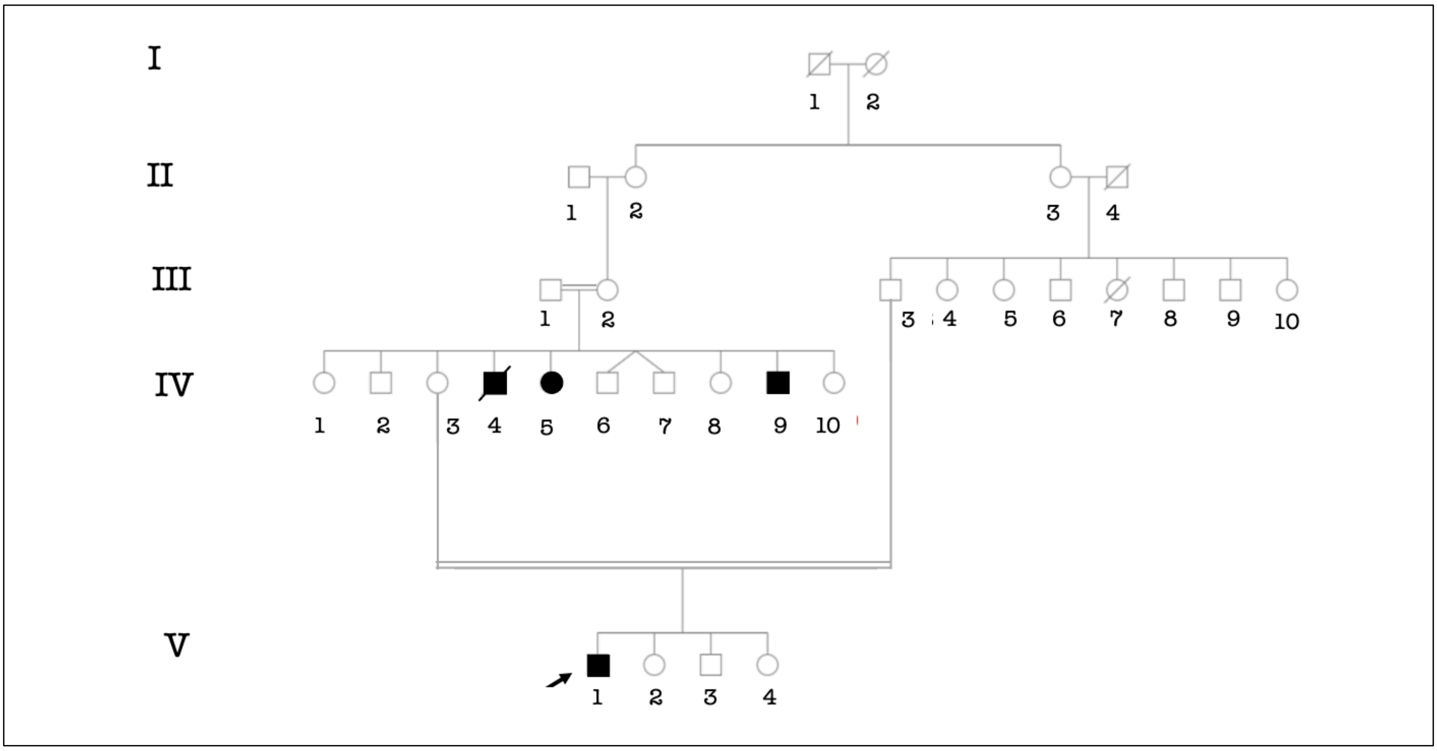

Family22. Showing four affected individuals from consanguineous marriages segregating autosomal recessive non-syndromic hearing impairment.

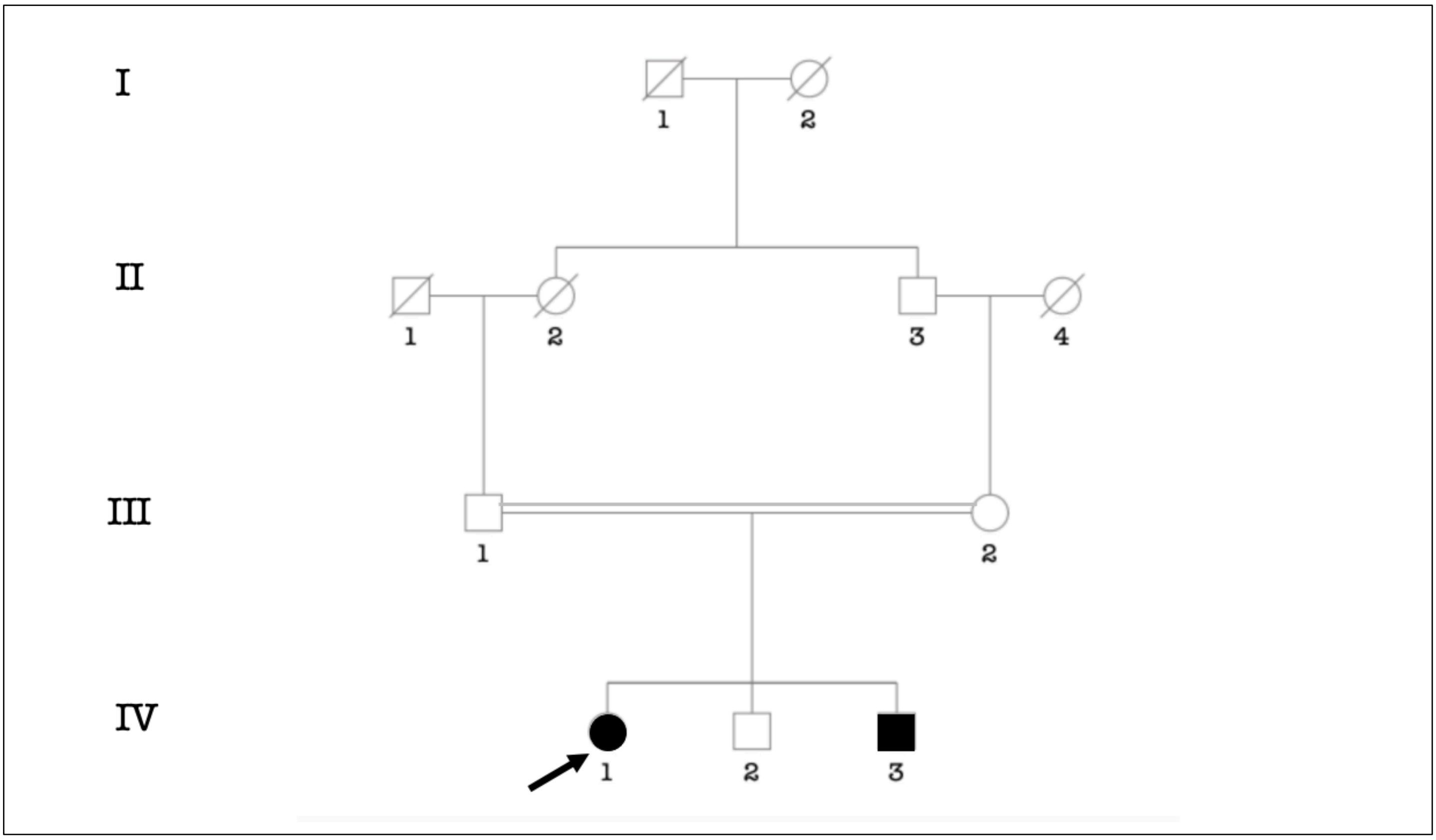

Family23. Showing two affected individuals from a consanguineous marriage segregating autosomal recessive non-syndromic hearing impairment

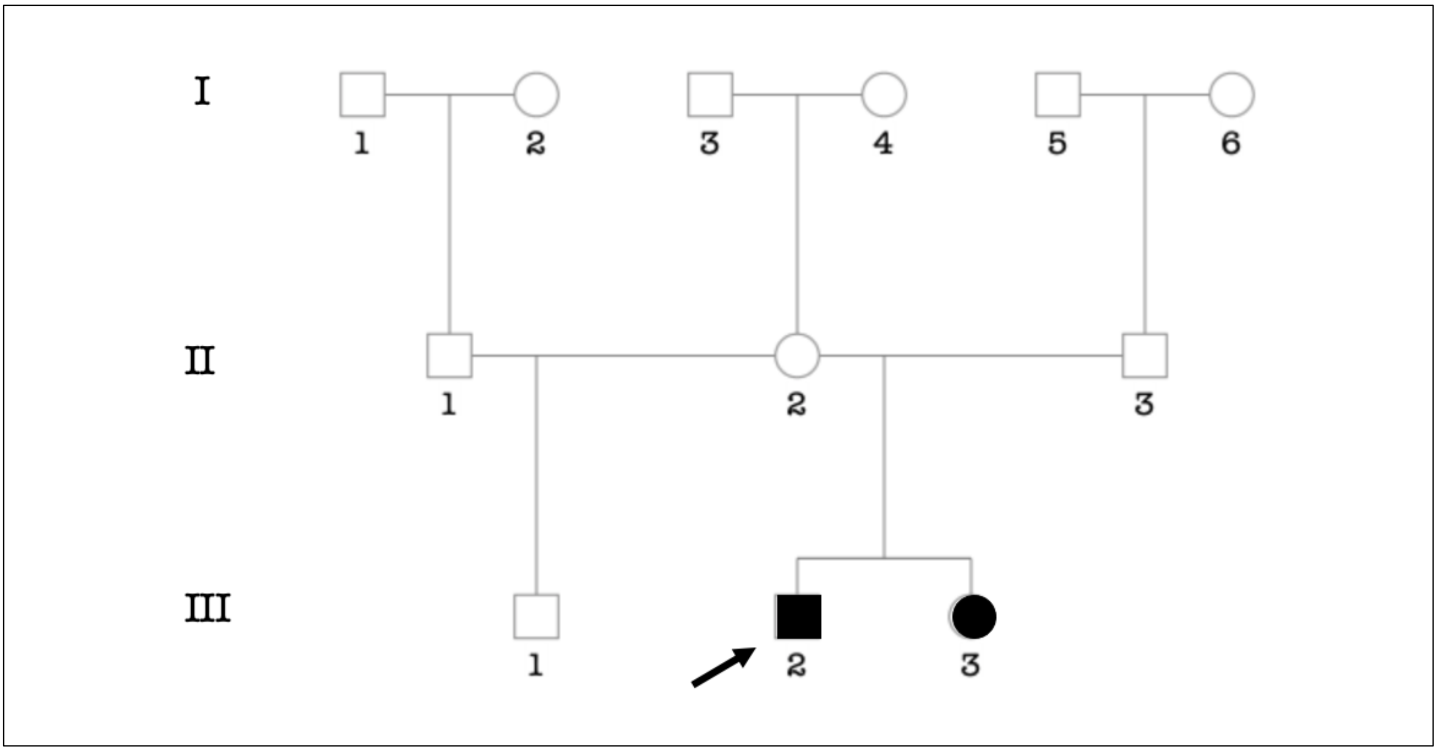

Family24. Showing two affected individuals from a non-consanguineous marriage segregating autosomal recessive non-syndromic hearing impairment

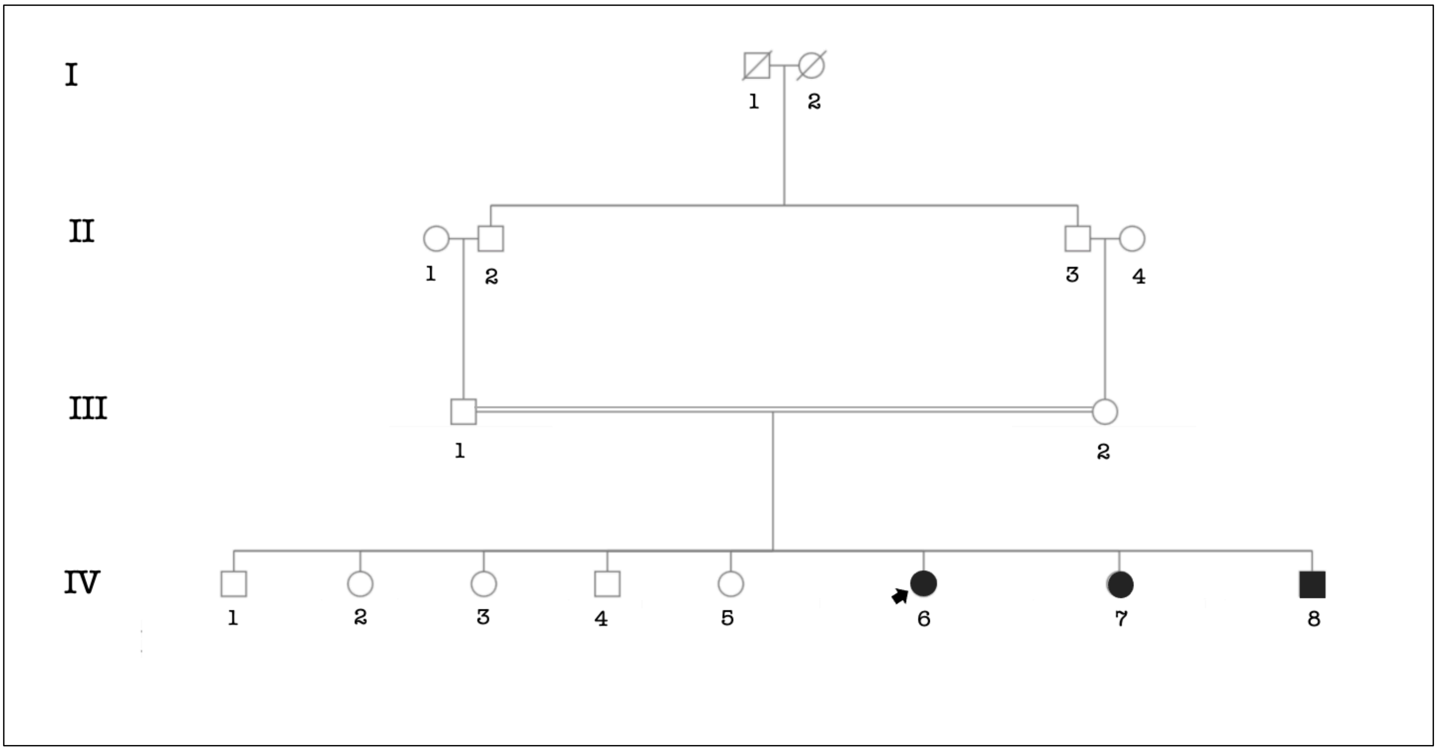

Family25. Showing three affected individuals from a consanguineous marriage segregating autosomal recessive non-syndromic hearing impairment.

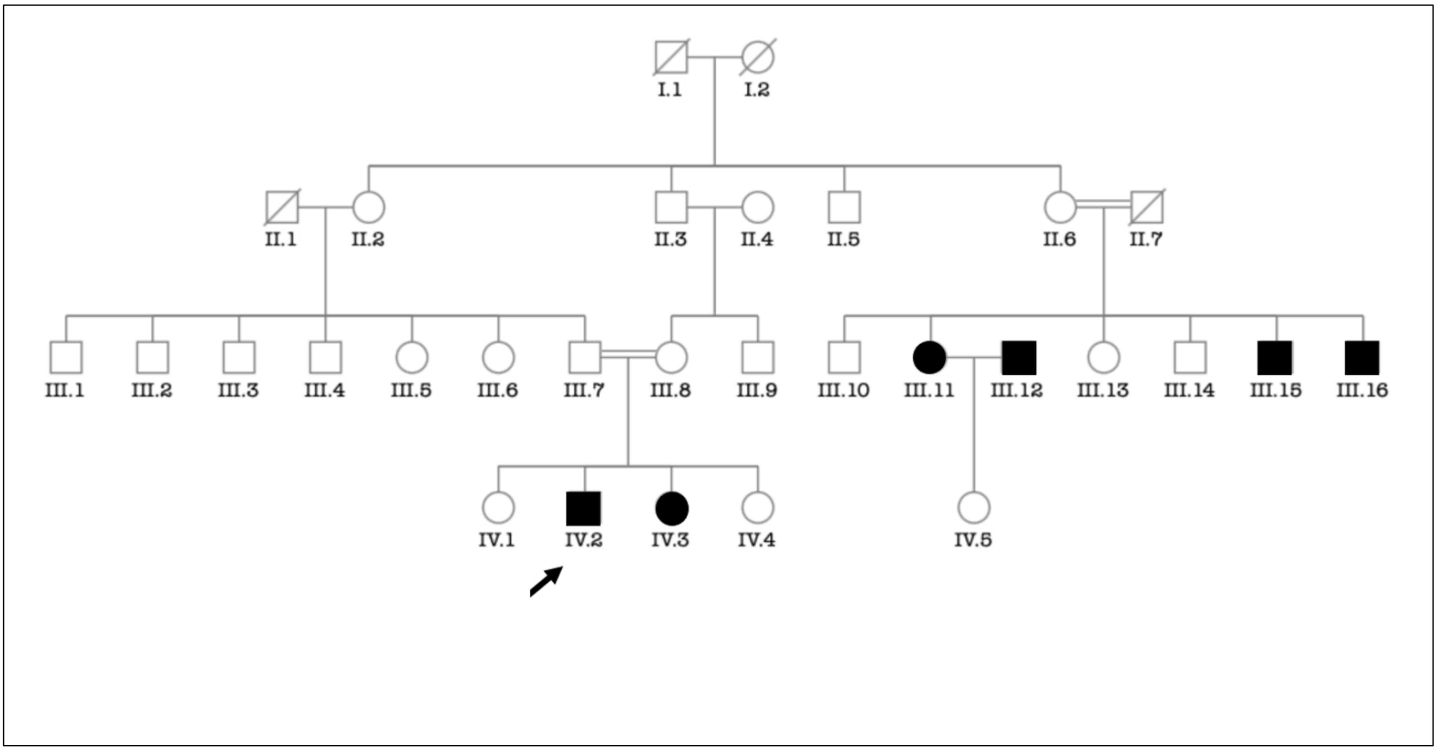

Family26. Showing six affected individuals from consanguineous marriages segregating autosomal recessive non-syndromic hearing impairment.

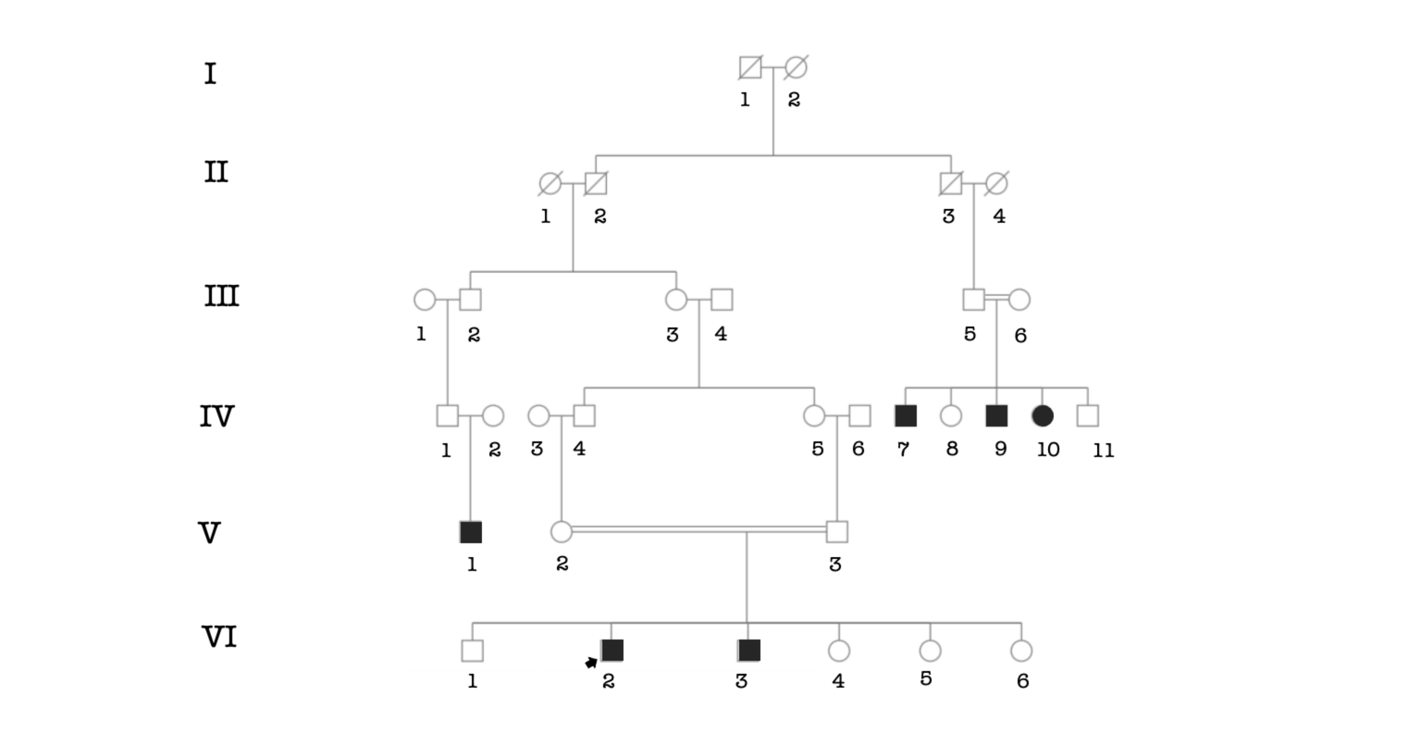

Family27. Showing six affected individuals from consanguineous marriage segregating autosomal recessive non-syndromic hearing impairment.

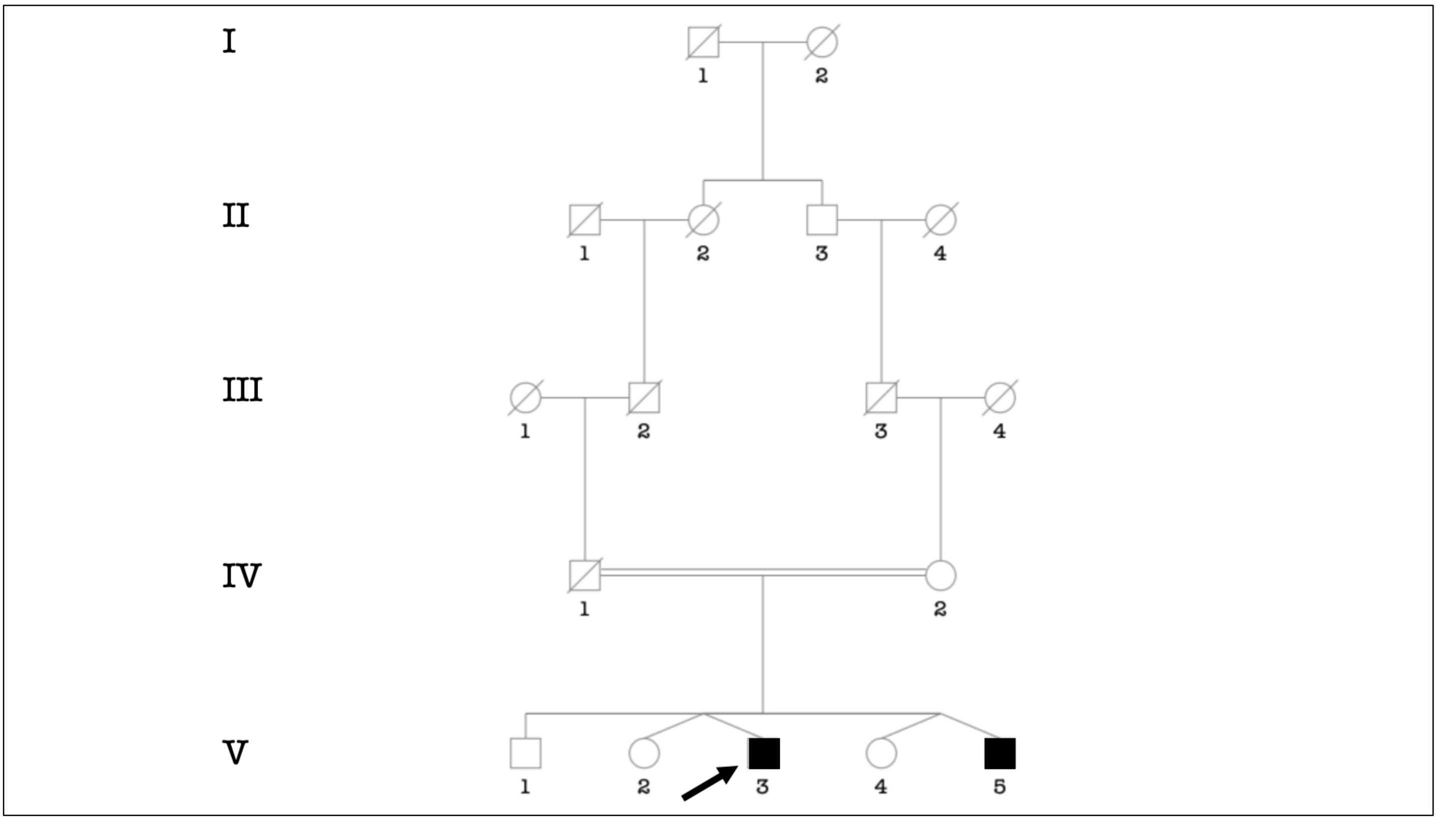

Family28. Showing two affected brothers from a consanguineous marriage segregating autosomal recessive non-syndromic hearing impairment

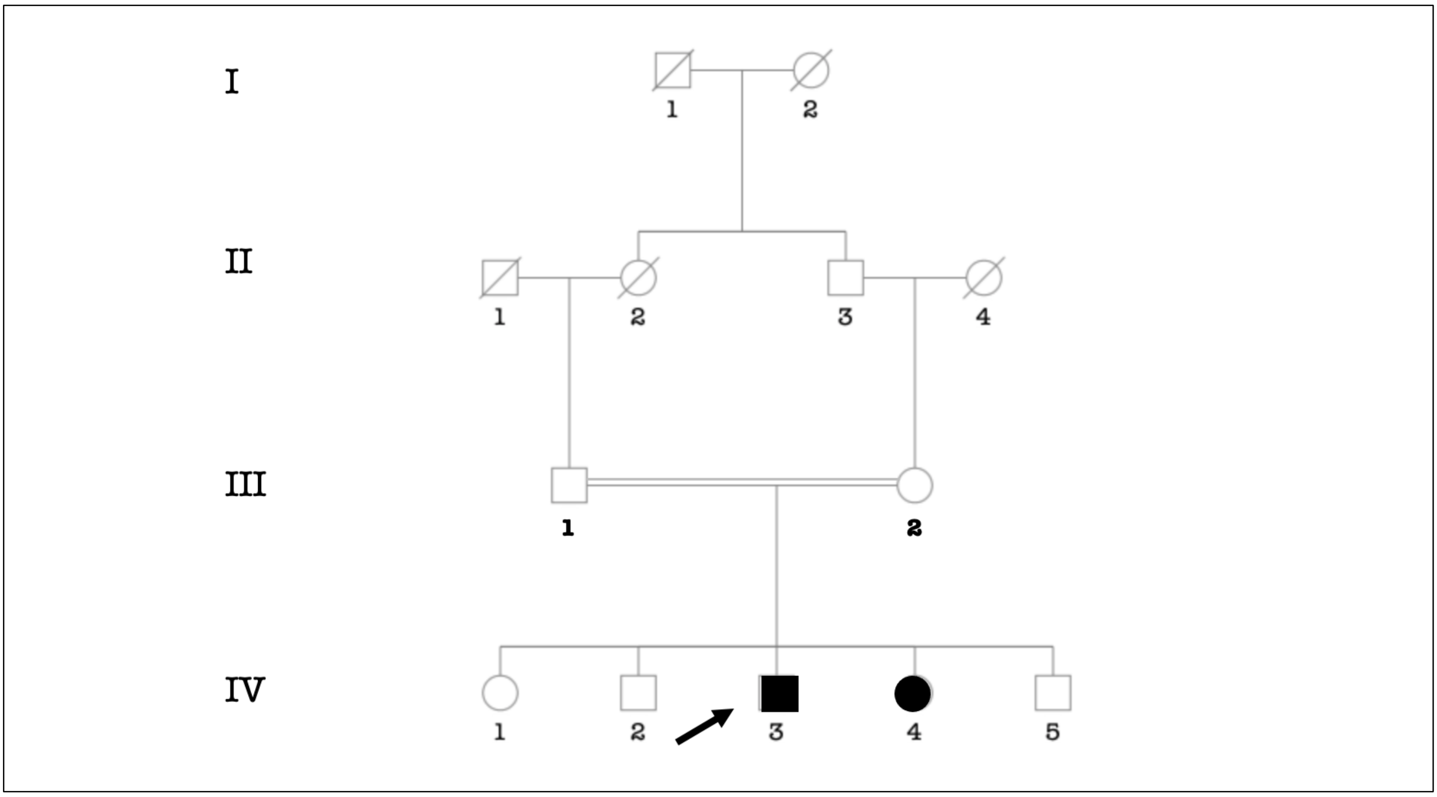

Family29. Showing two affected individuals from a consanguineous marriage segregating autosomal recessive non-syndromic hearing impairment.

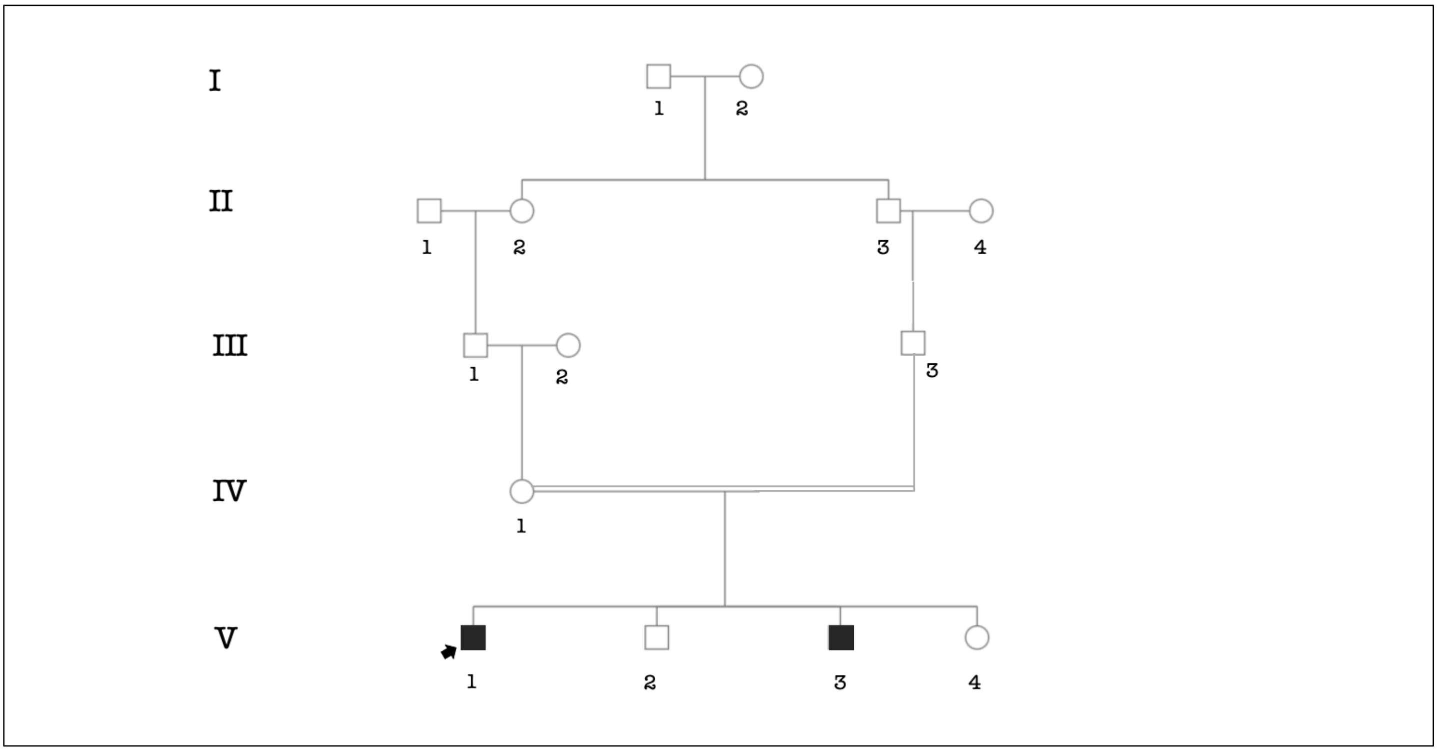

Family30. Showing two affected individuals from a consanguineous marriage segregating likely autosomal recessive non-syndromic hearing impairment.

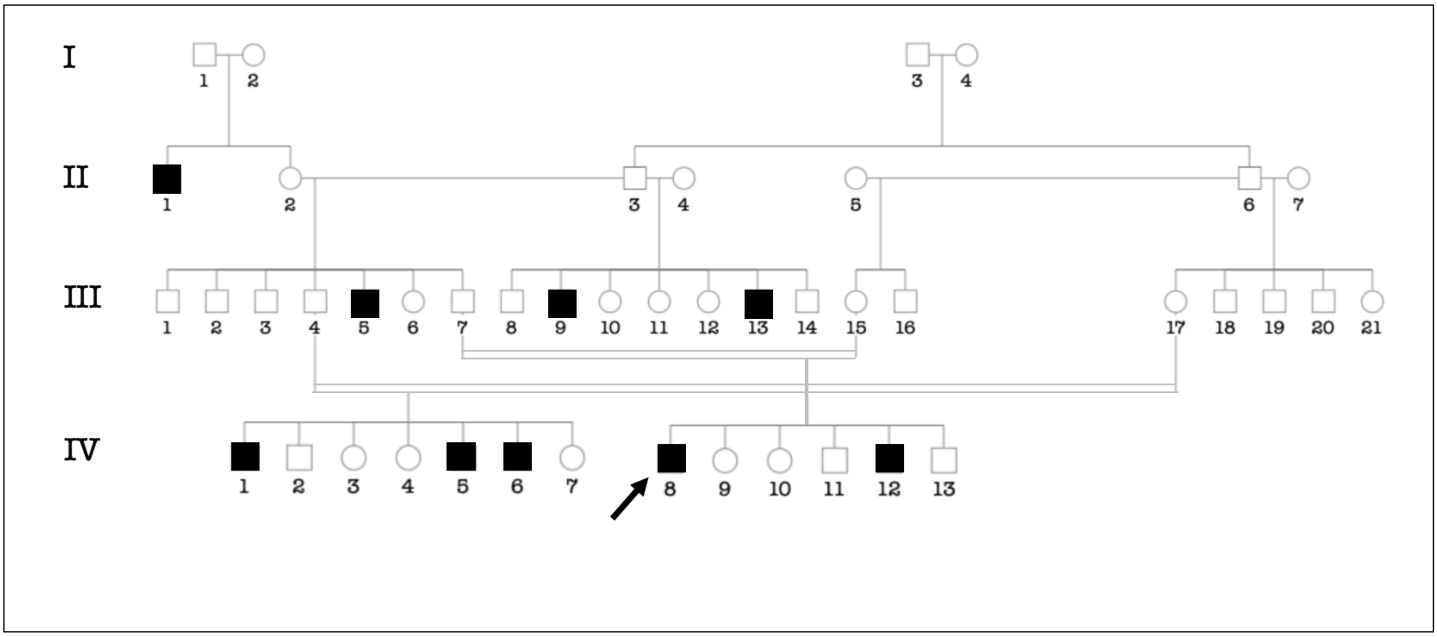

Family31. Showing nine affected boys segregating recessive X-linked non-syndromic hearing impairment.

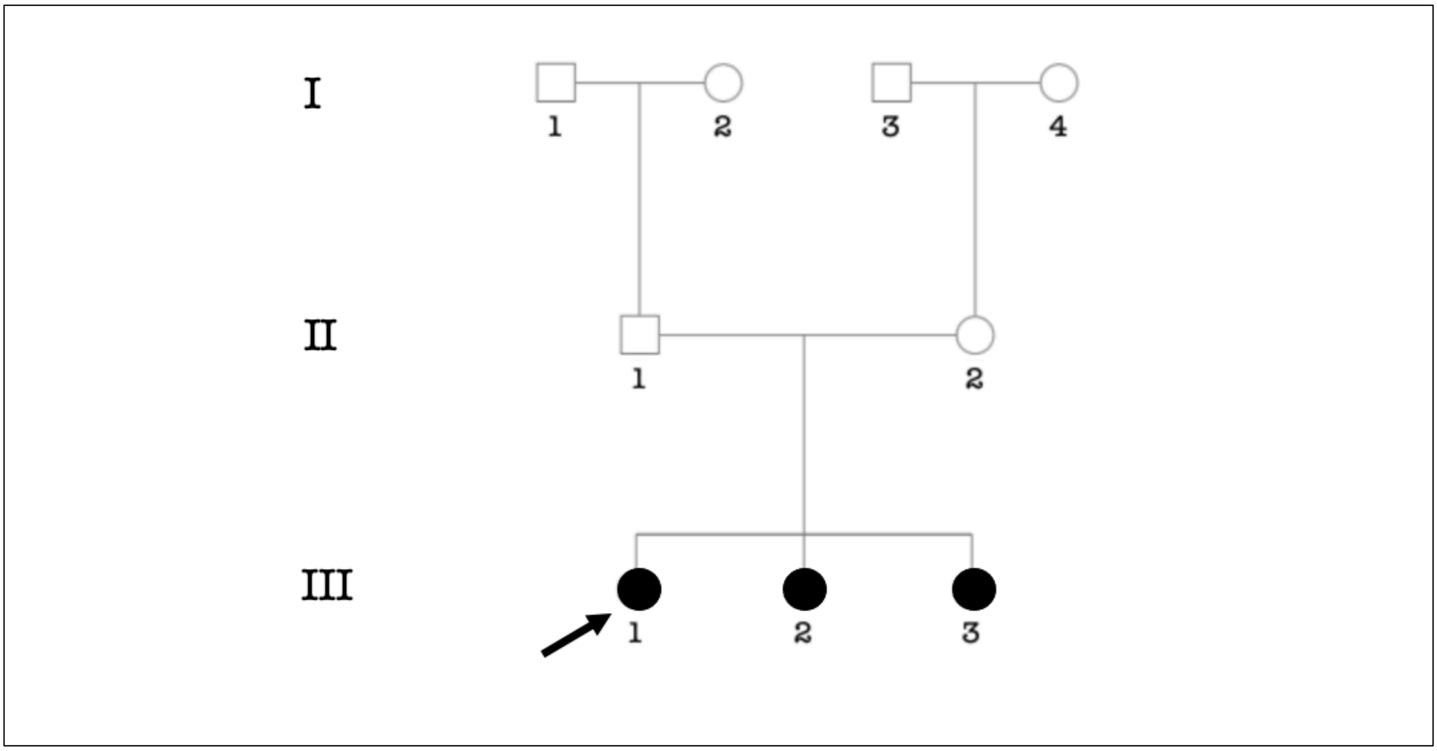

Family32. Showing three affected girls from a non-consanguineous marriage segregating autosomal recessive non-syndromic hearing impairment.

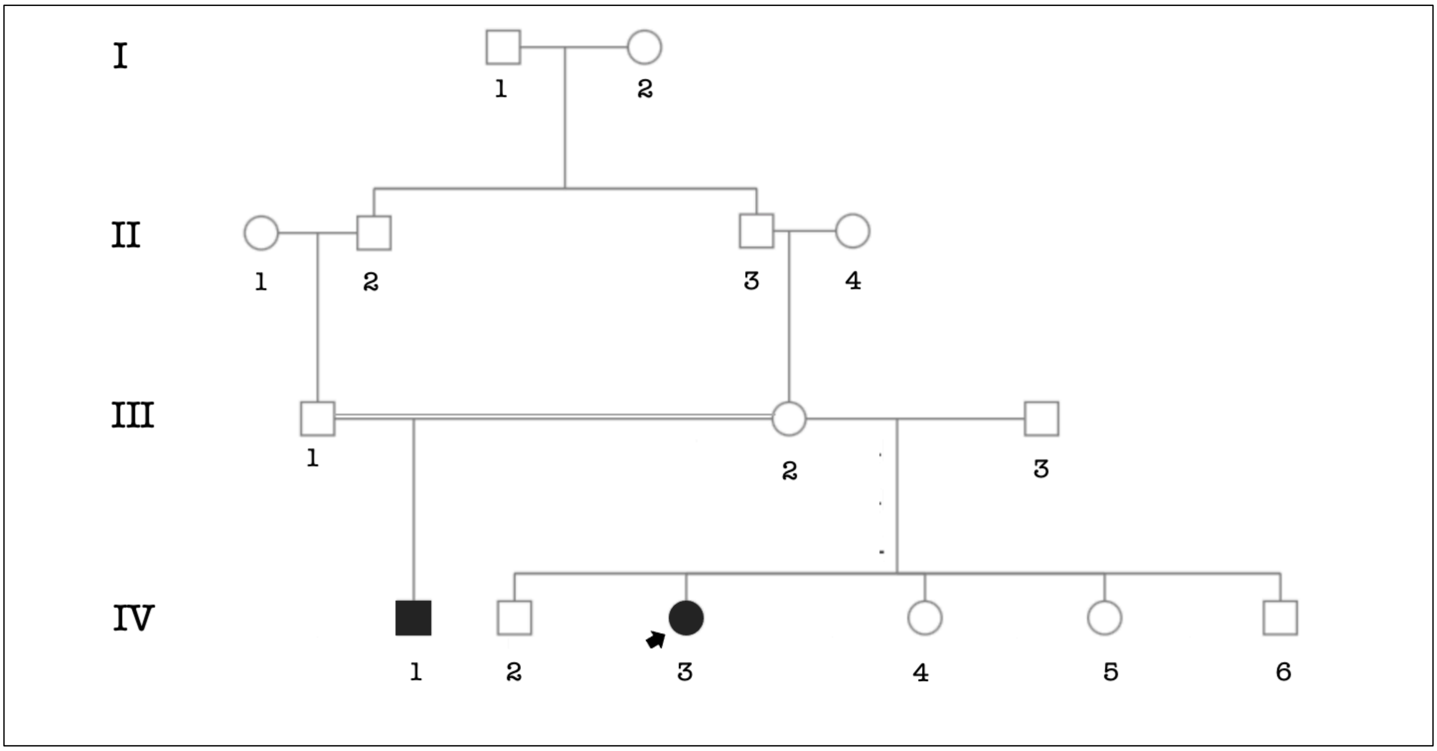

Family33. Showing two affected individuals segregating autosomal recessive non-syndromic hearing impairment.

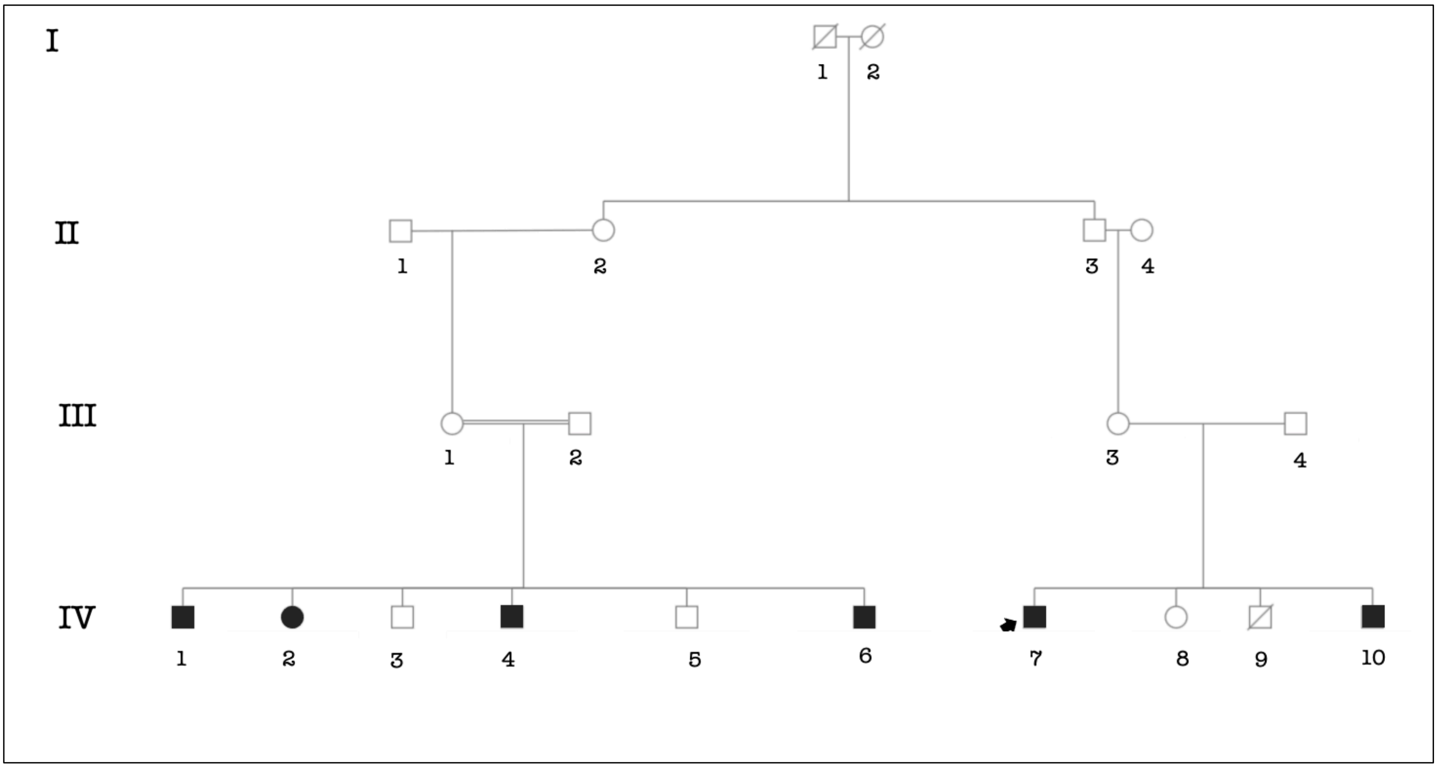

Family34. Showing six affected individuals segregating autosomal recessive non-syndromic hearing impairment.

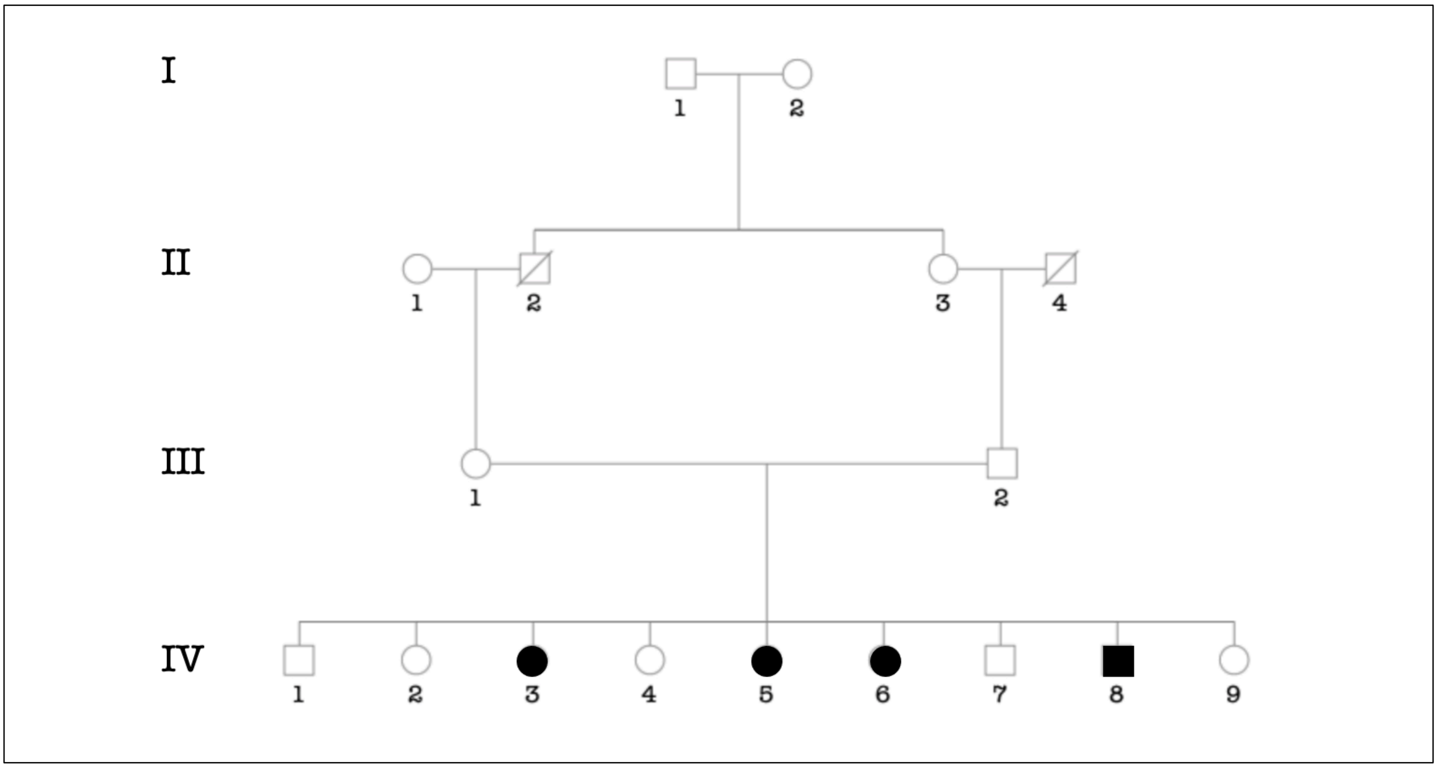

Family35. Showing four affected individuals from a consanguineous marriage segregating Pendred syndrome**.**

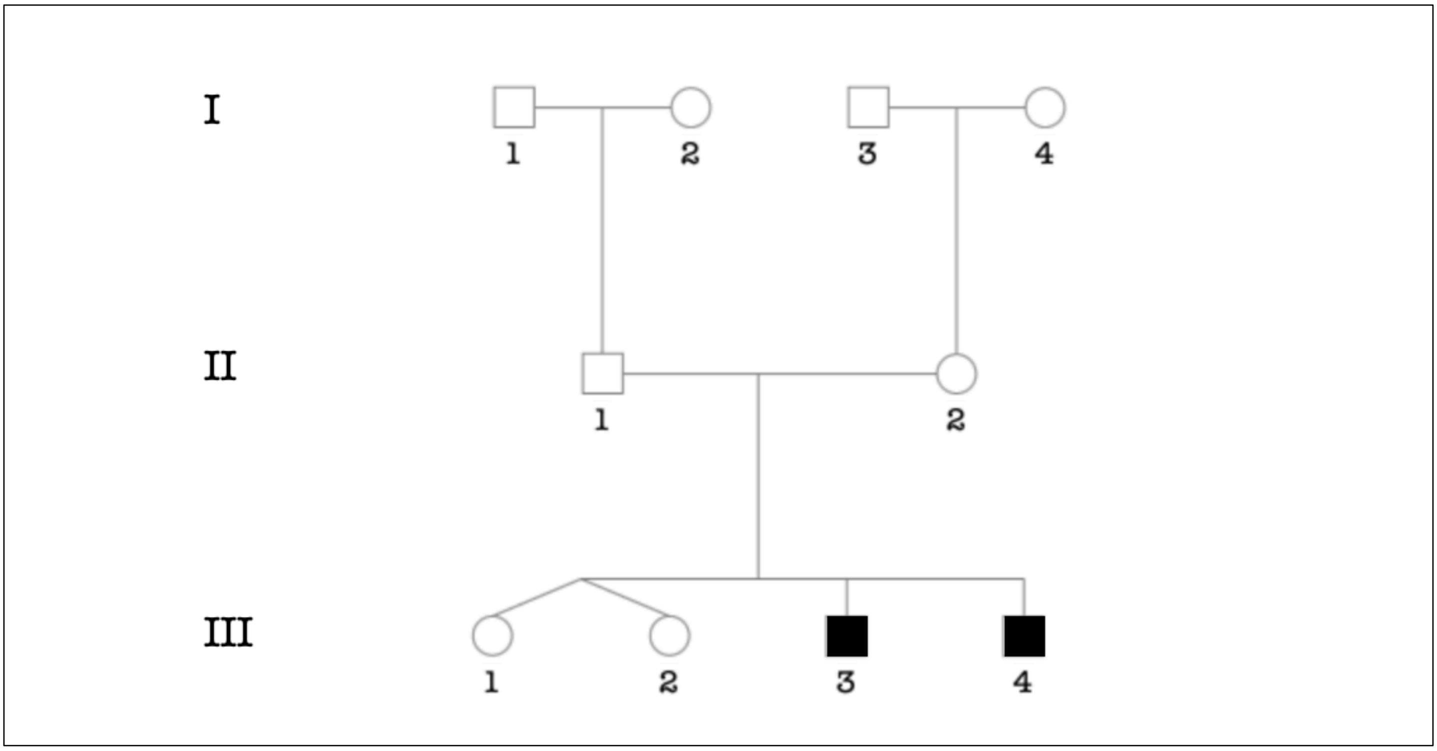

Family36. Showing two affected boys from a non-consanguineous marriage segregating Waardenburg syndrome.

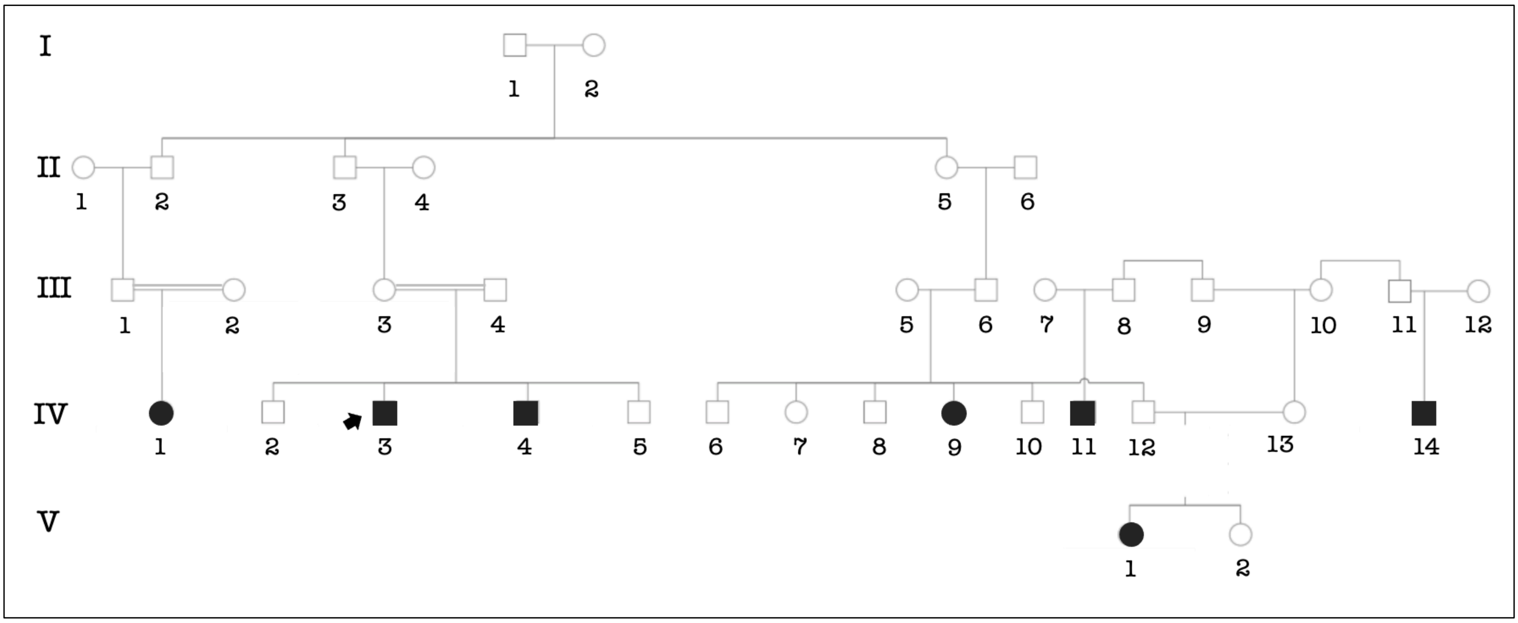

Family37. Showing seven affected individuals from consanguineous and non-consanguineous marriages segregating non-syndromic hearing impairment.

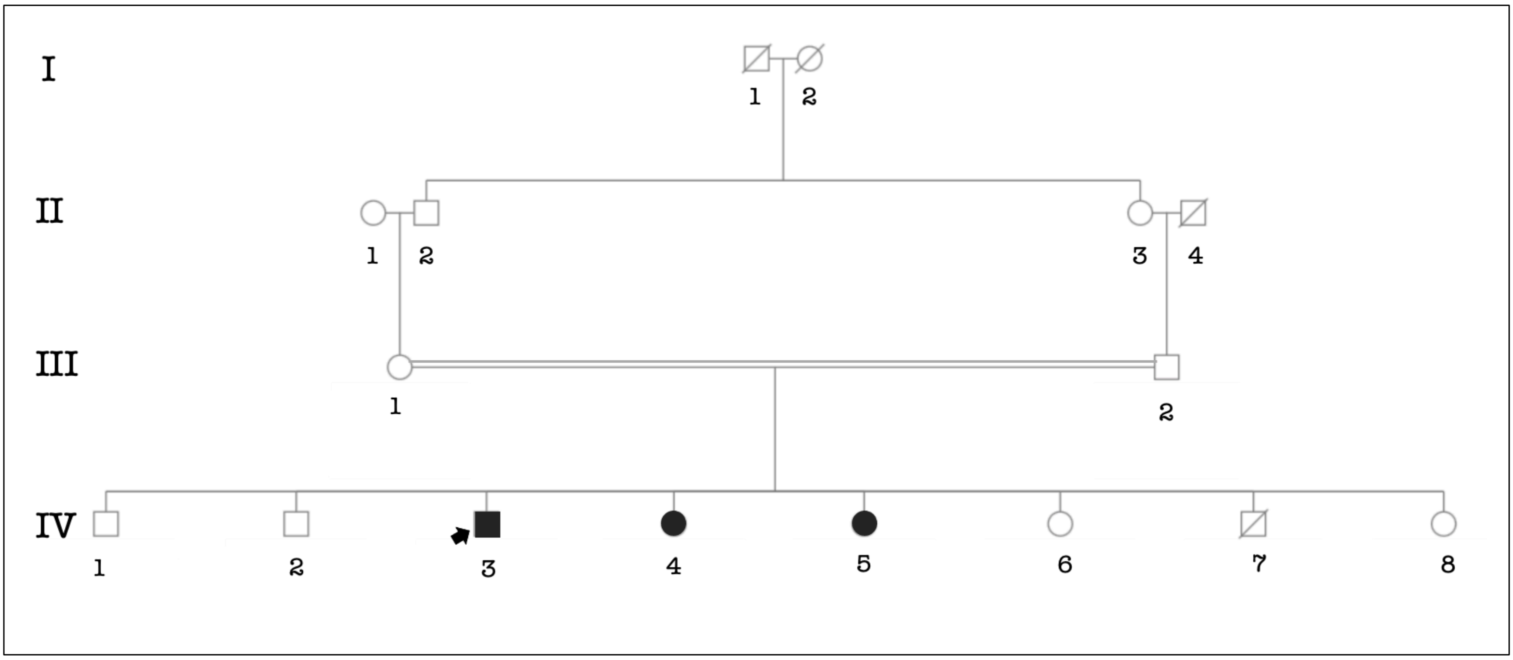

Family38. Showing three affected individuals from a consanguineous marriage segregating autosomal recessive non-syndromic hearing impairment.

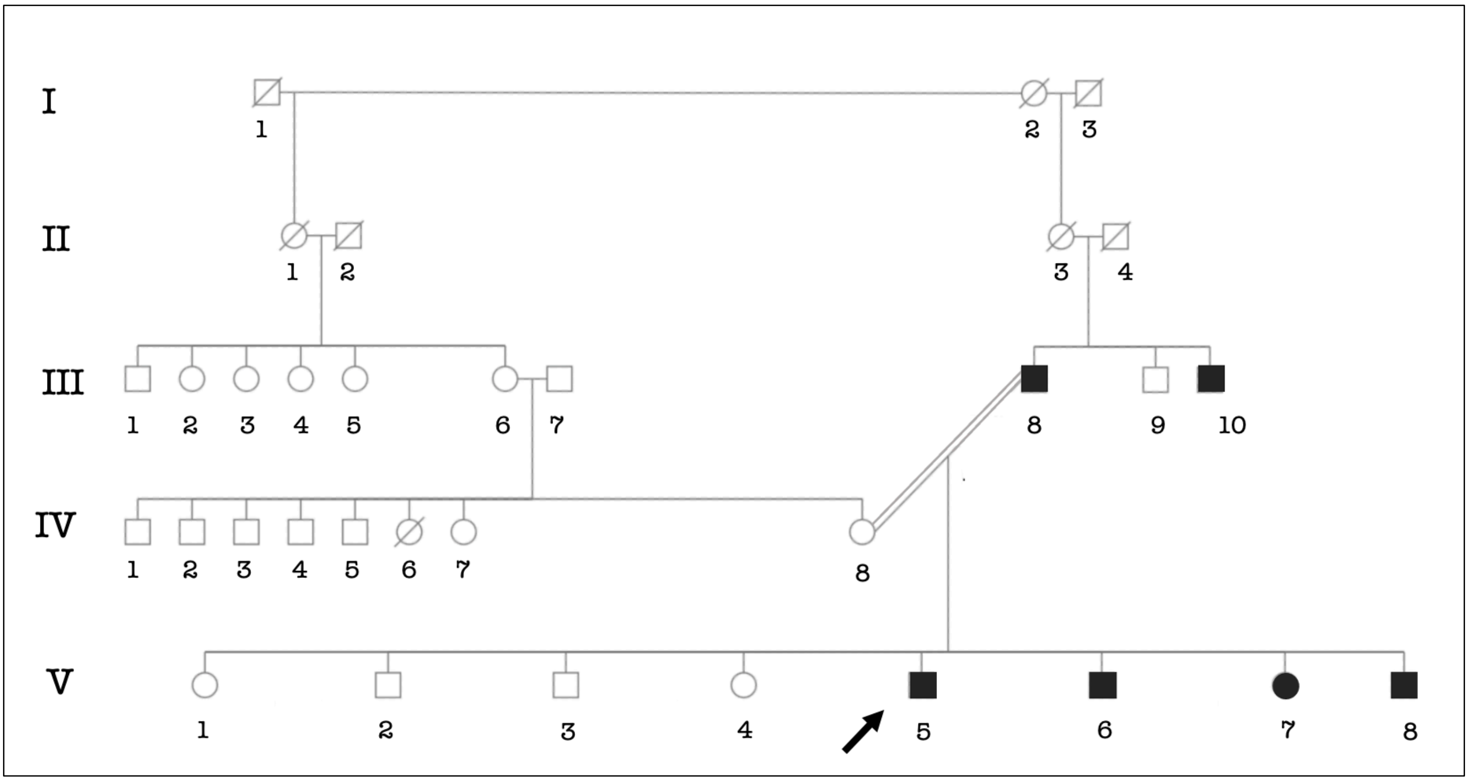

Family39. Showing six affected individuals segregating autosomal recessive non-syndromic hearing impairment.

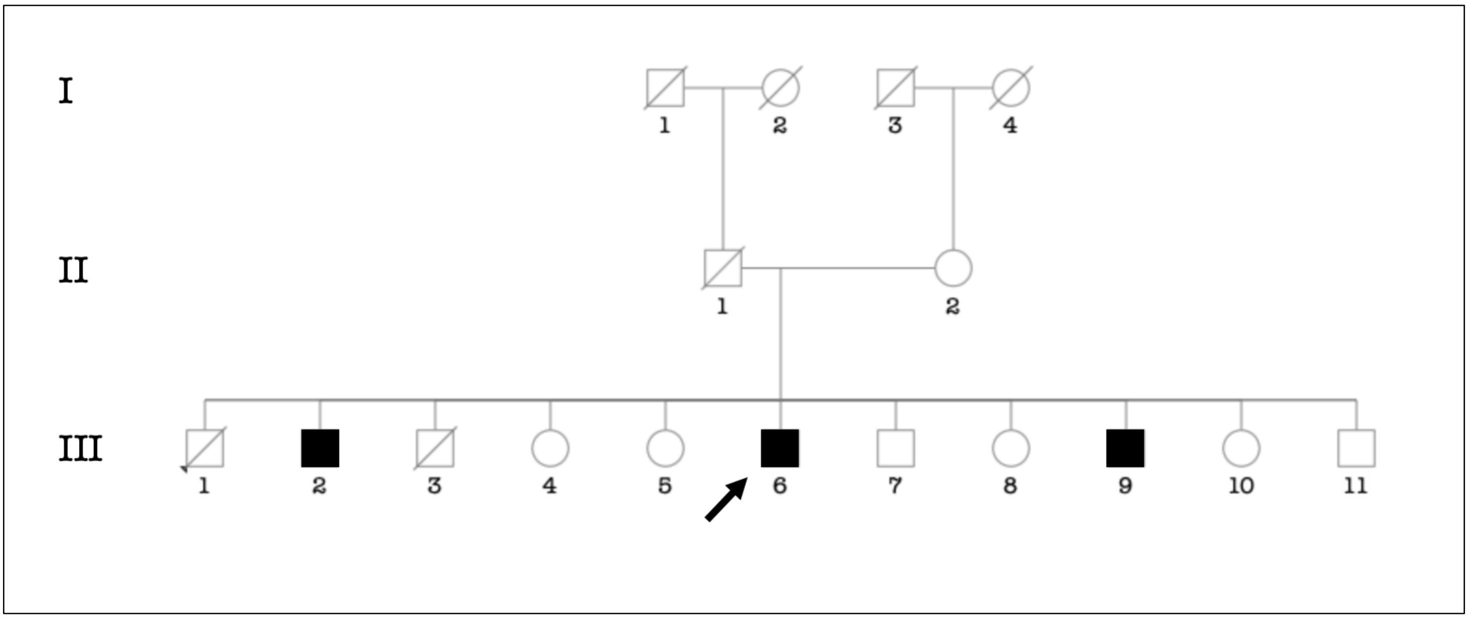

Family40. Showing three affected boys from a non-consanguineous marriage segregating likely recessive X-linked non-syndromic hearing impairment.

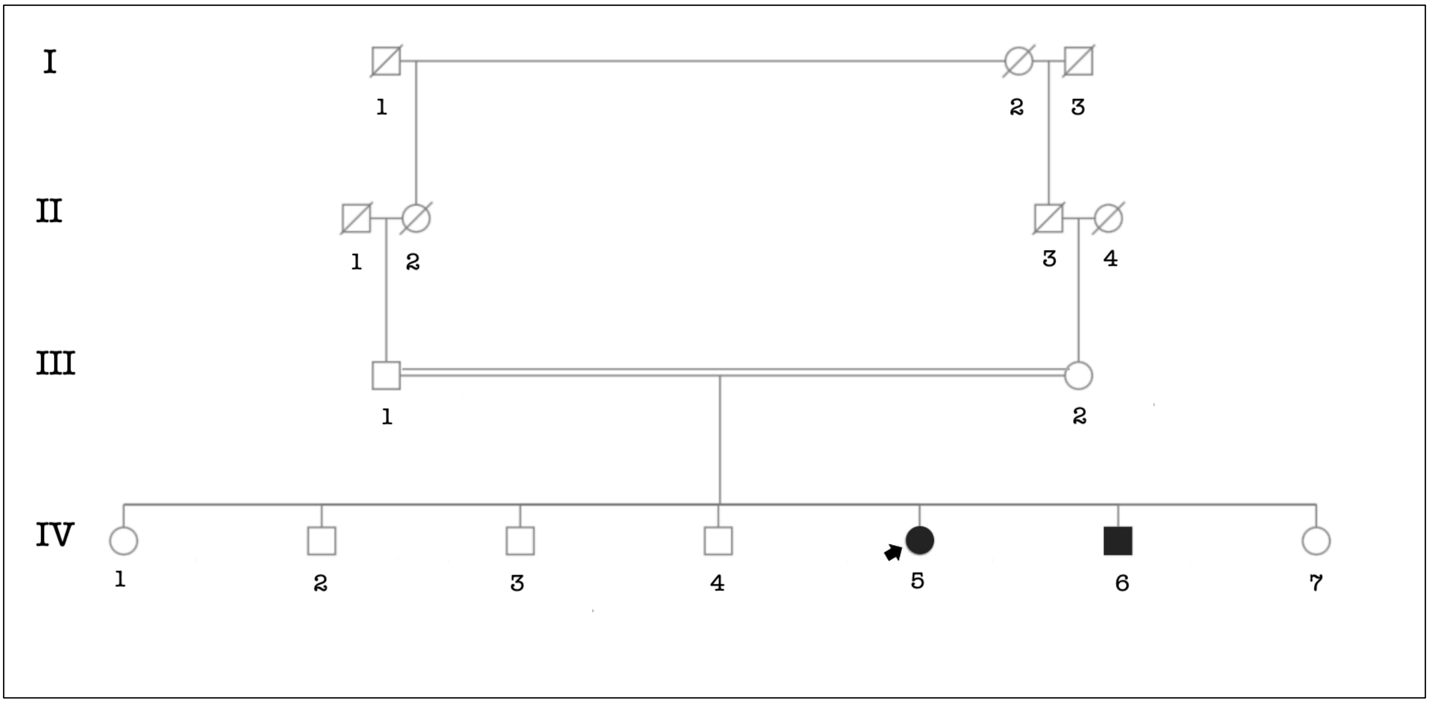

Family41. Pedigree showing two affected individuals from a consanguineous marriage segregating autosomal recessive non-syndromic hearing impairment.

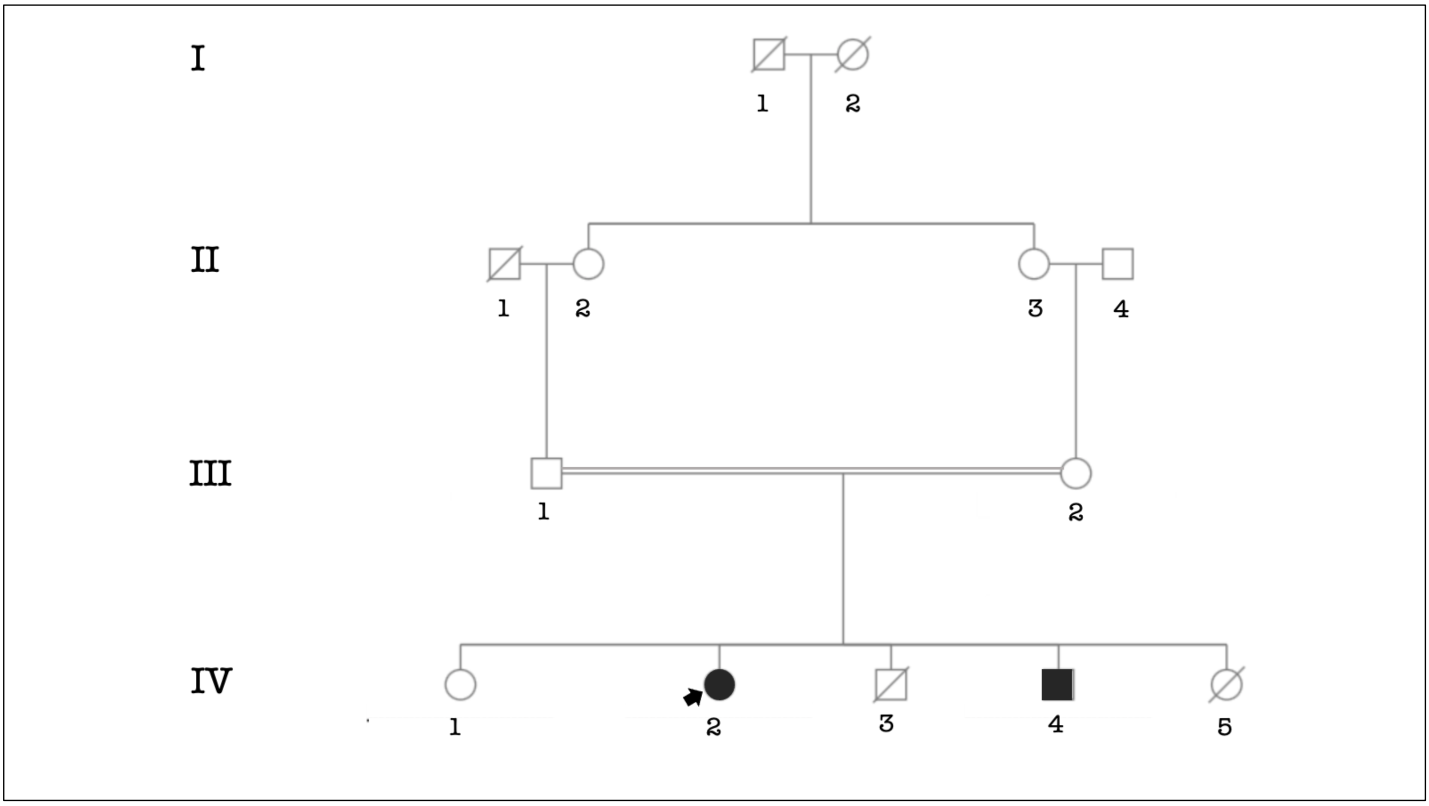

Family42. Pedigree showing two affected individuals from a consanguineous marriage segregating autosomal recessive non-syndromic hearing impairment.

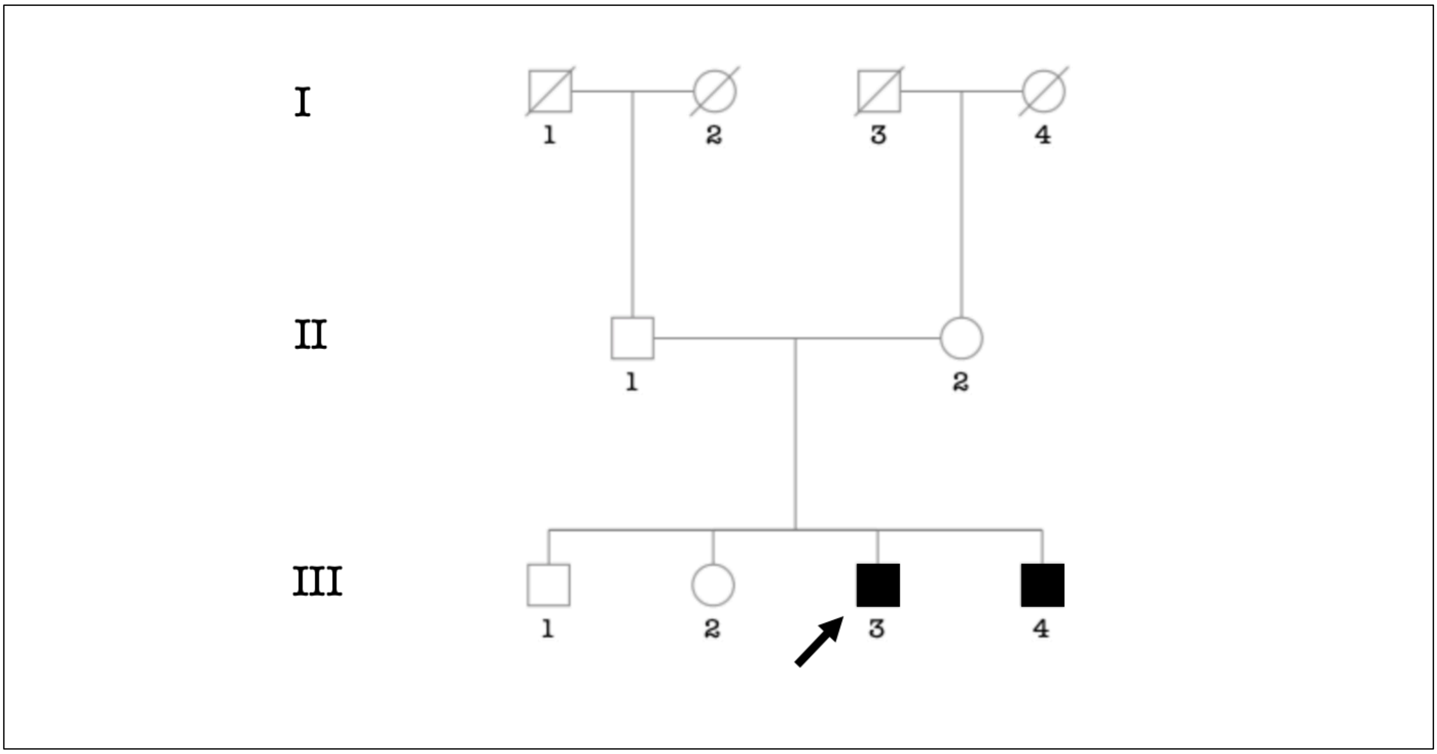

Family43. Pedigree showing two affected individuals from a consanguineous marriage segregating autosomal recessive non-syndromic hearing impairment.

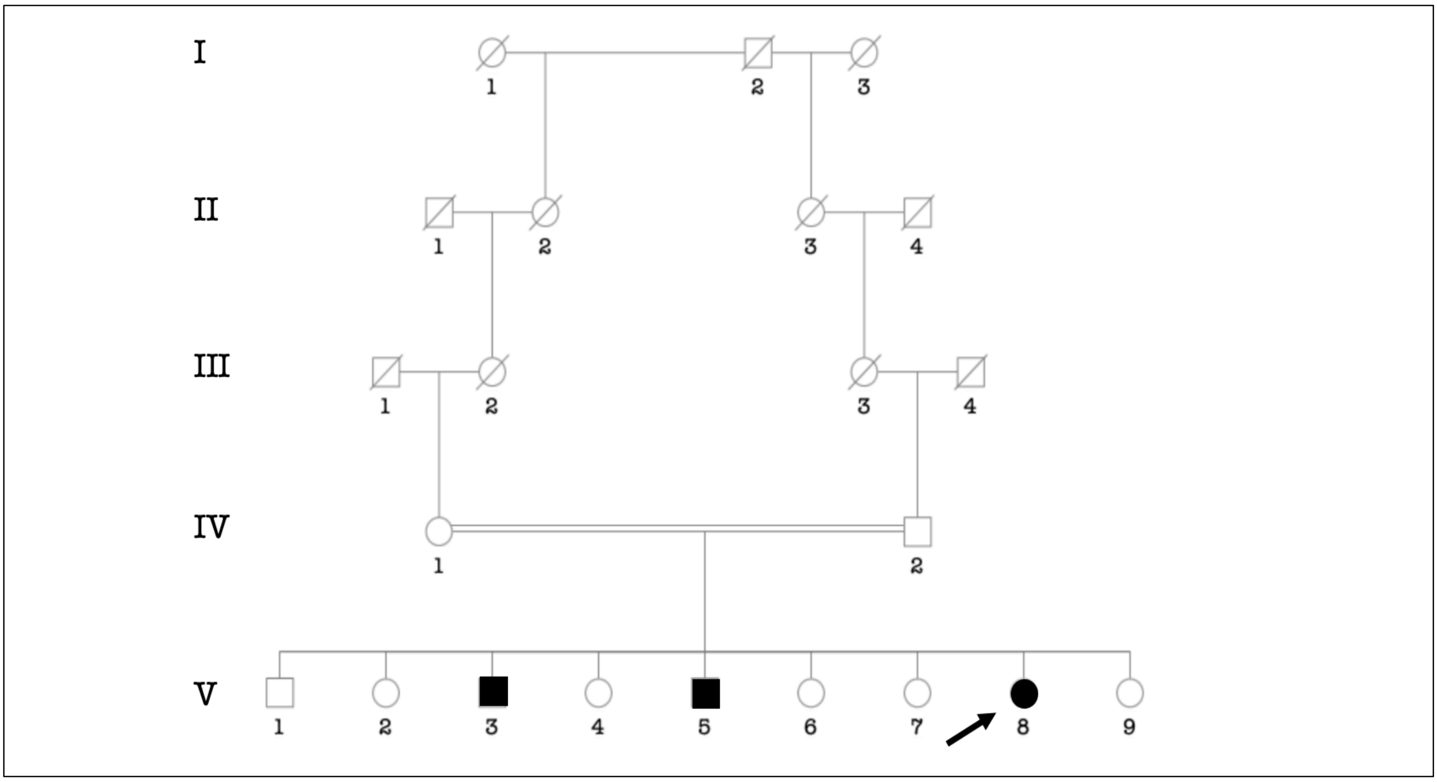

Family44. Pedigree showing two affected individuals from a consanguineous marriage segregating Usher syndrome type2.

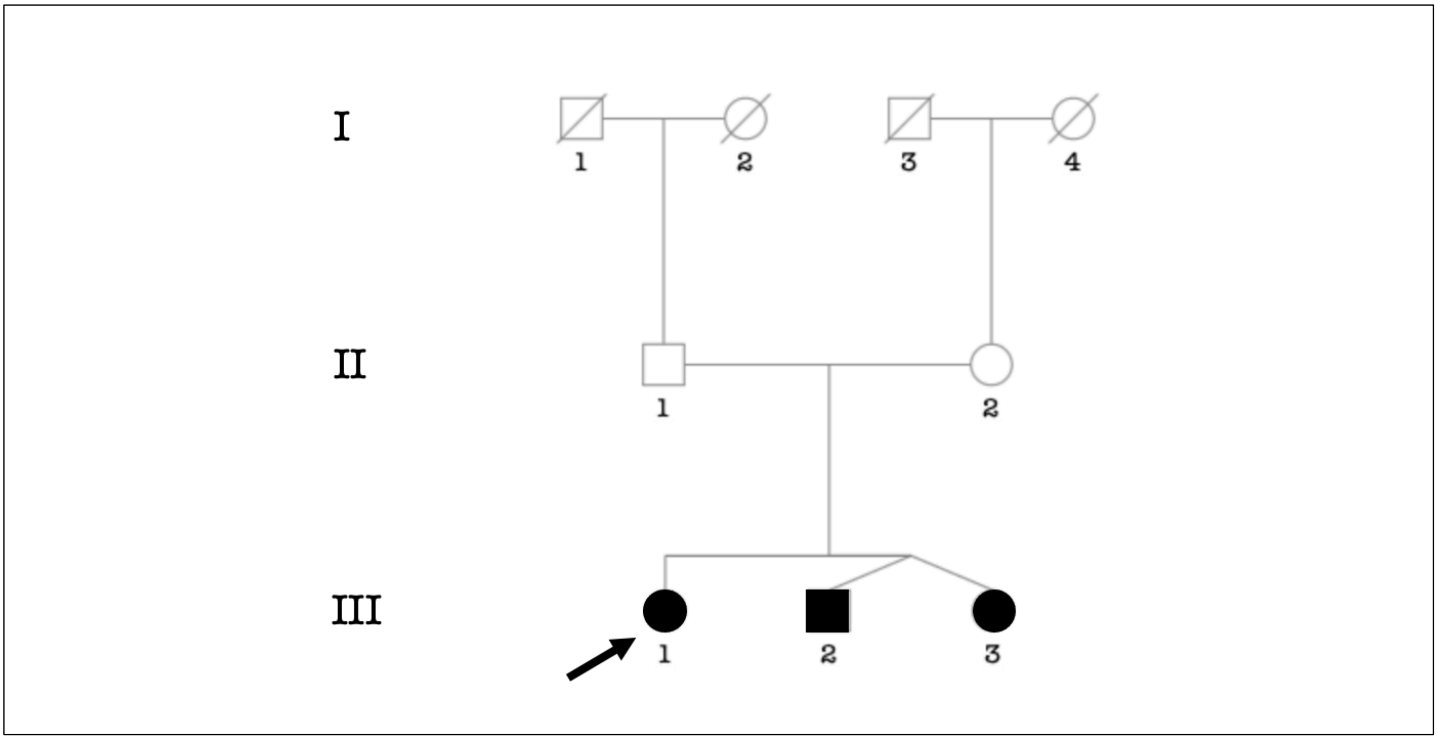

Family45. Pedigree showing three affected individuals from a non-consanguineous marriage segregating autosomal recessive non-syndromic hearing impairment.

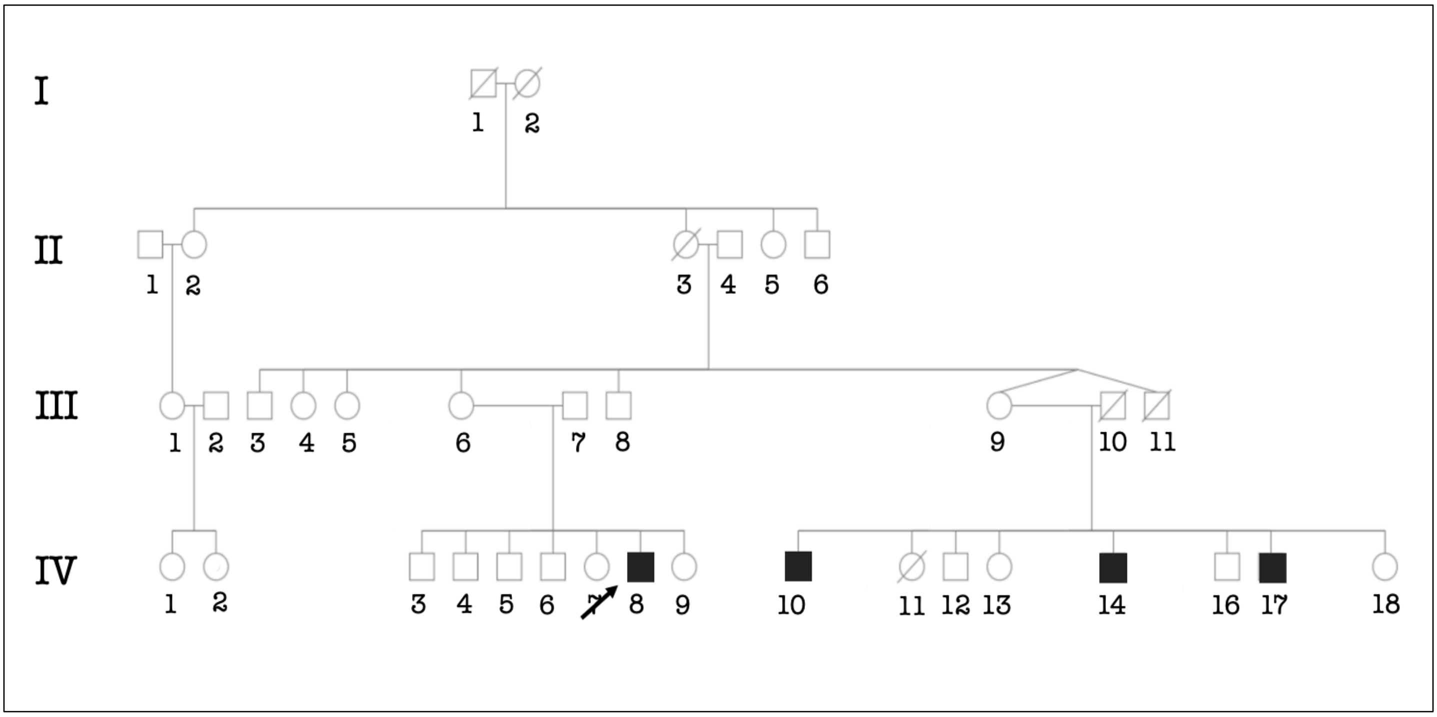

Family46. Pedigree showing four affected boys segregating likely recessive autosomal recessive non-syndromic hearing impairment.

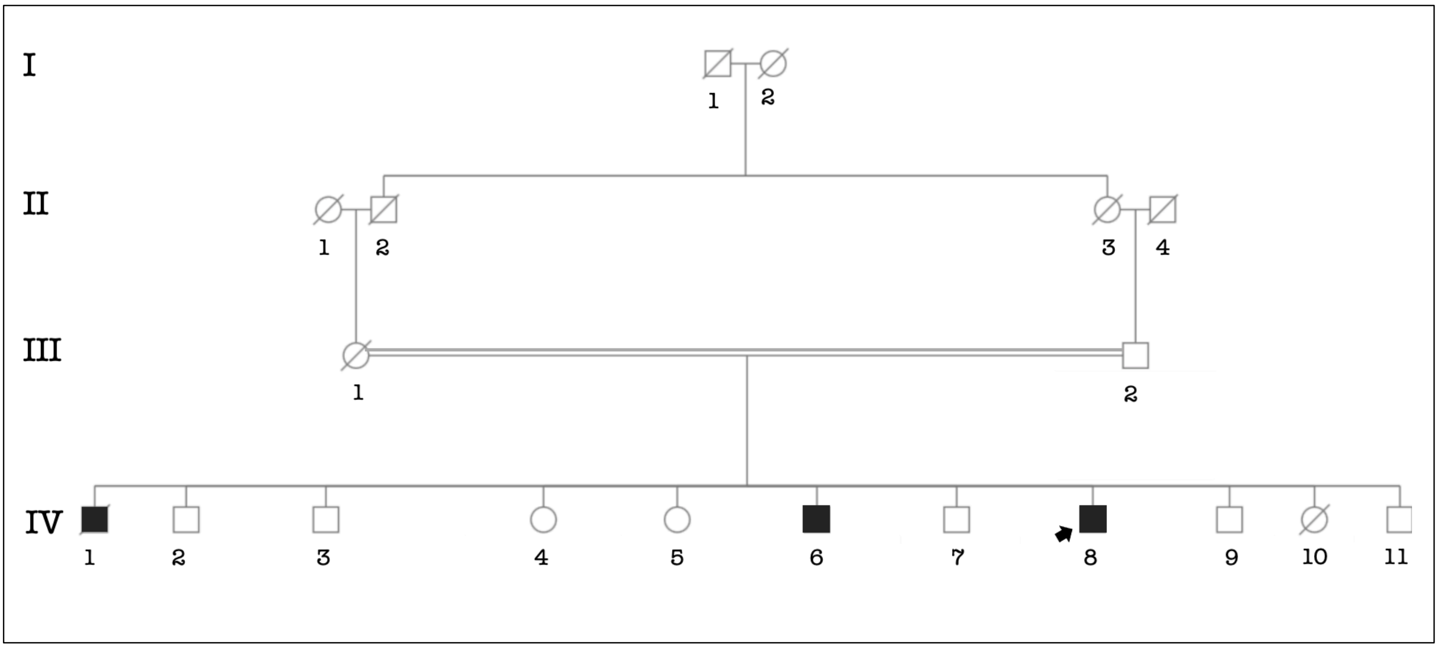

Family47. Pedigree showing three affected individuals from a consanguineous marriage segregating autosomal recessive non-syndromic hearing impairment.

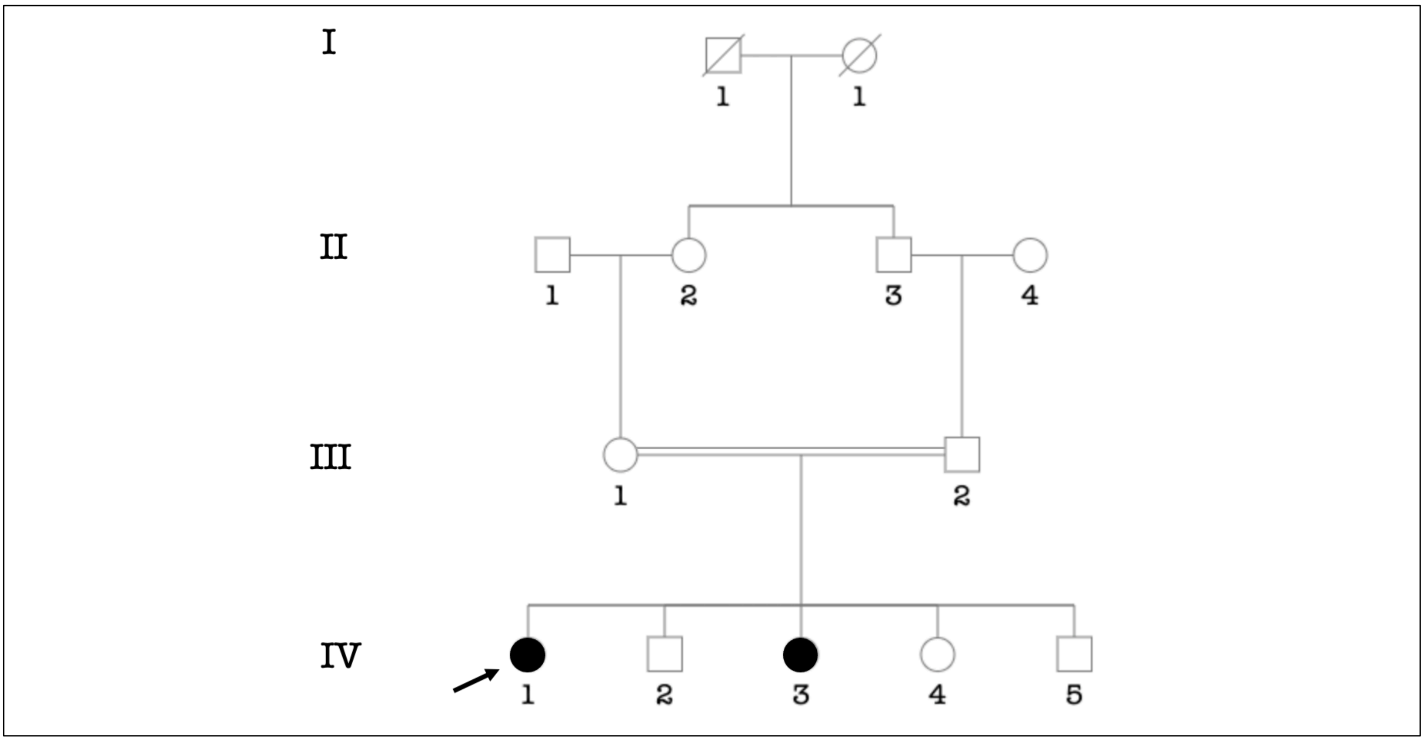

Family48. Pedigree showing two affected girls from a consanguineous marriage segregating autosomal recessive non-syndromic hearing impairment

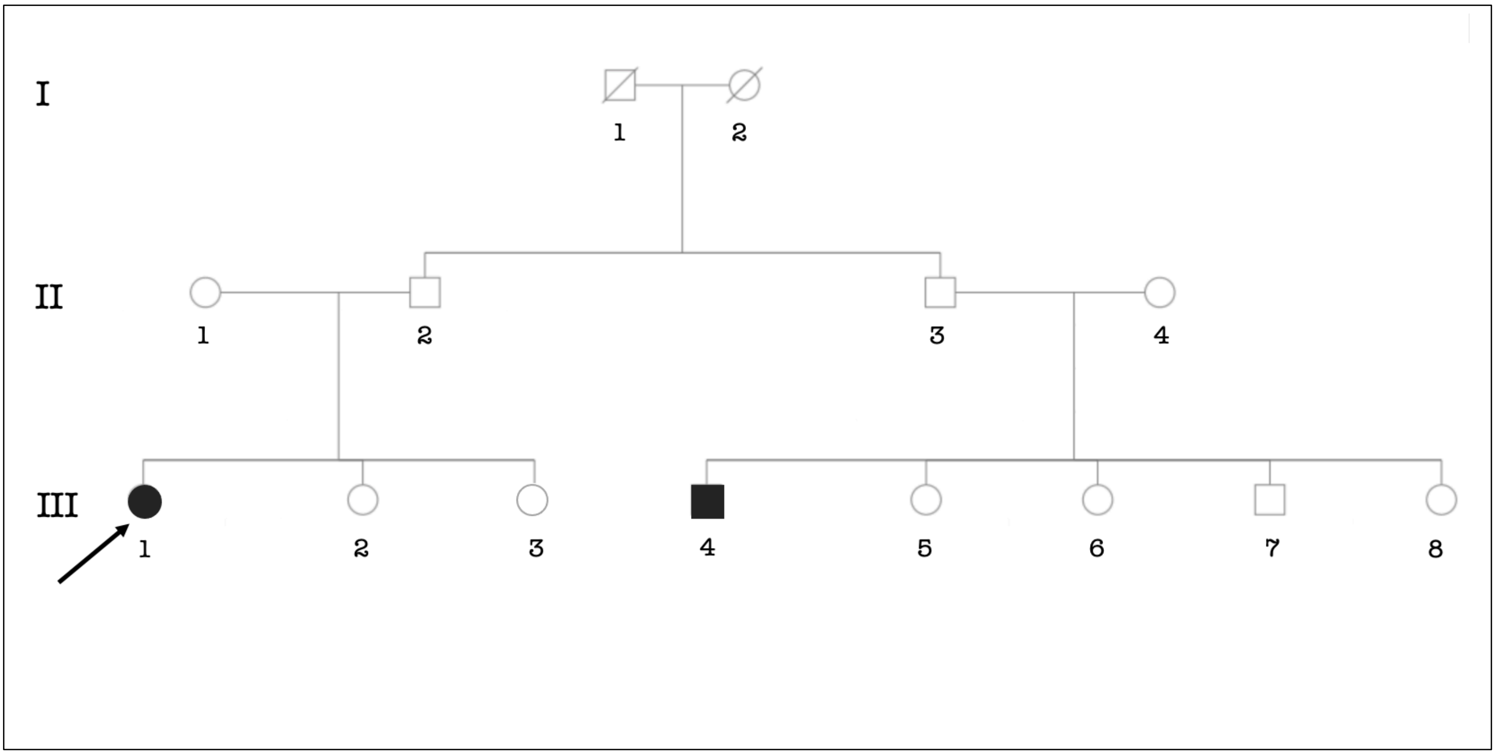

Family49. Pedigree showing two affected individuals from a non-consanguineous marriage segregating autosomal recessive non-syndromic hearing impairment

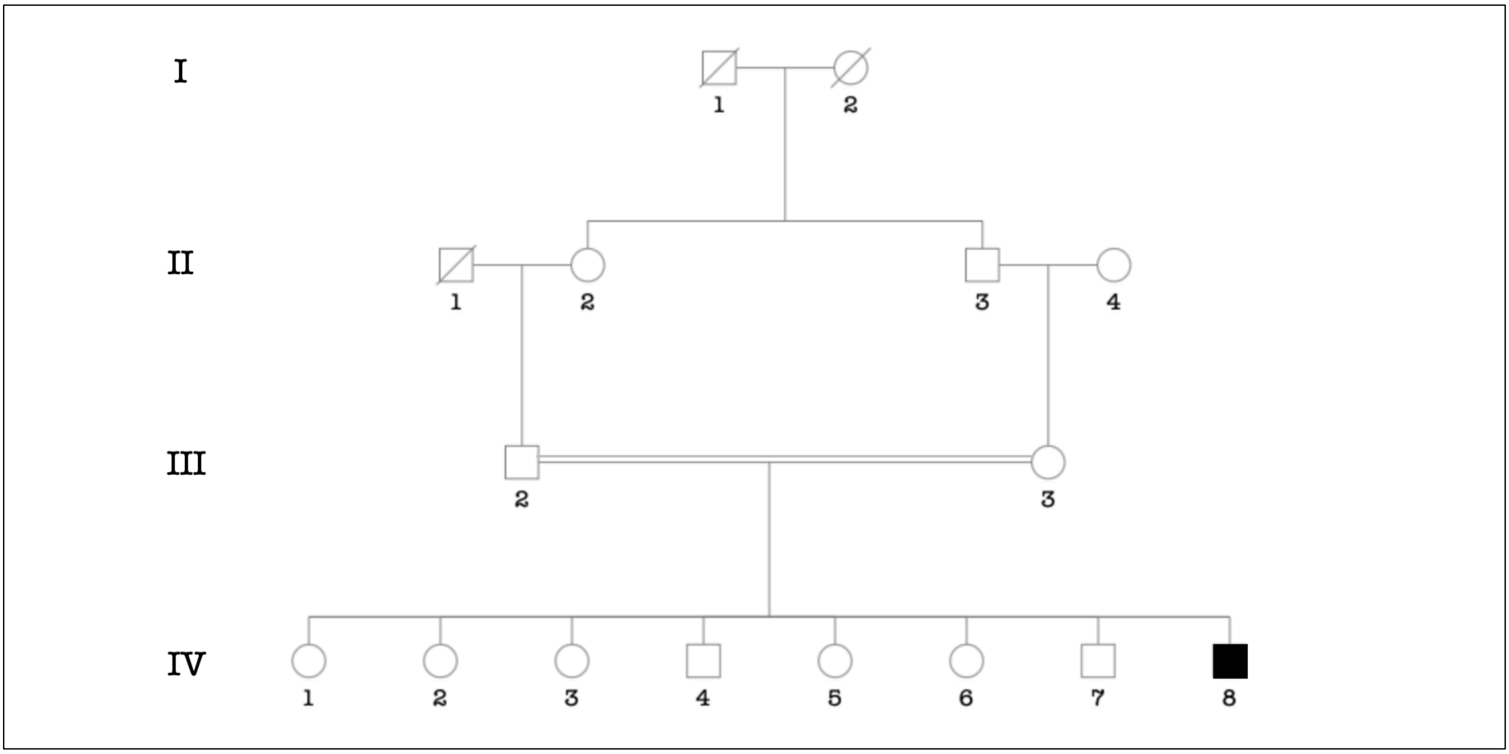

Family50. Pedigree showing a proband with Waardenburg Syndrome type2.

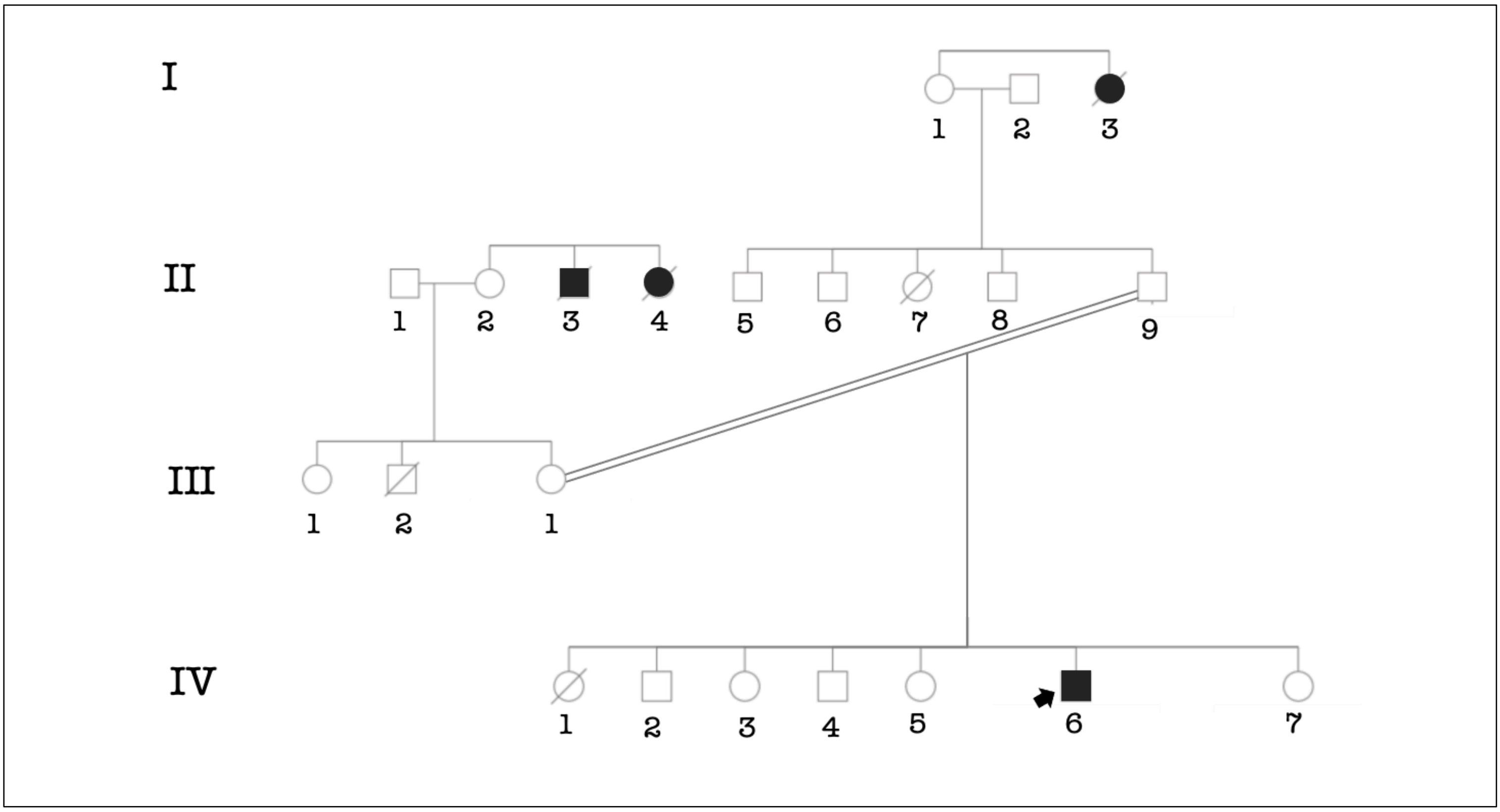

Family51. Pedigree showing four affected individuals segregating autosomal recessive non-syndromic hearing impairment

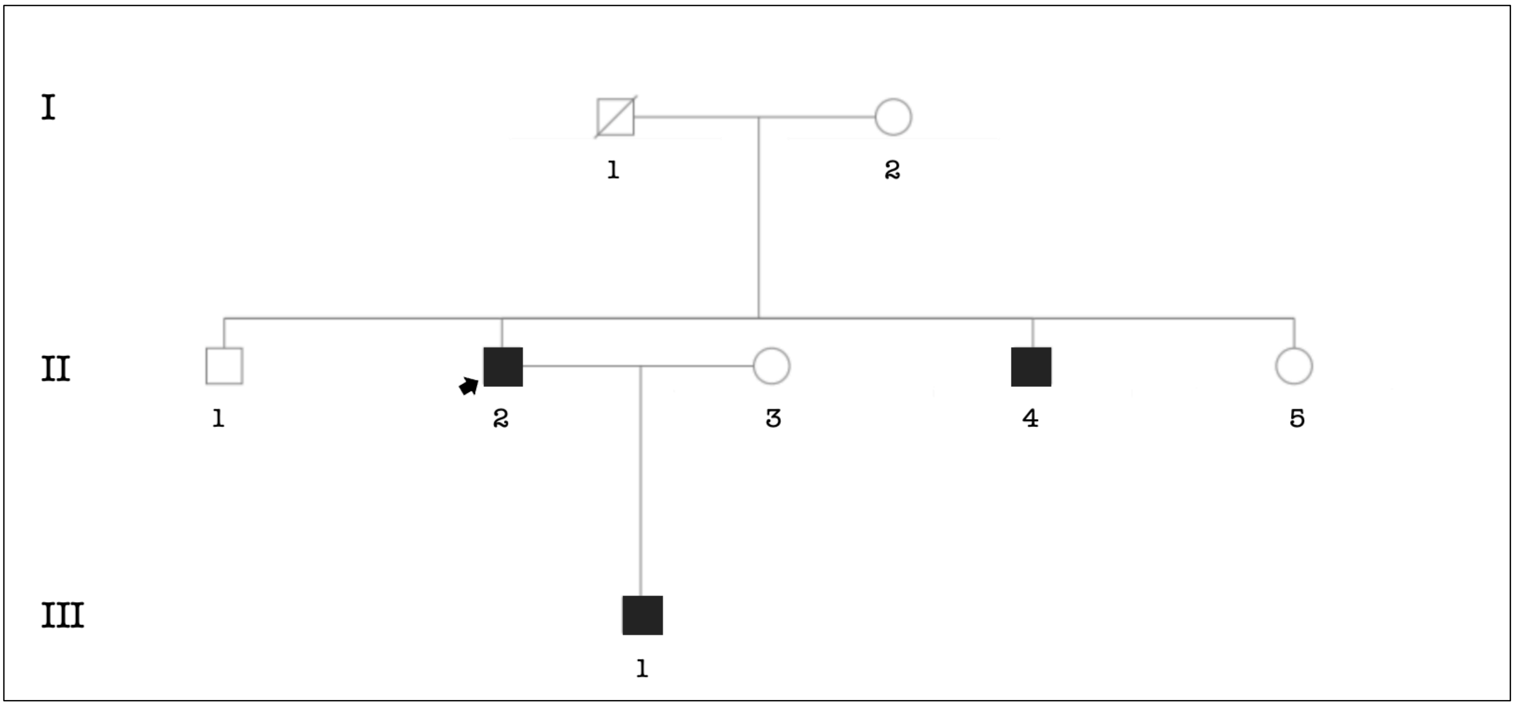

Family52. Pedigree showing three affected individuals segregating Waardenburg syndrome type 2.

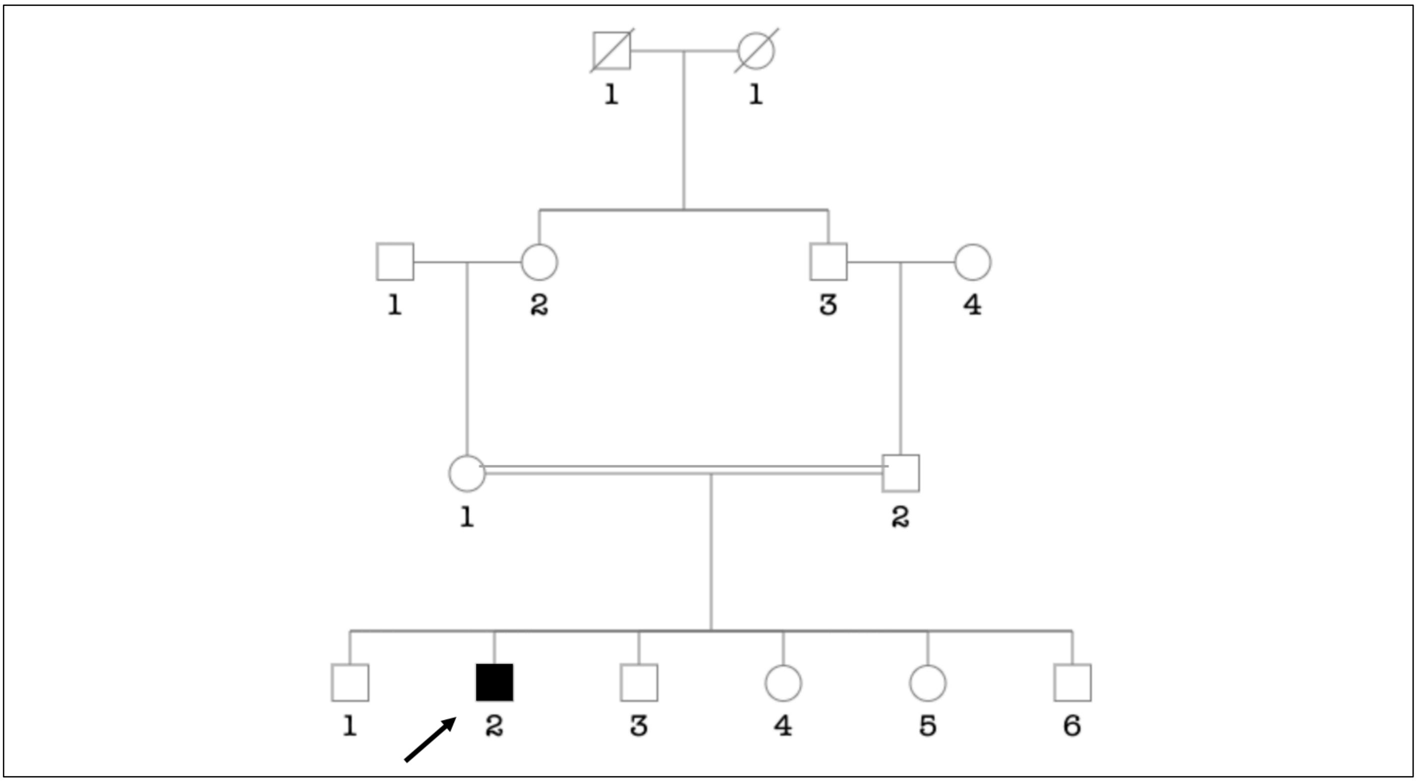

Family53. Pedigree showing a proband indicates by an arrow with non-syndromic hearing impairment.

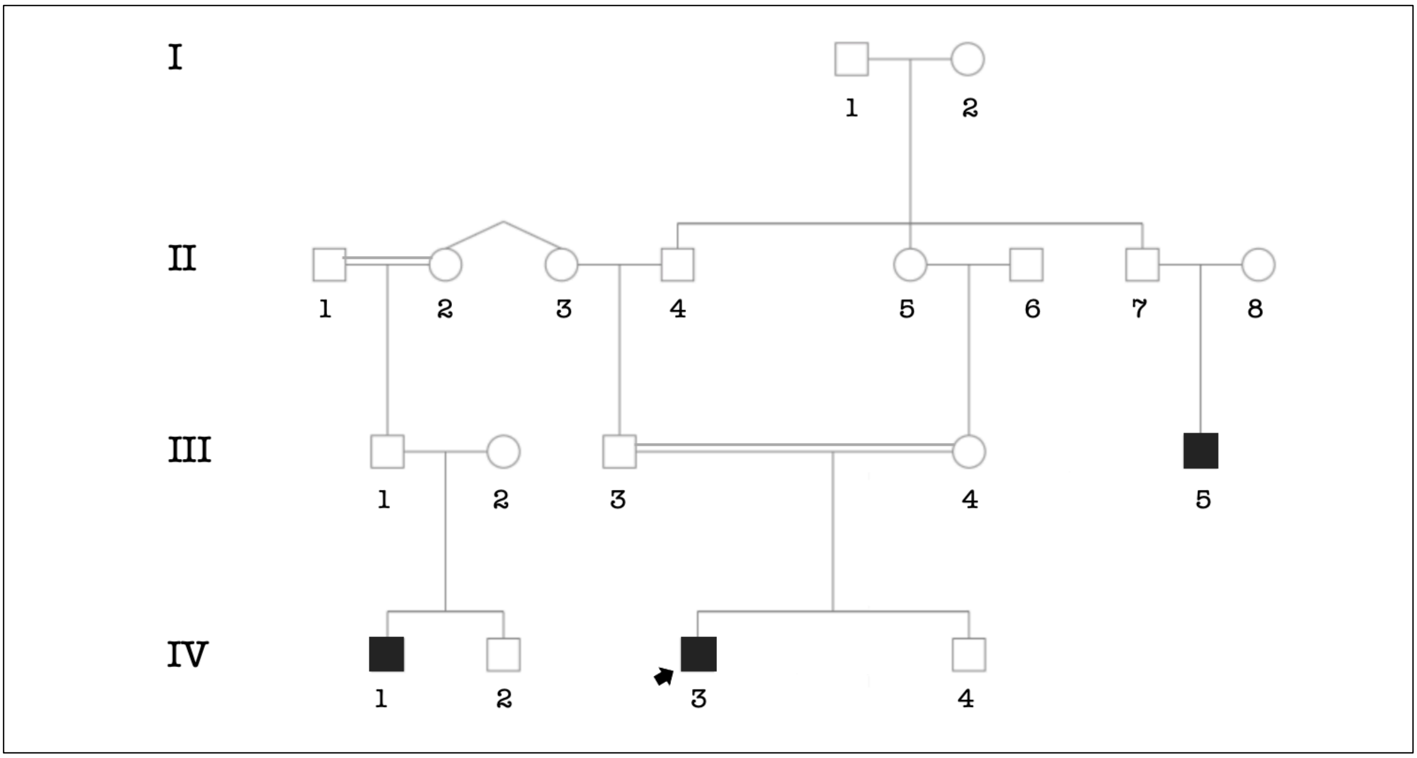

Family54. Pedigree showing three affected individuals segregating autosomal recessive non-syndromic hearing impairment

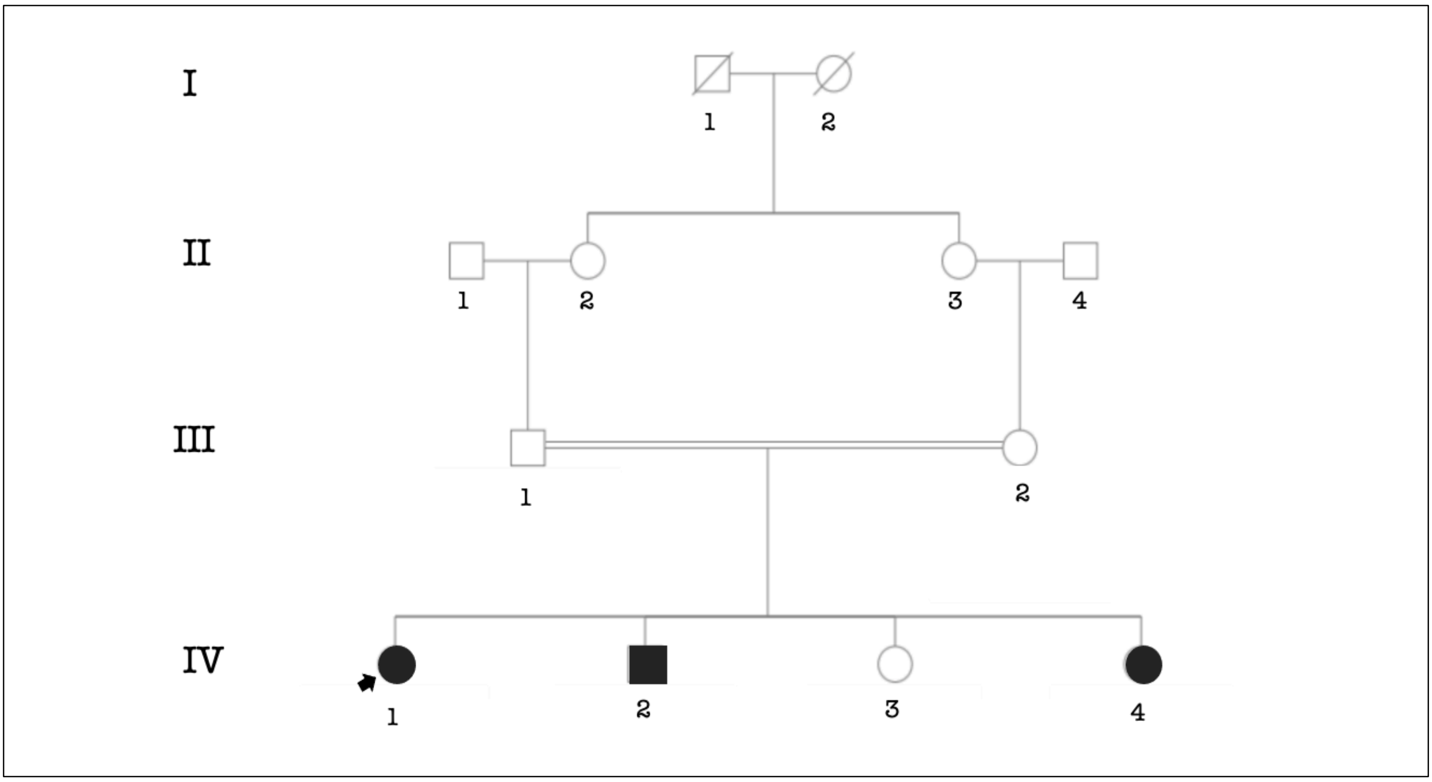

Family55. Pedigree showing three affected individuals from a consanguineous marriage segregating autosomal recessive non-syndromic hearing impairment

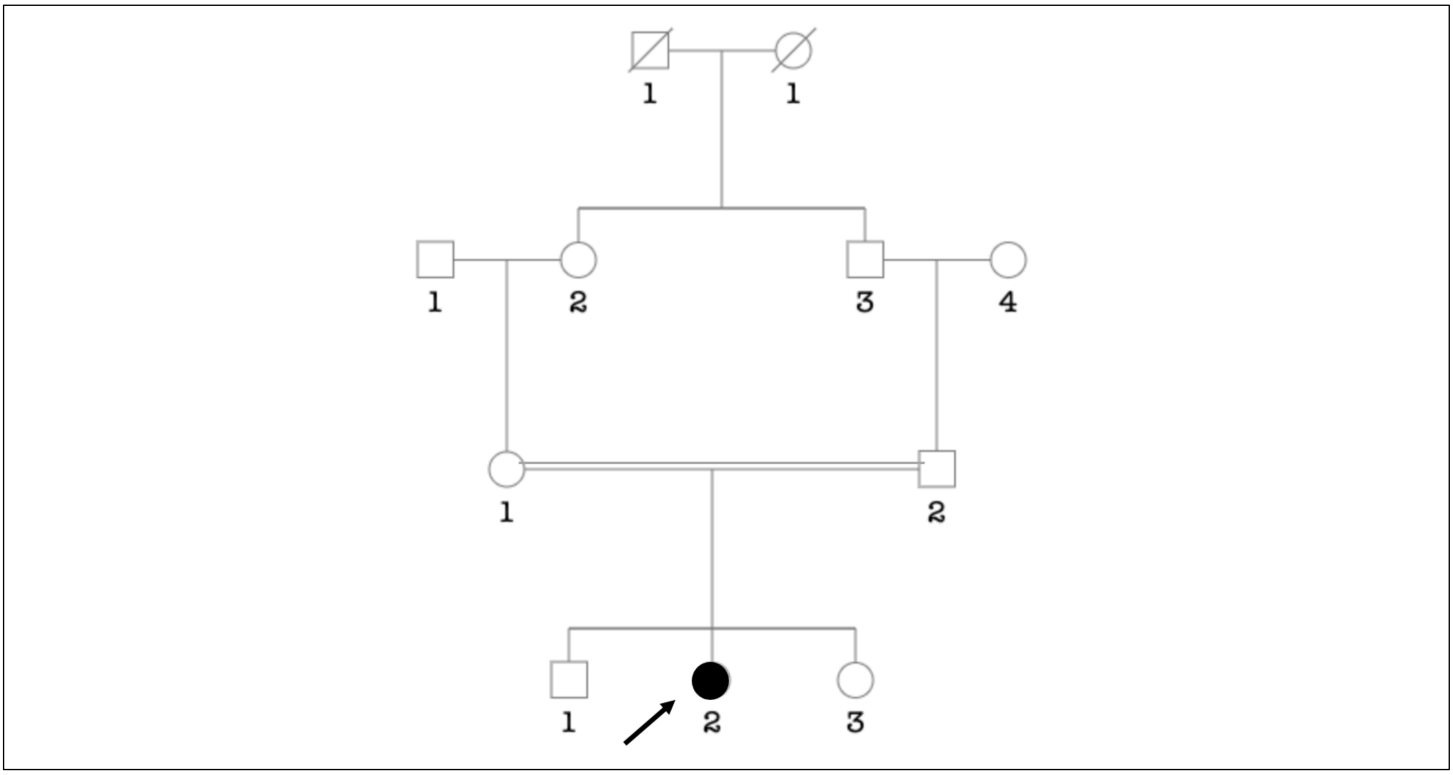

Family56. Pedigree showing a proband indicates by an arrow with non-syndromic hearing impairment

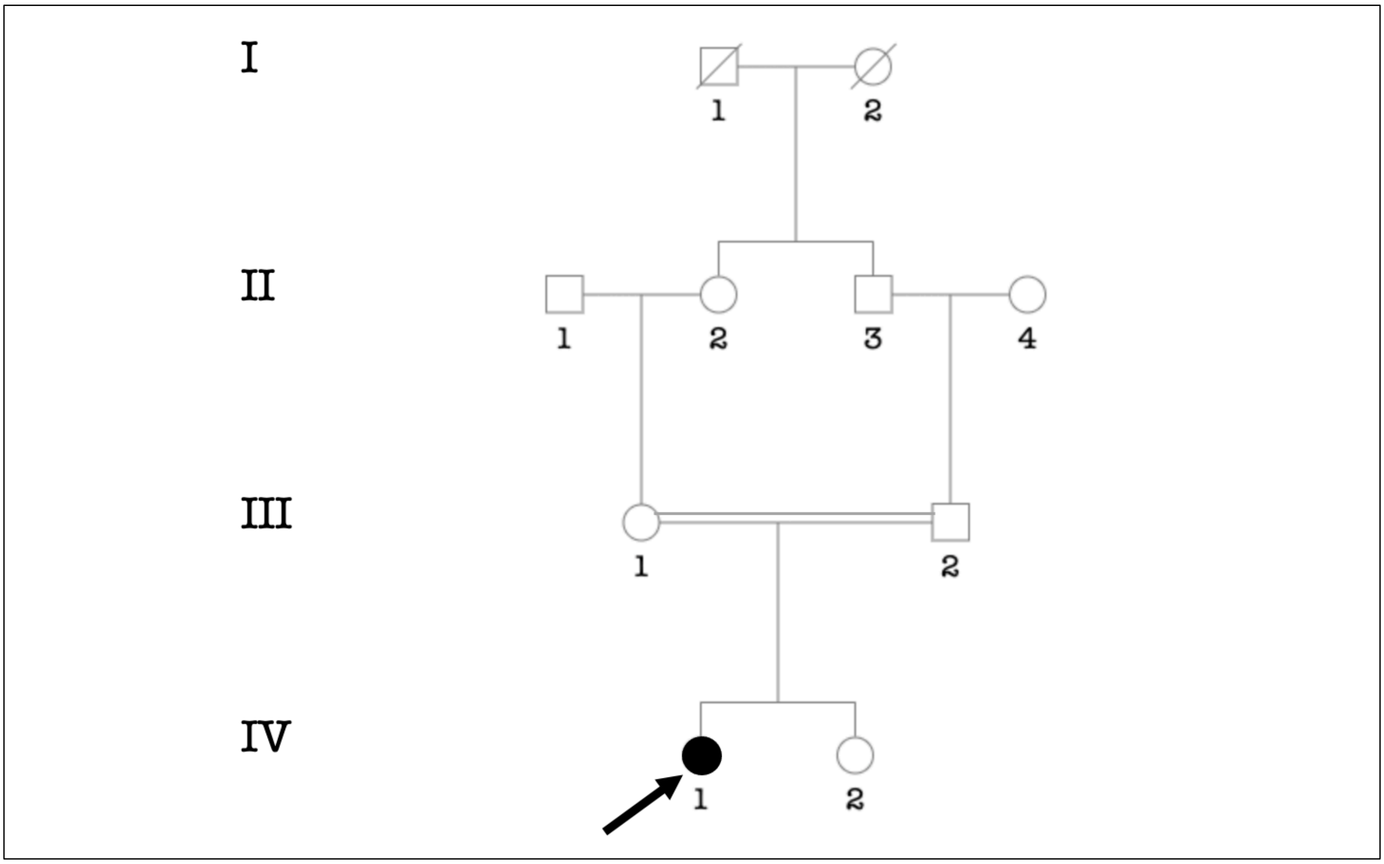

Family57. Pedigree showing a proband indicates by an arrow with non-syndromic hearing impairment

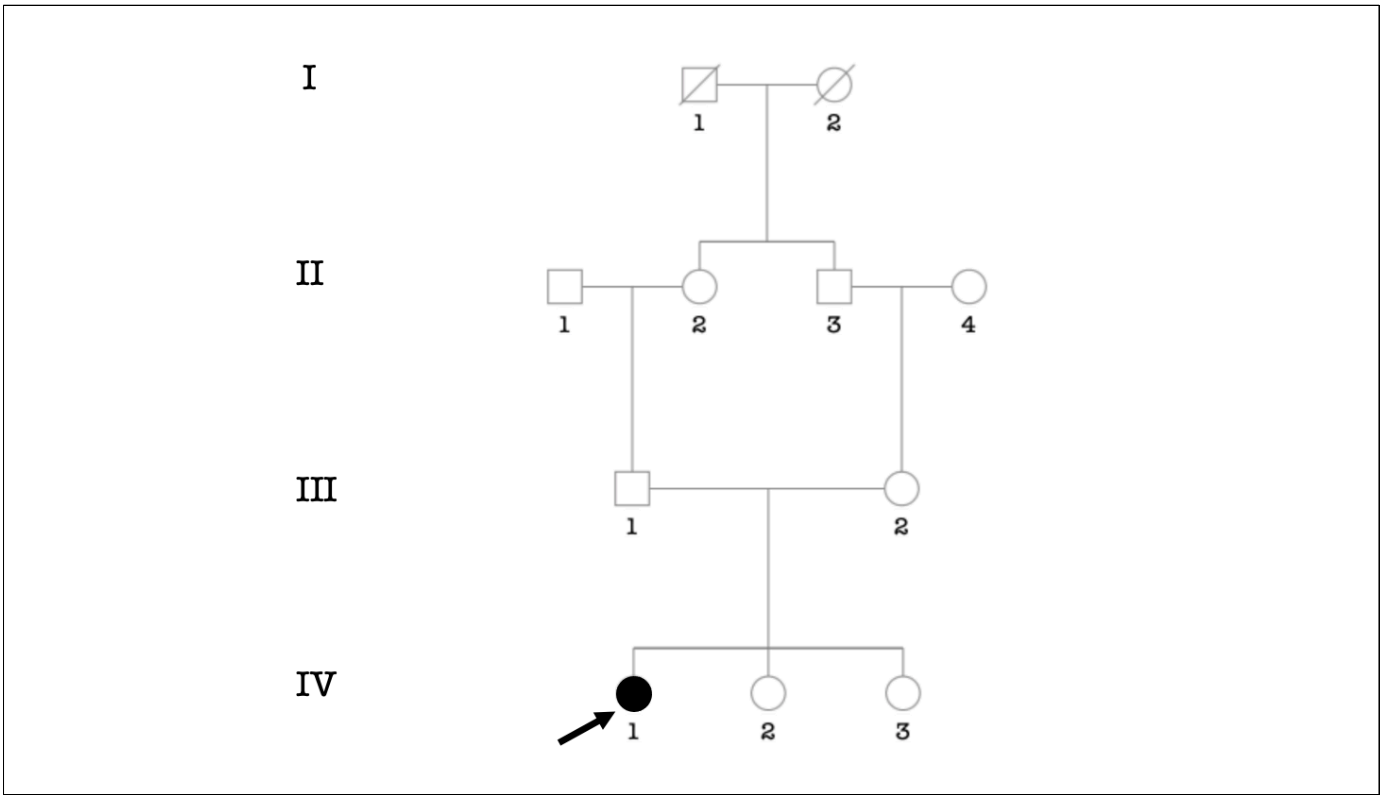

Family58. Pedigree showing a proband indicates by an arrow with non-syndromic hearing impairment

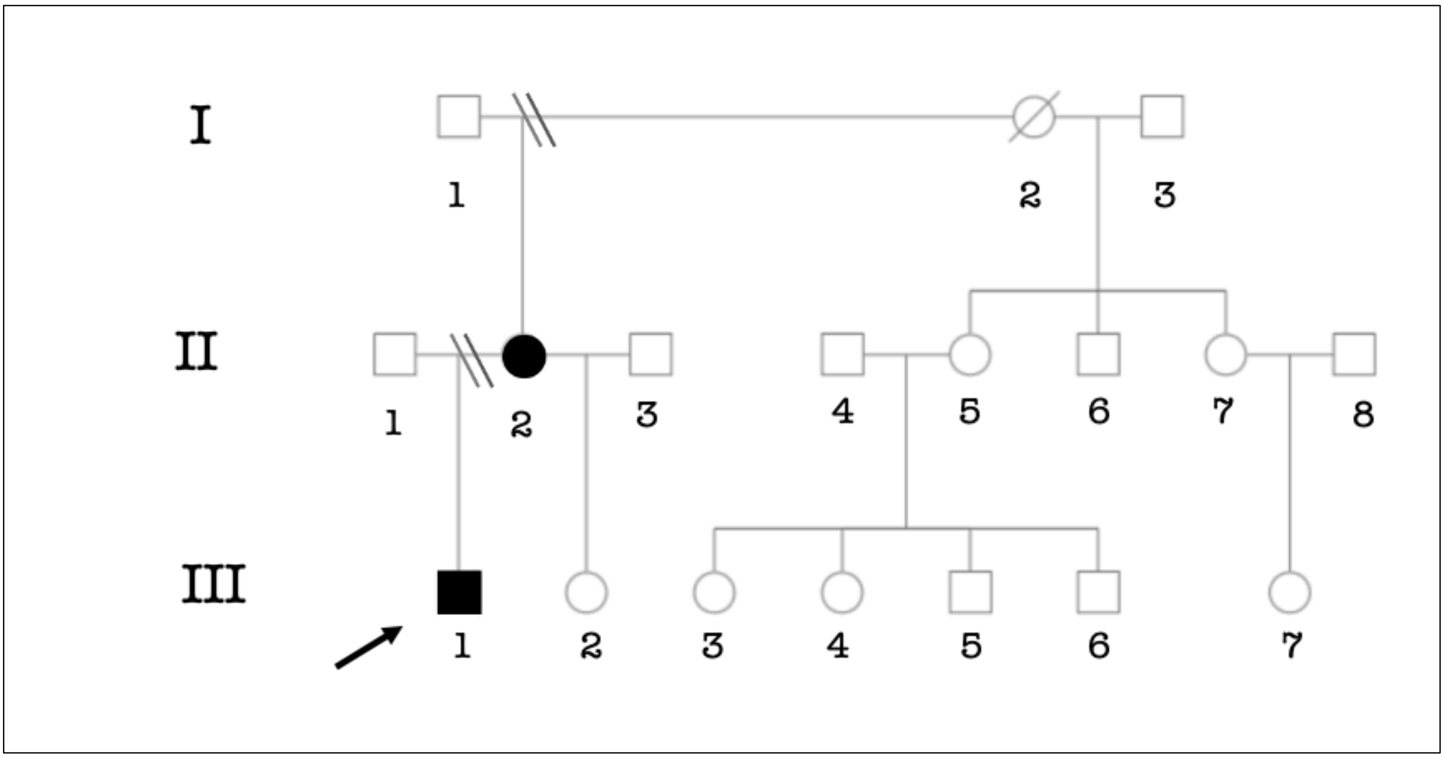

Family59. Pedigree showing two affected individuals segregating autosomal dominant non-syndromic hearing impairment

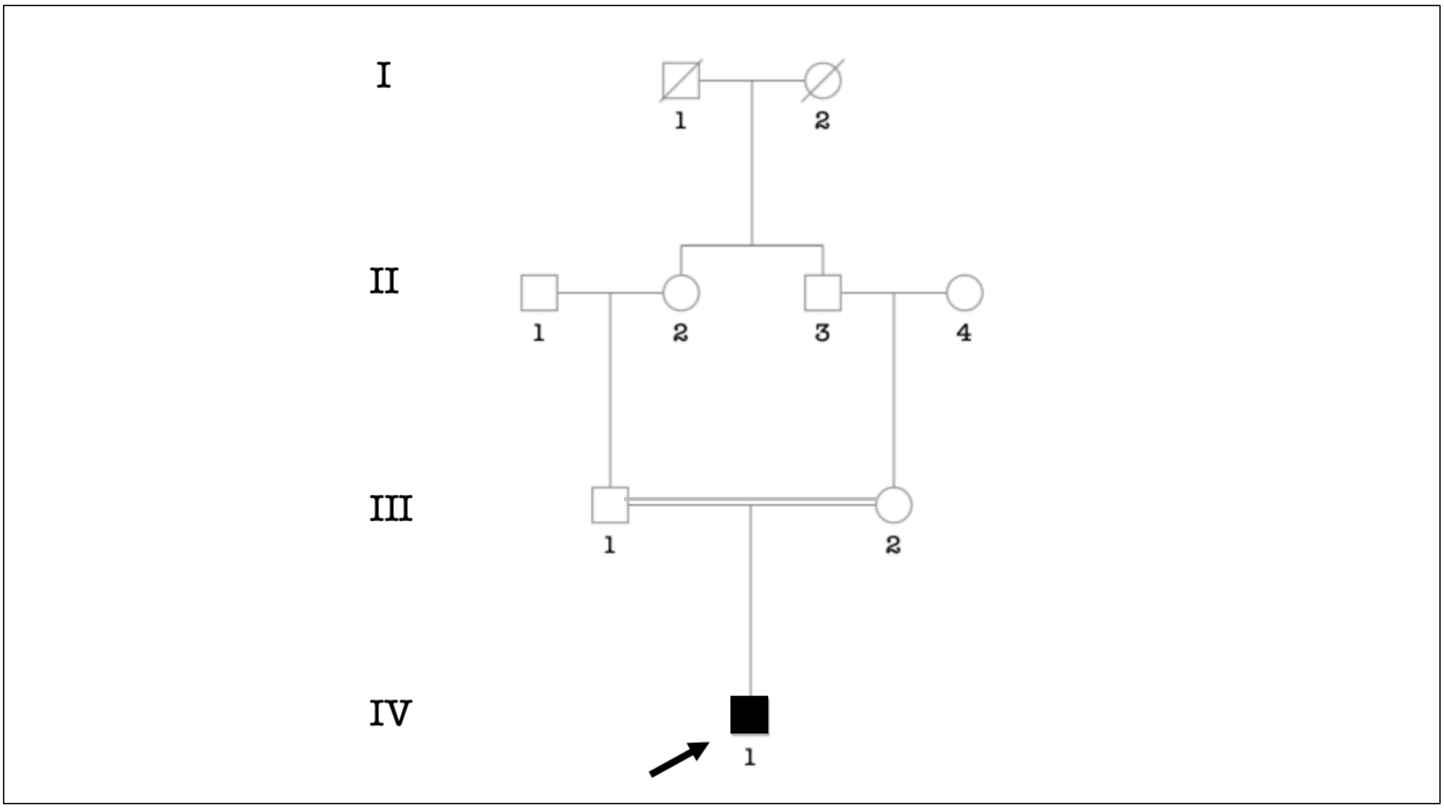

Family60. Pedigree showing a proband indicates by an arrow with non-syndromic hearing impairment

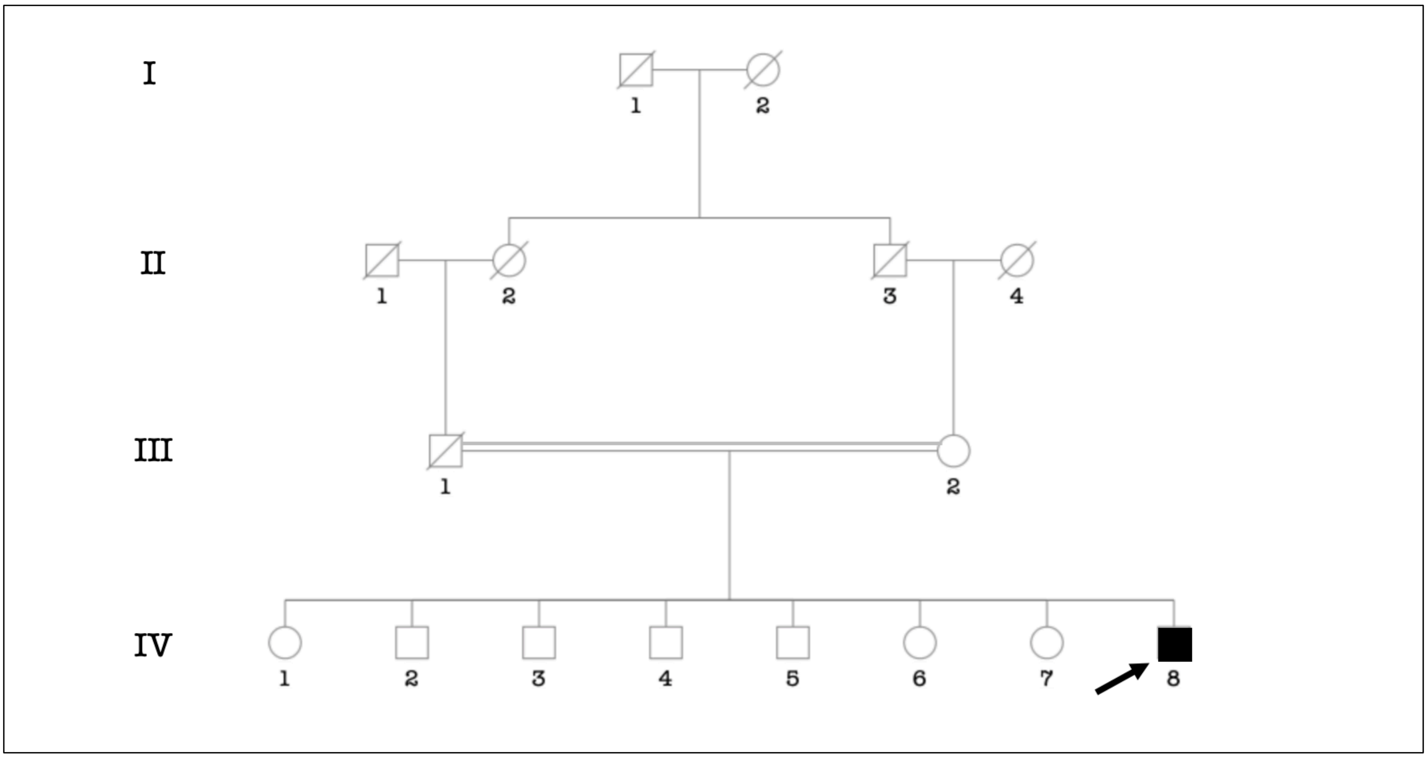

Family61. Pedigree showing a proband indicates by an arrow with non-syndromic hearing impairment

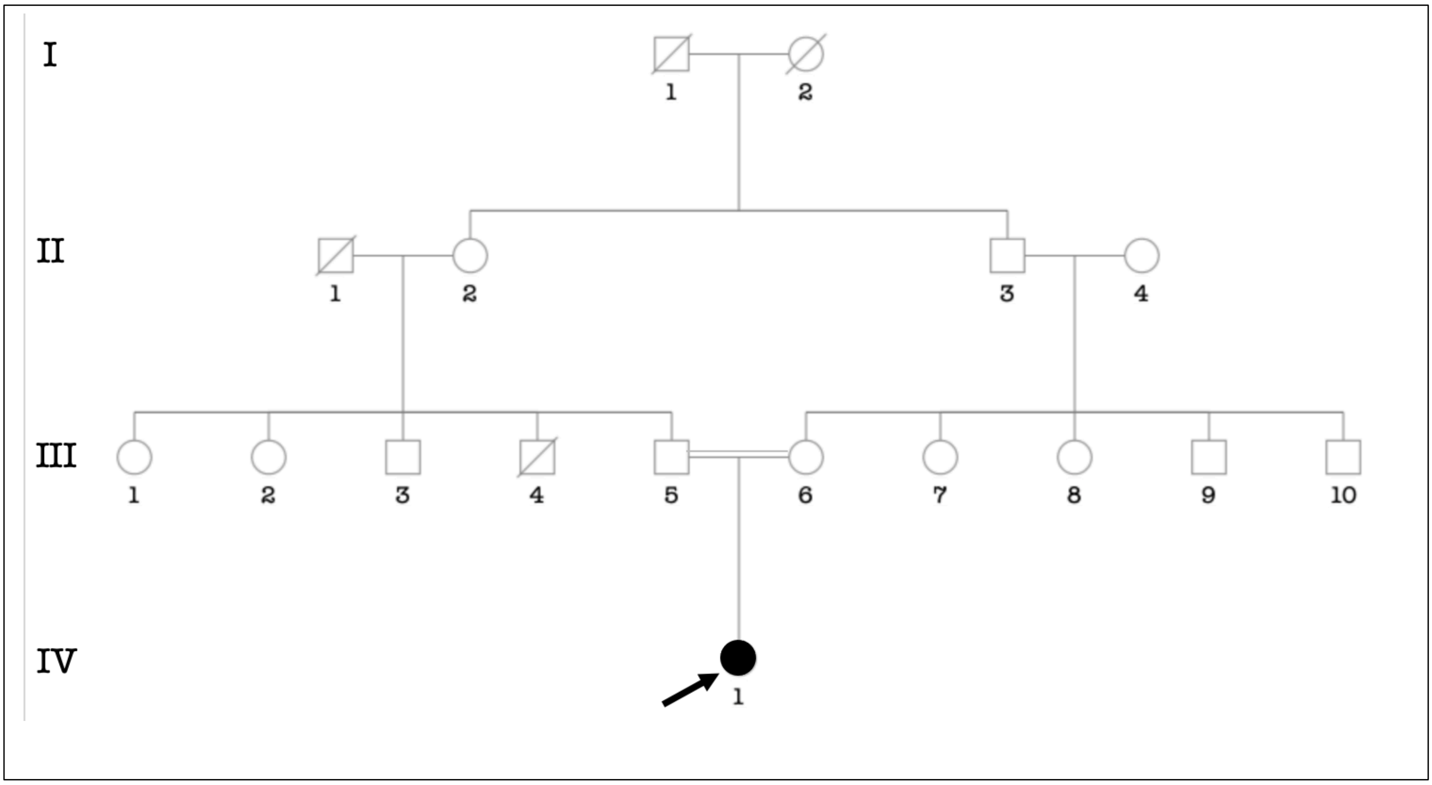

Family62. Pedigree showing a proband indicates by an arrow from a consanguineous marriage with non-syndromic hearing impairment

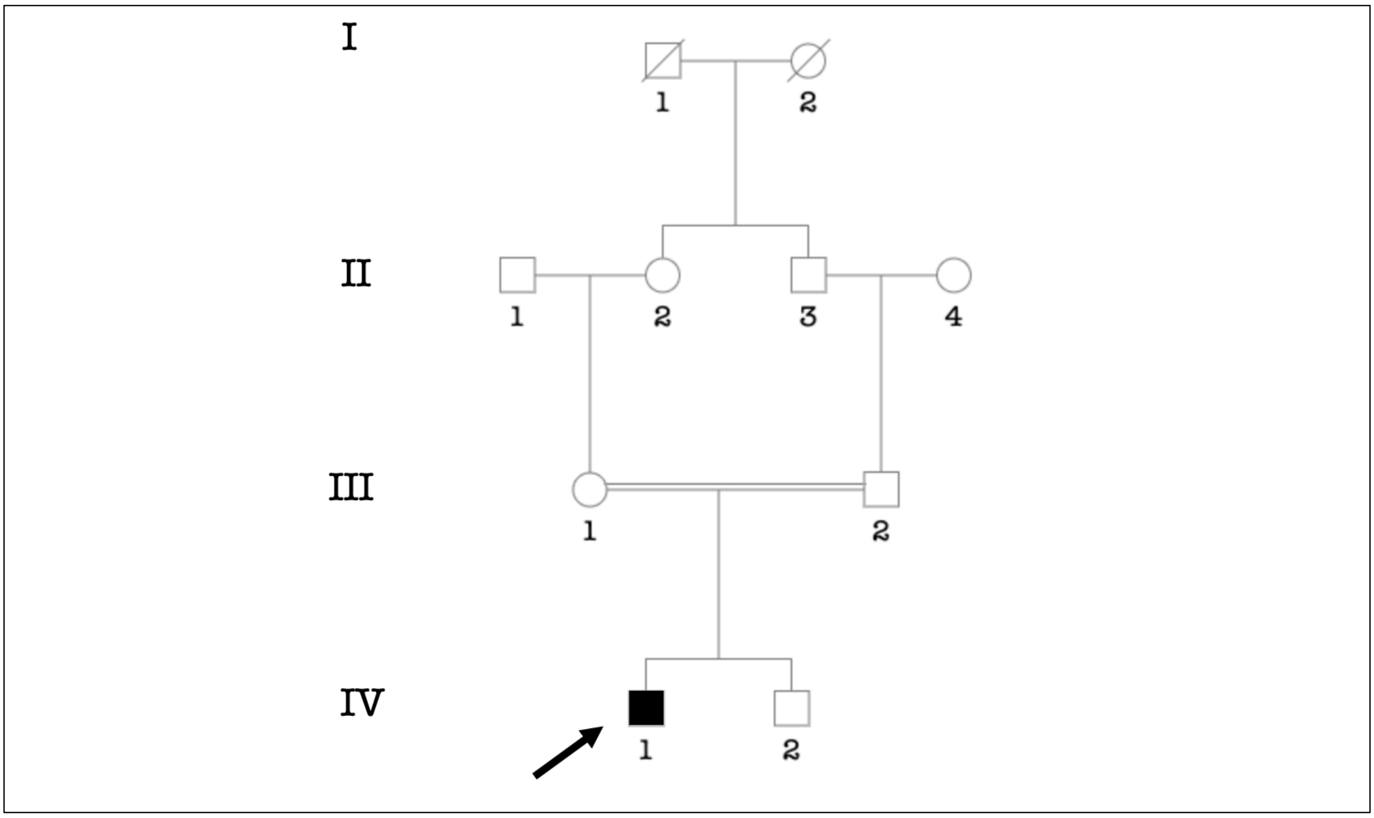

Family63. Pedigree showing a proband indicates by an arrow with non-syndromic hearing impairment

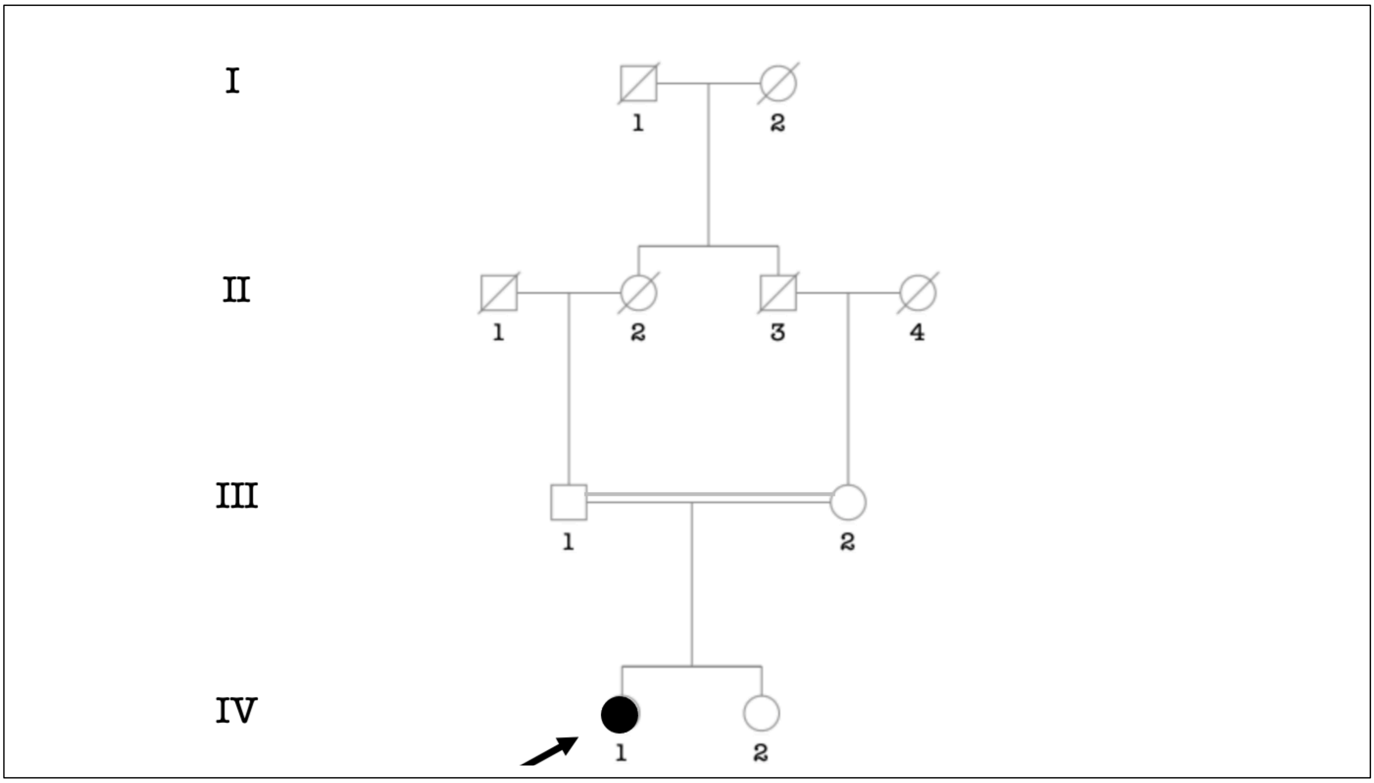

Family64. Pedigree showing a proband indicates by an arrow with non-syndromic hearing impairment

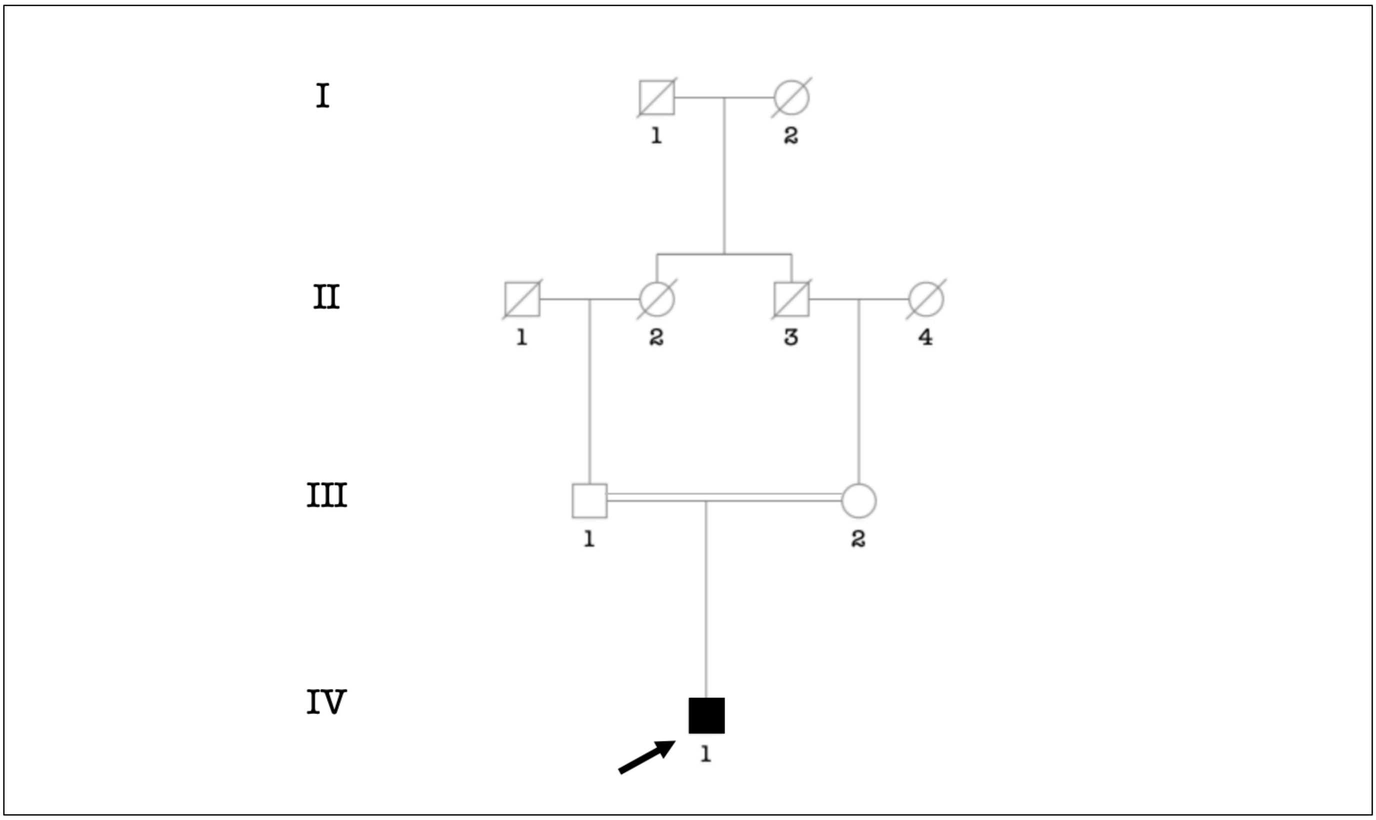

Family65. Pedigree showing a proband indicates by an arrow with non-syndromic hearing impairment

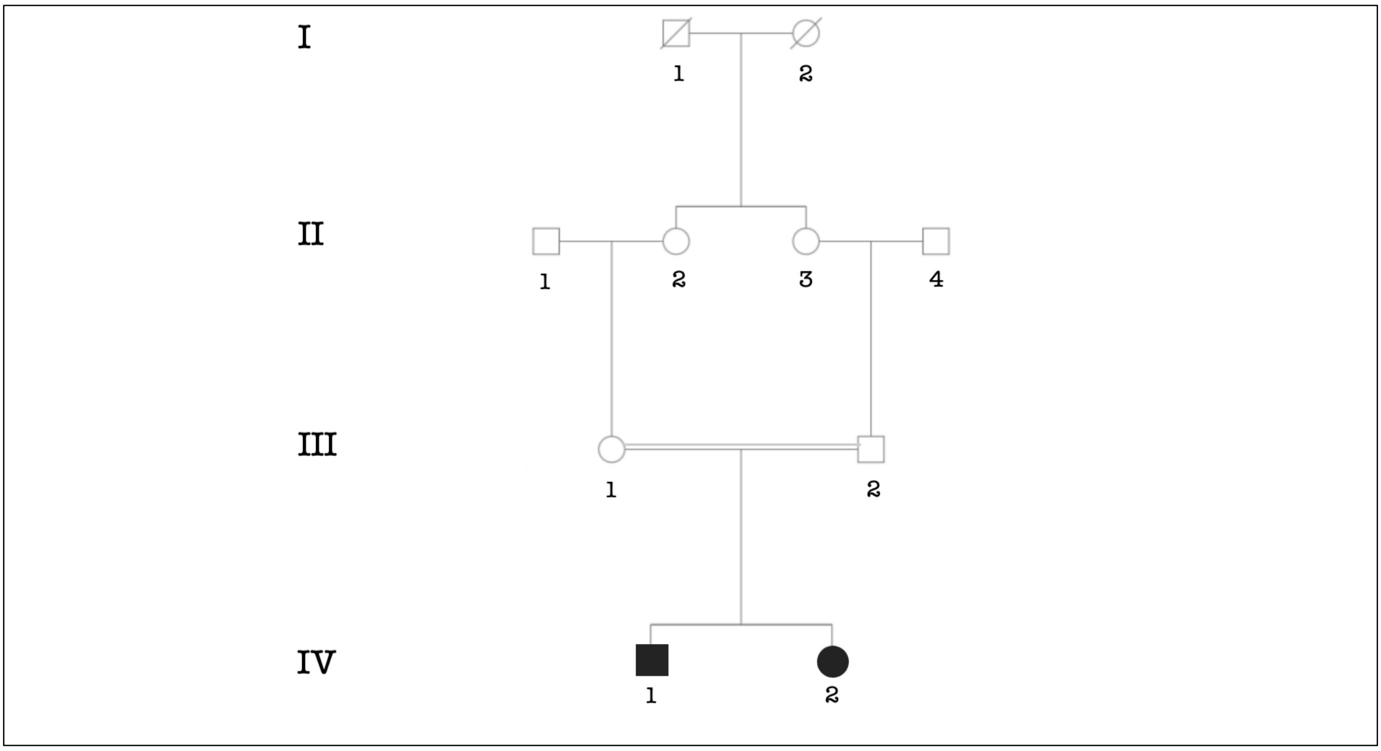

Family66. Showing two affected individuals from a consanguineous marriage segregating autosomal recessive non-syndromic hearing impairment.

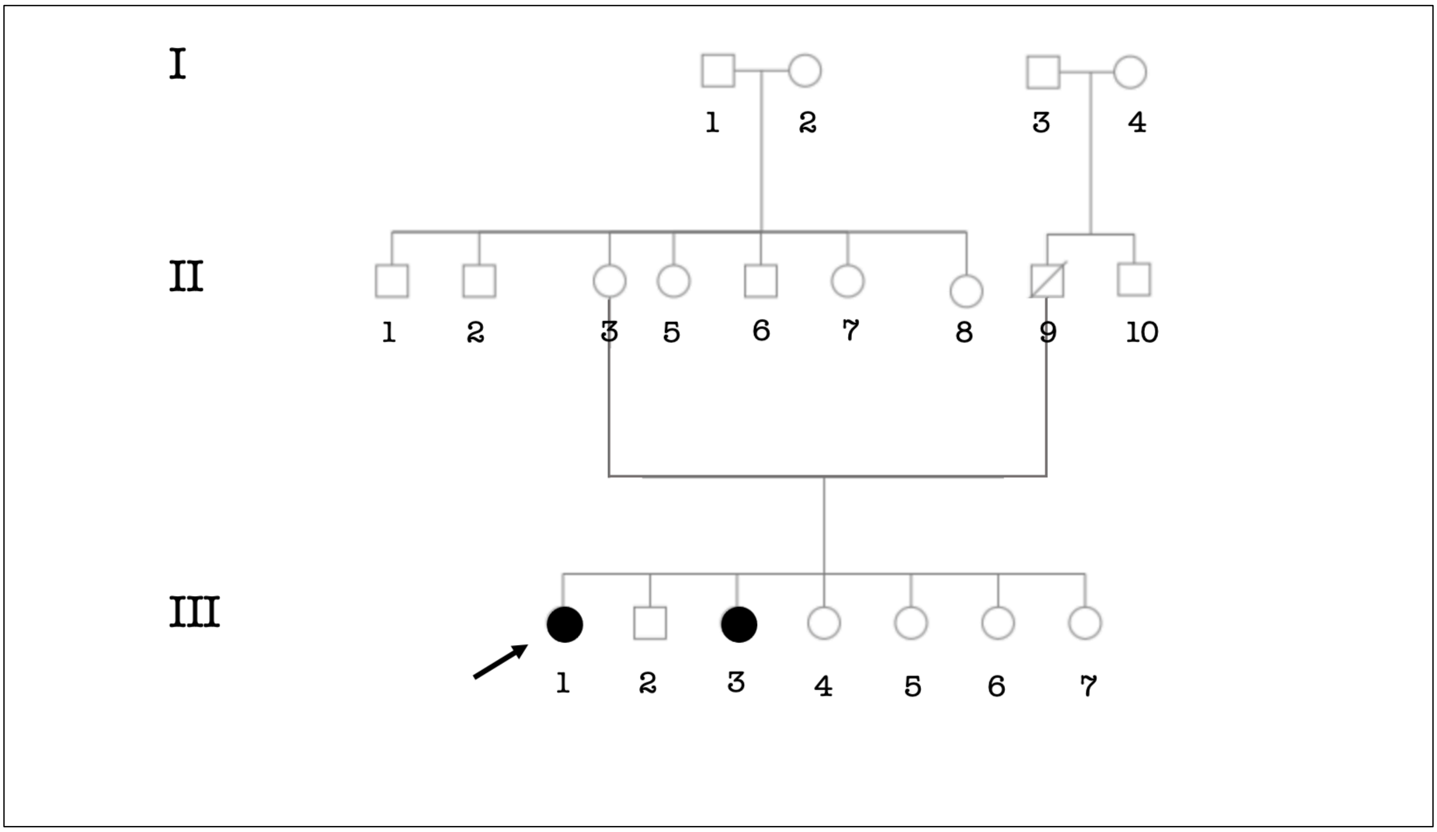

Family67. Showing two affected individuals from a non-consanguineous marriage segregating an autosomal recessive non-specific syndrome

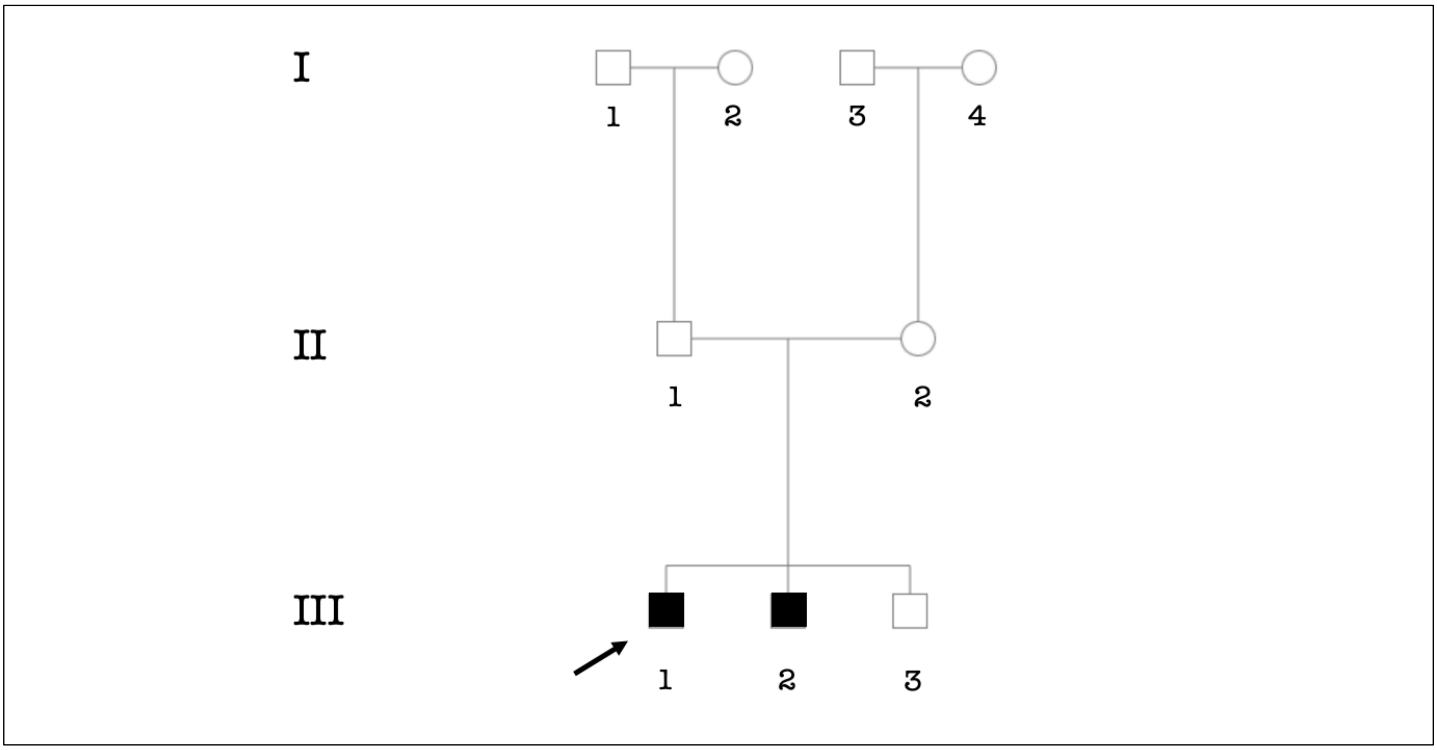

Family68. Showing two affected individuals from a non-consanguineous marriage segregating likely autosomal recessive non-syndromic hearing impairment.

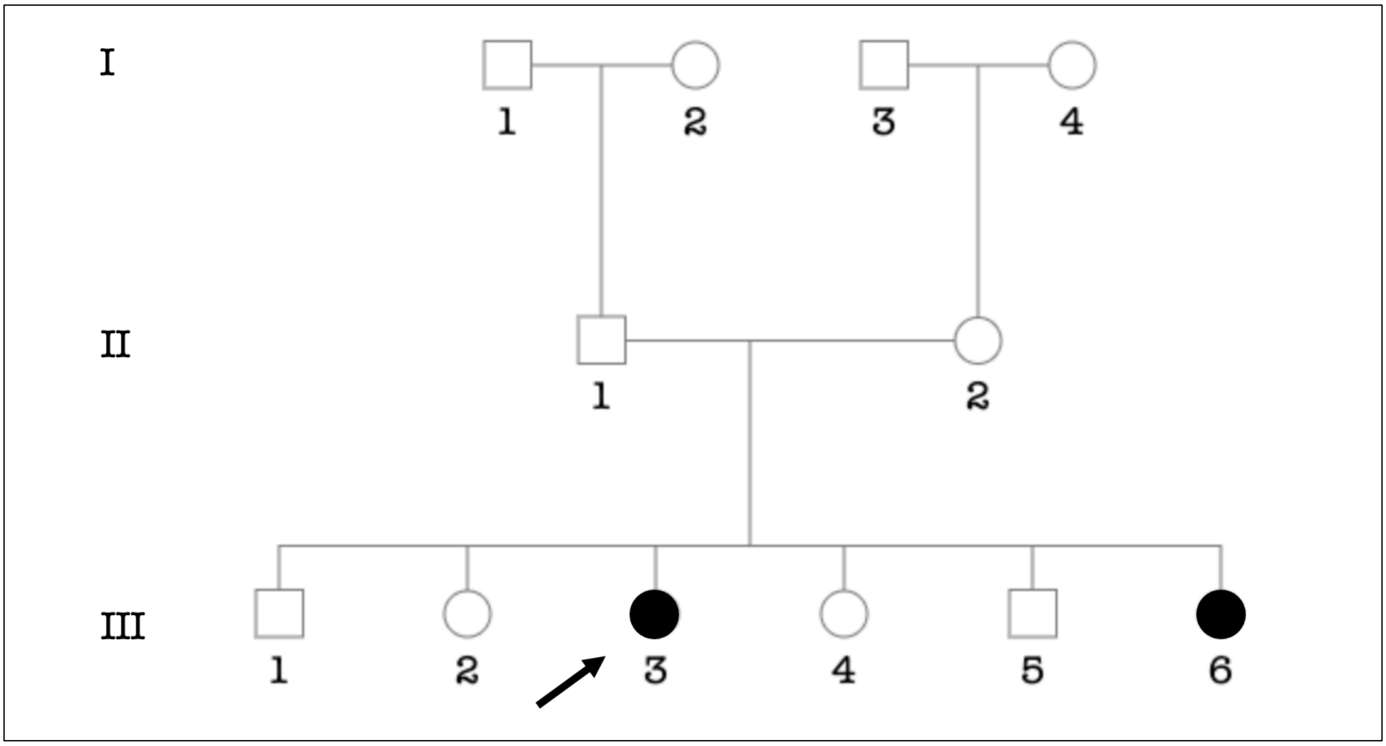

Family69. Pedigree showing two affected girls from a non-consanguineous marriage segregating autosomal recessive non-syndromic hearing impairment

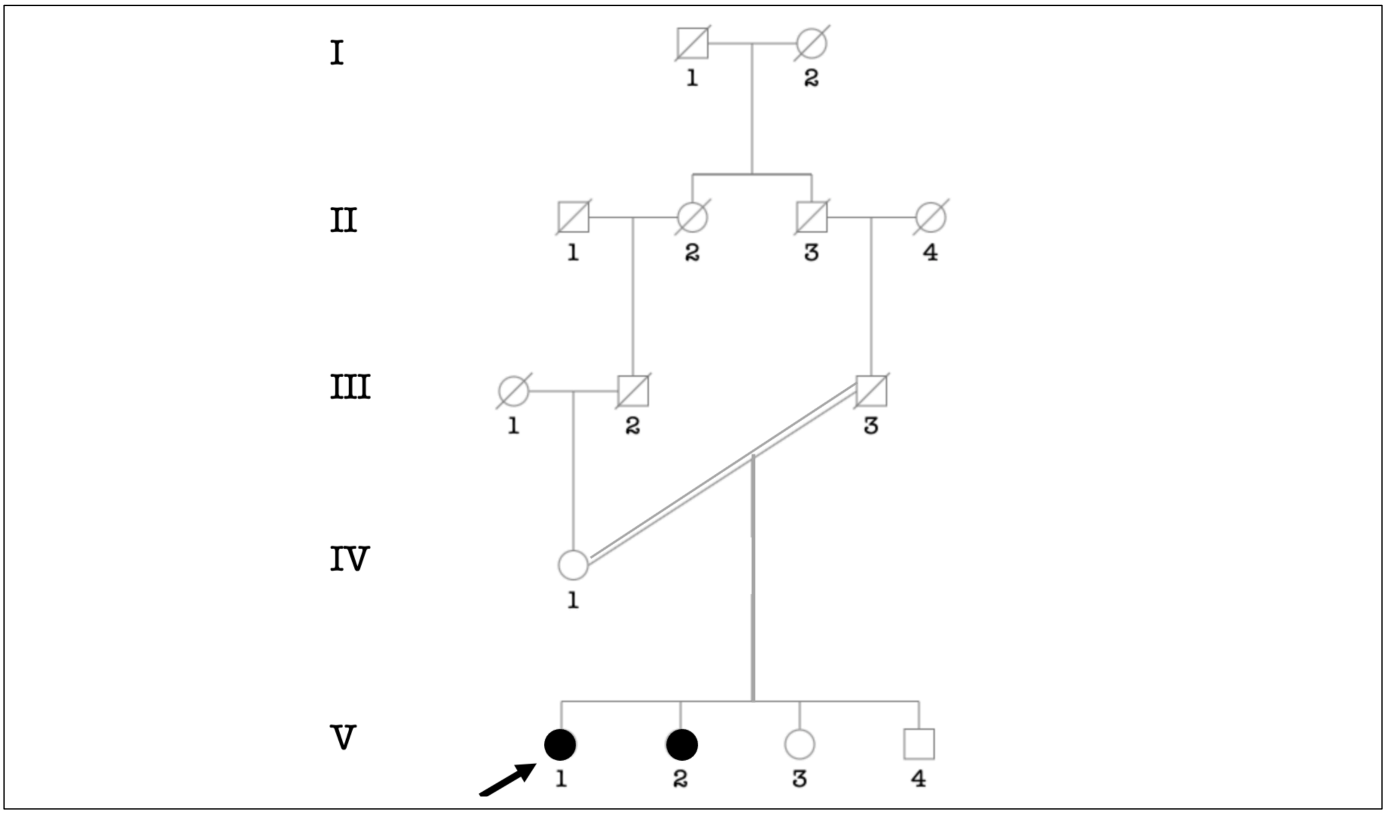

Family70. Pedigree showing two affected girls from a consanguineous marriage segregating autosomal recessive non-syndromic hearing impairment

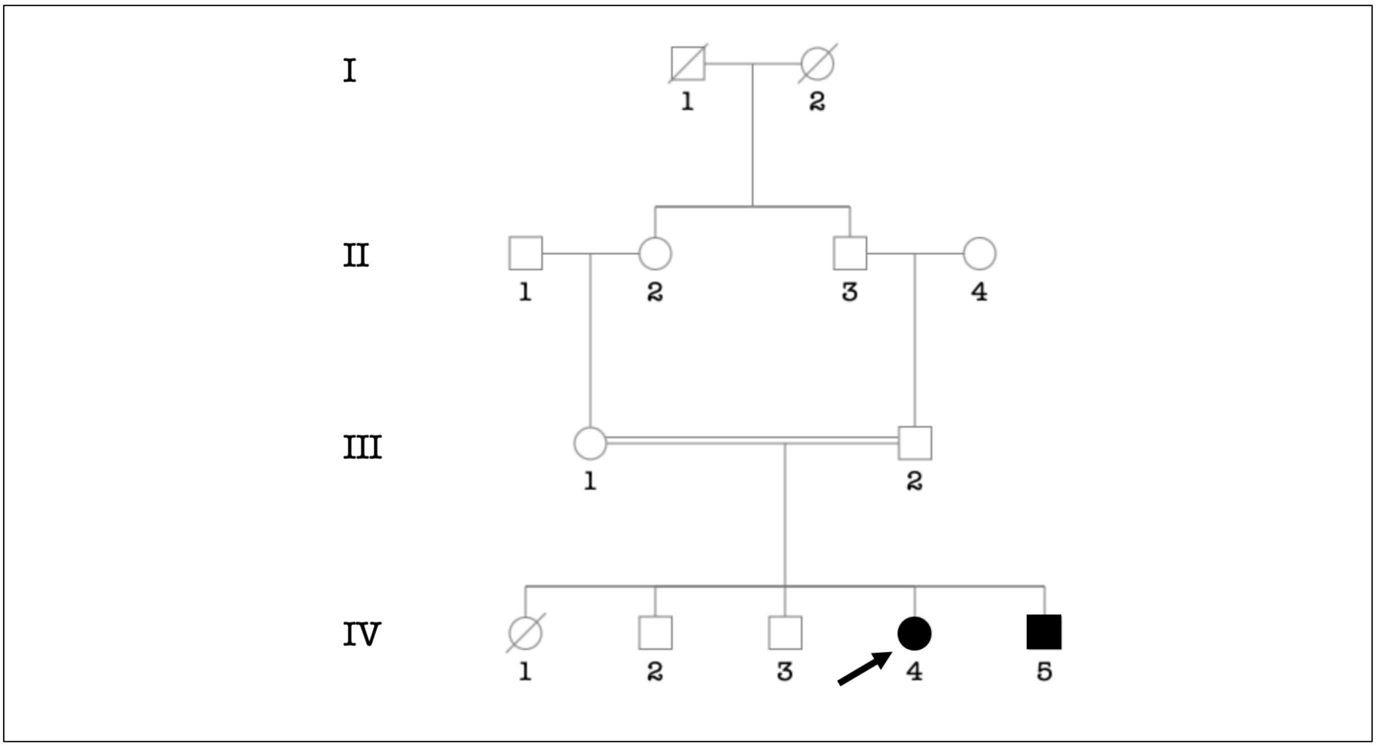

Family71. Pedigree showing two affected individuals from a consanguineous marriage segregating autosomal recessive non-syndromic hearing impairment

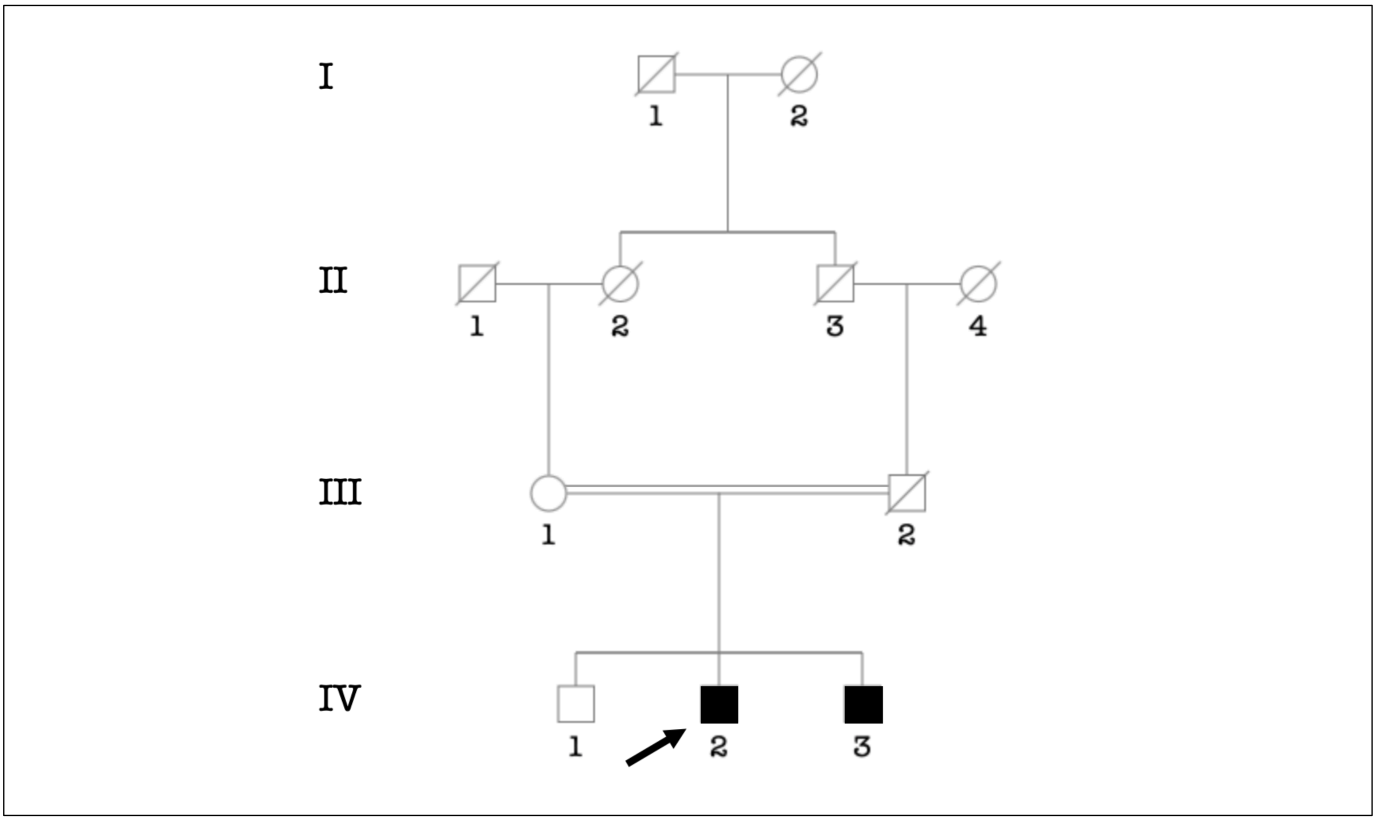

Family72. Pedigree showing two affected boys from a consanguineous marriage segregating autosomal recessive non-syndromic hearing impairment

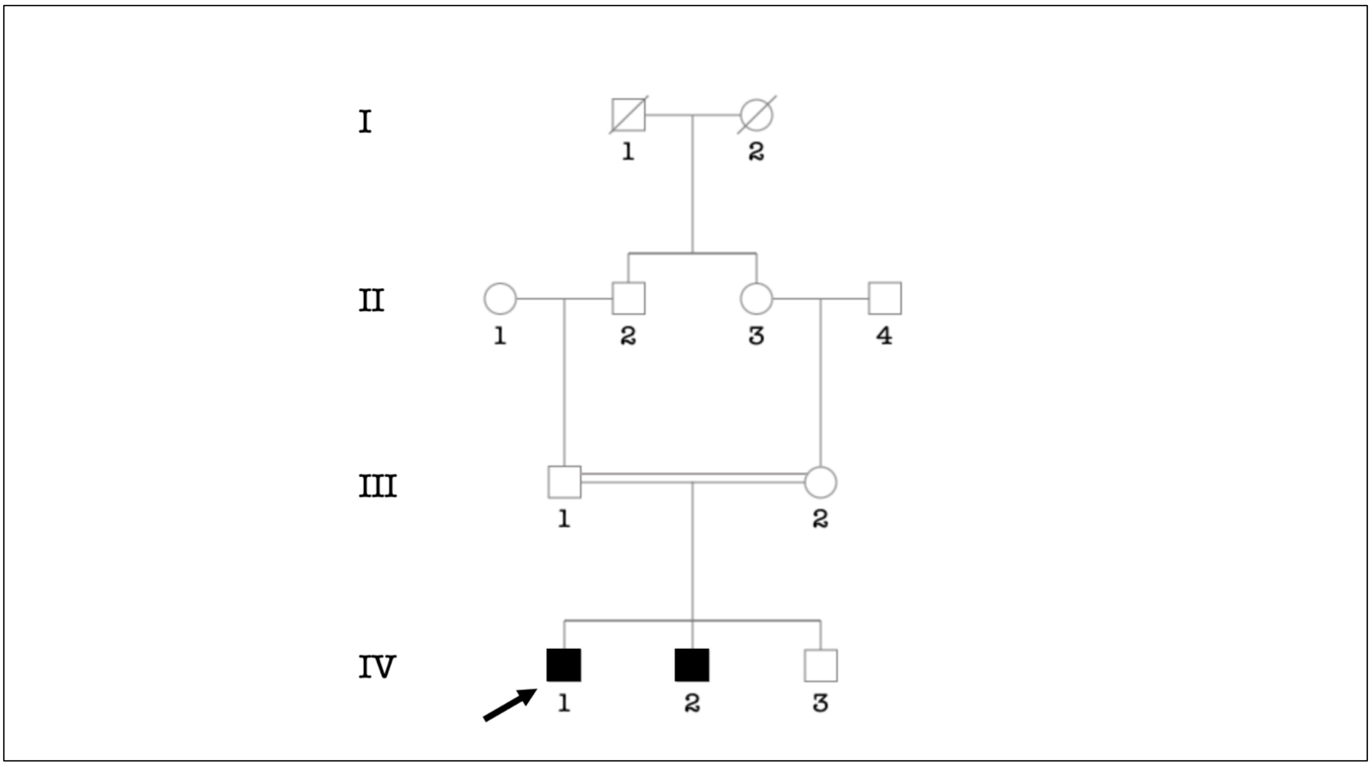

Family73. Pedigree showing two affected boys from a consanguineous marriage segregating autosomal recessive non-syndromic hearing impairment

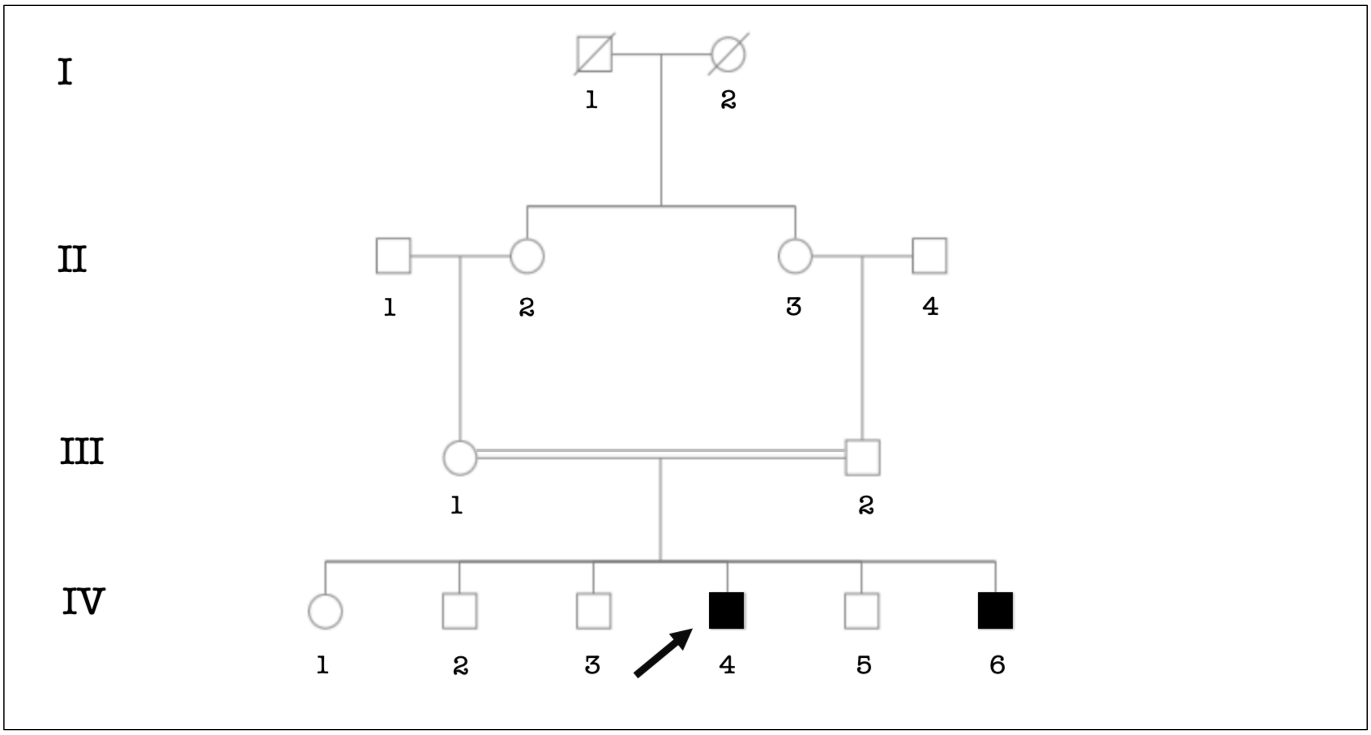

Family74. Pedigree showing two affected boys from a consanguineous marriage segregating autosomal recessive non-syndromic hearing impairment

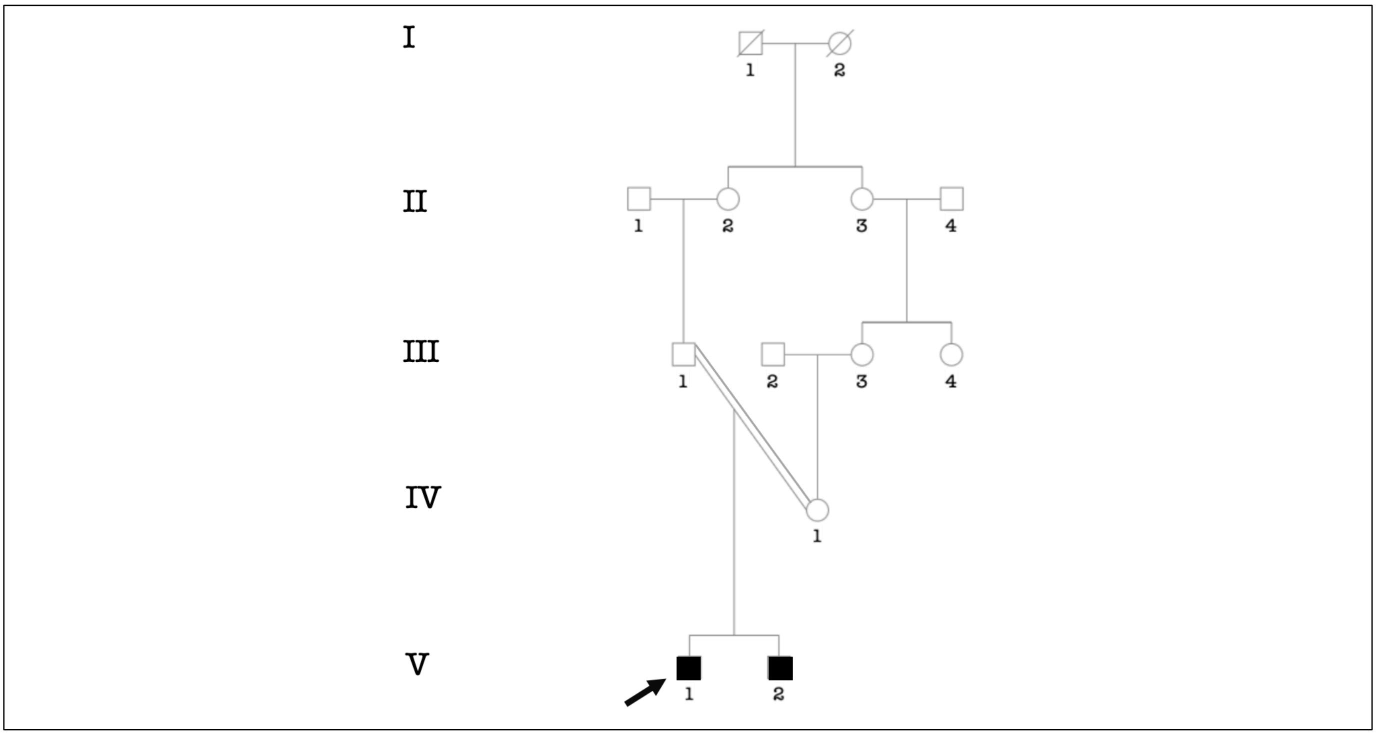

Family75. Pedigree showing two affected individuals from a consanguineous marriage segregating autosomal recessive non-syndromic hearing impairment.

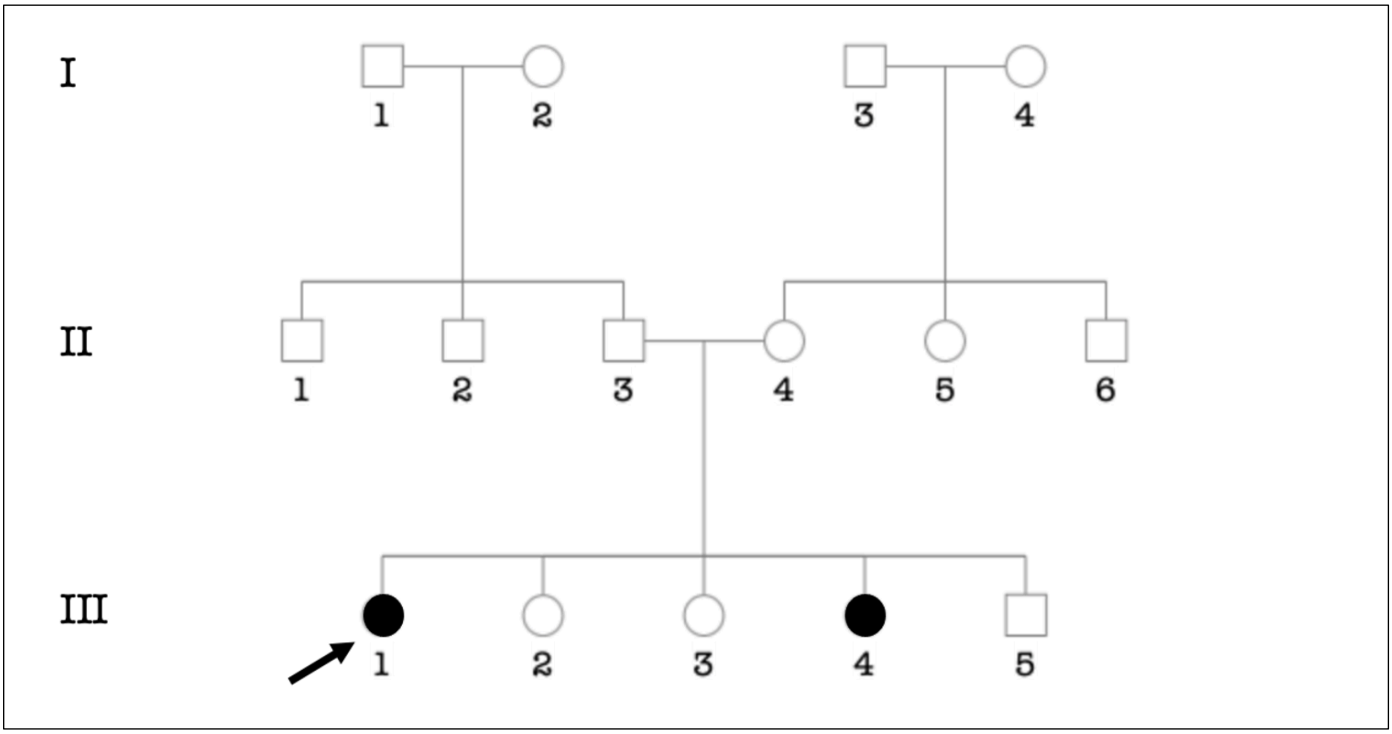

Family76. Pedigree showing two affected girls segregating autosomal recessive non-syndromic hearing impairment.

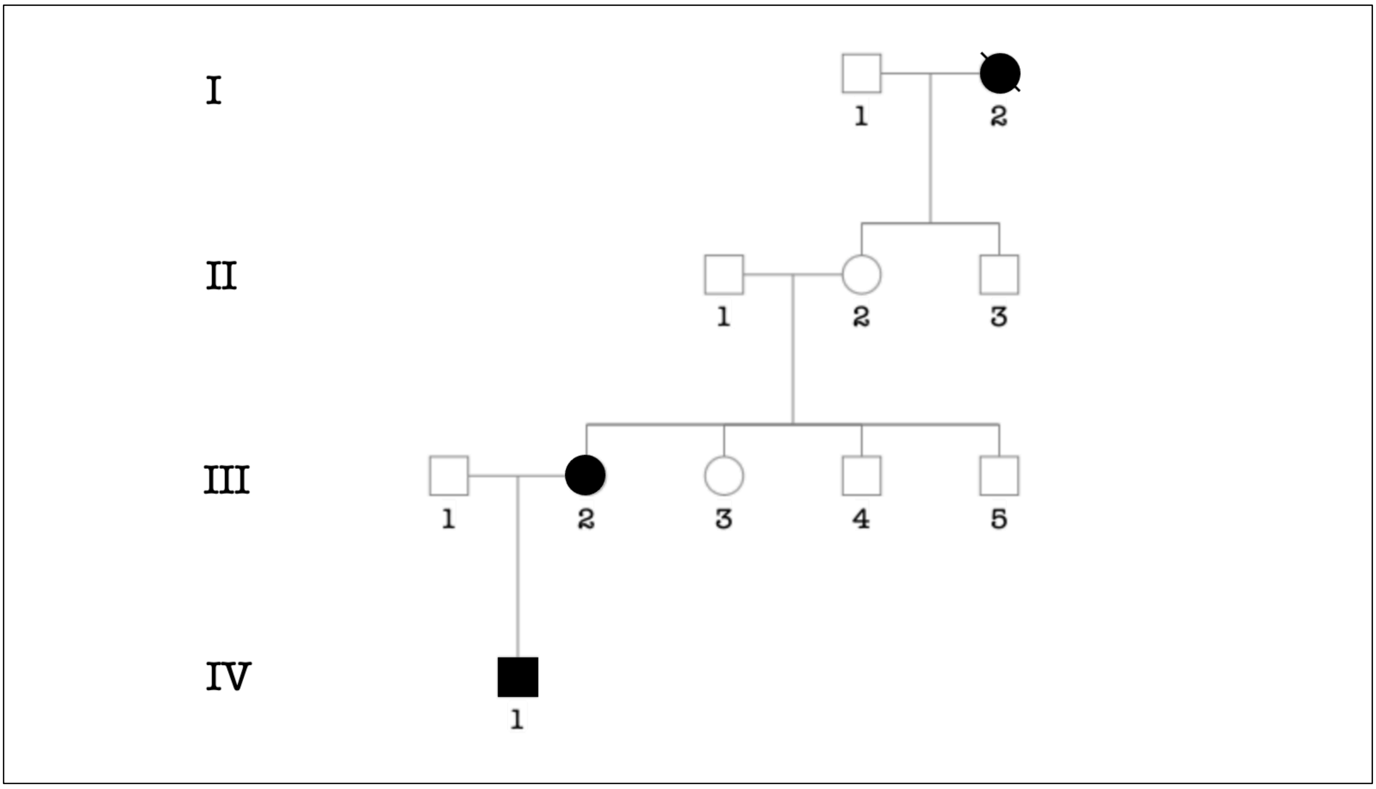

Family77. Pedigree showing three affected individuals segregating autosomal dominant non-syndromic hearing impairment

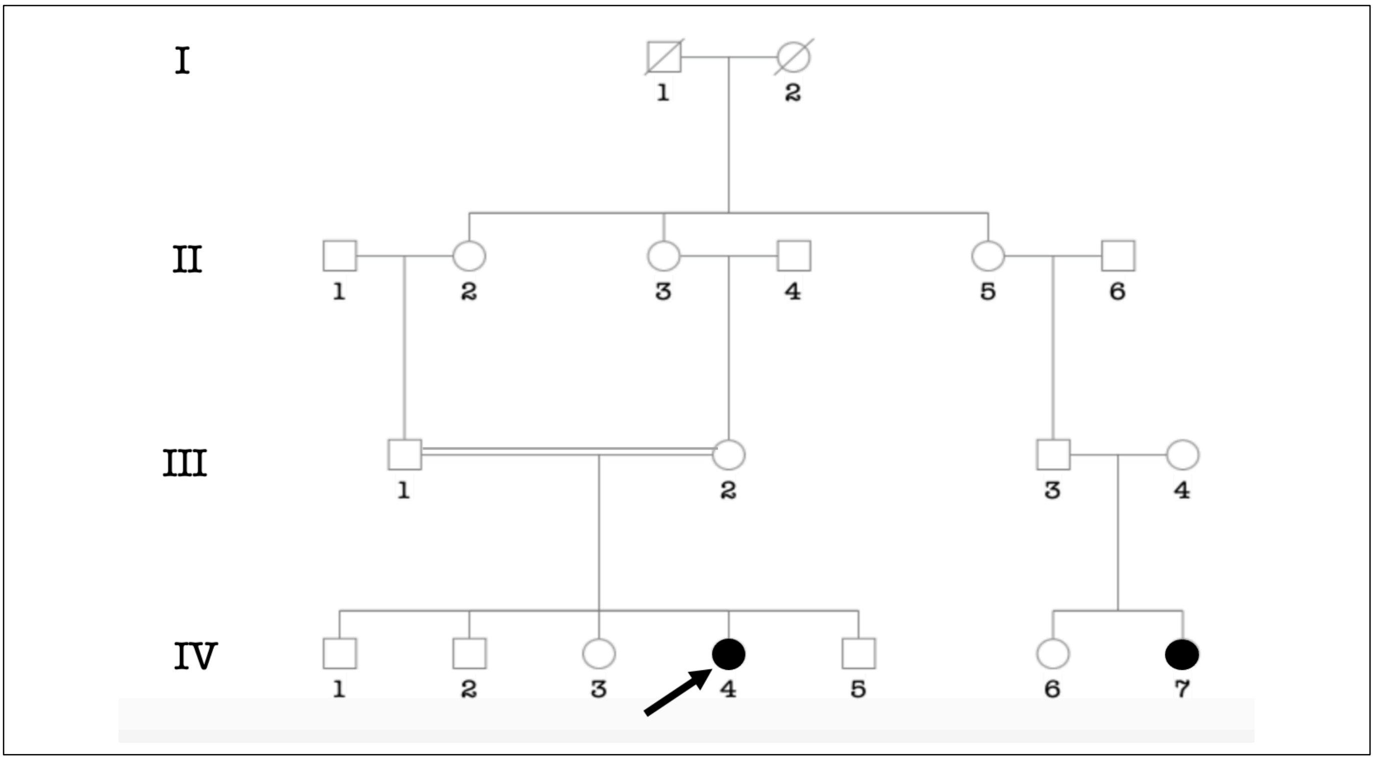

Family78. Pedigree showing two affected girls segregating autosomal recessive non-syndromic hearing impairment

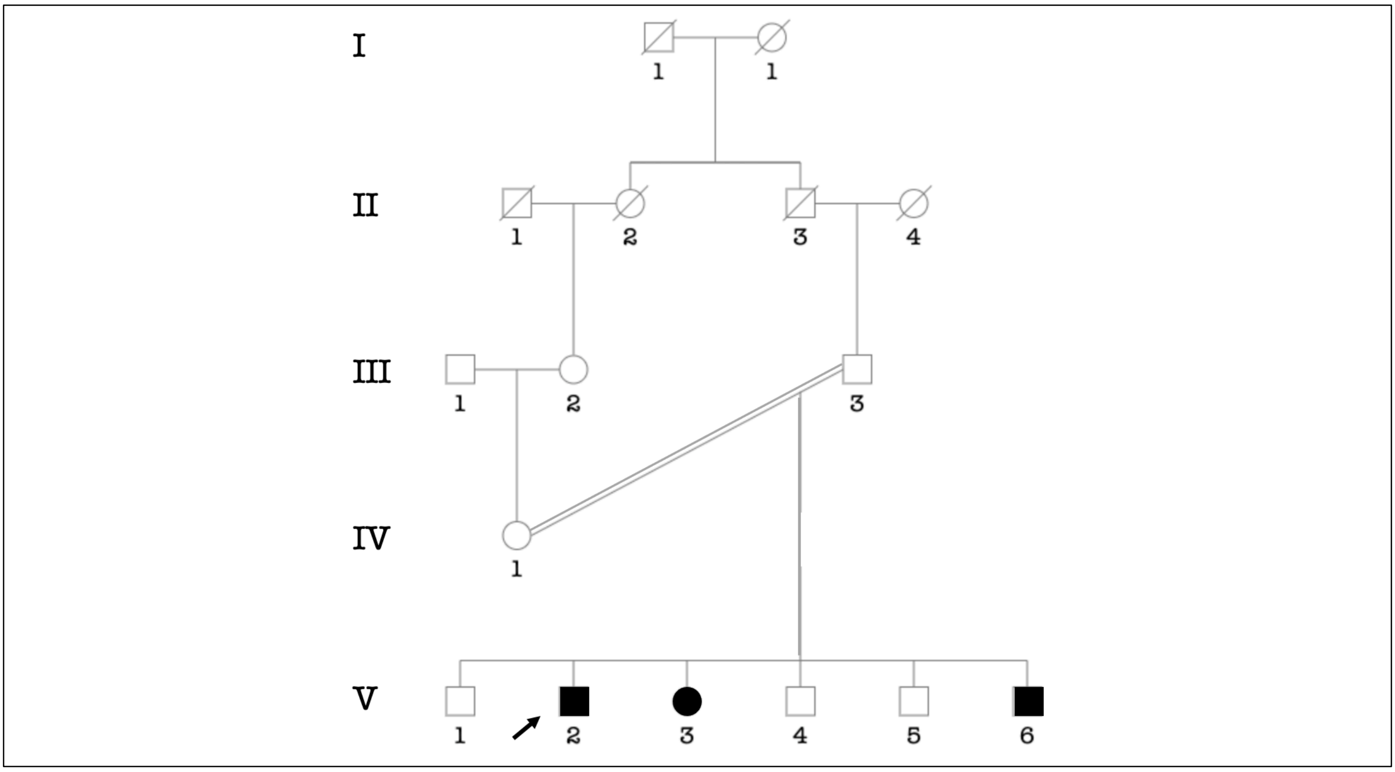

Family79. Pedigree showing three affected individuals from a consanguineous marriage segregating autosomal recessive non-syndromic hearing impairment

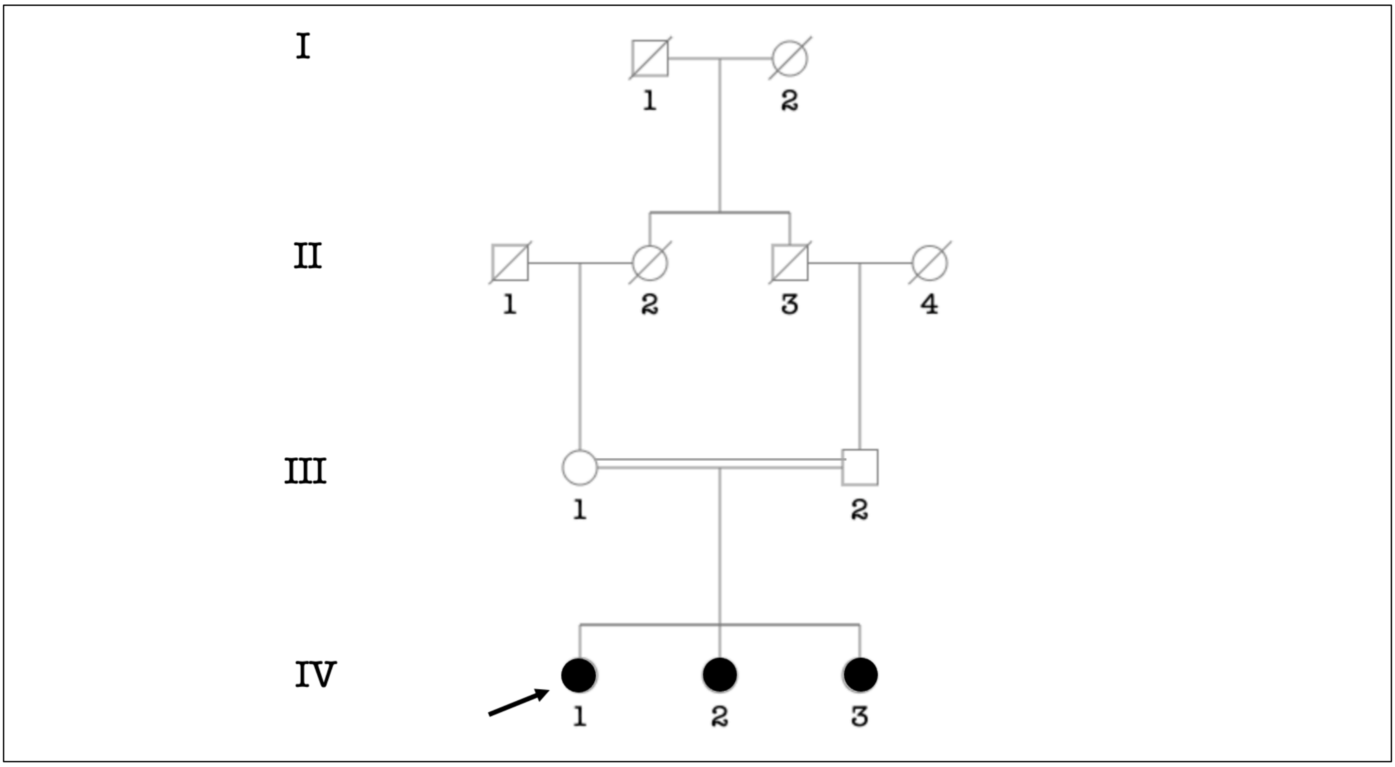

Family80. Pedigree showing three affected individuals from a consanguineous marriage segregating autosomal recessive non-syndromic hearing impairment

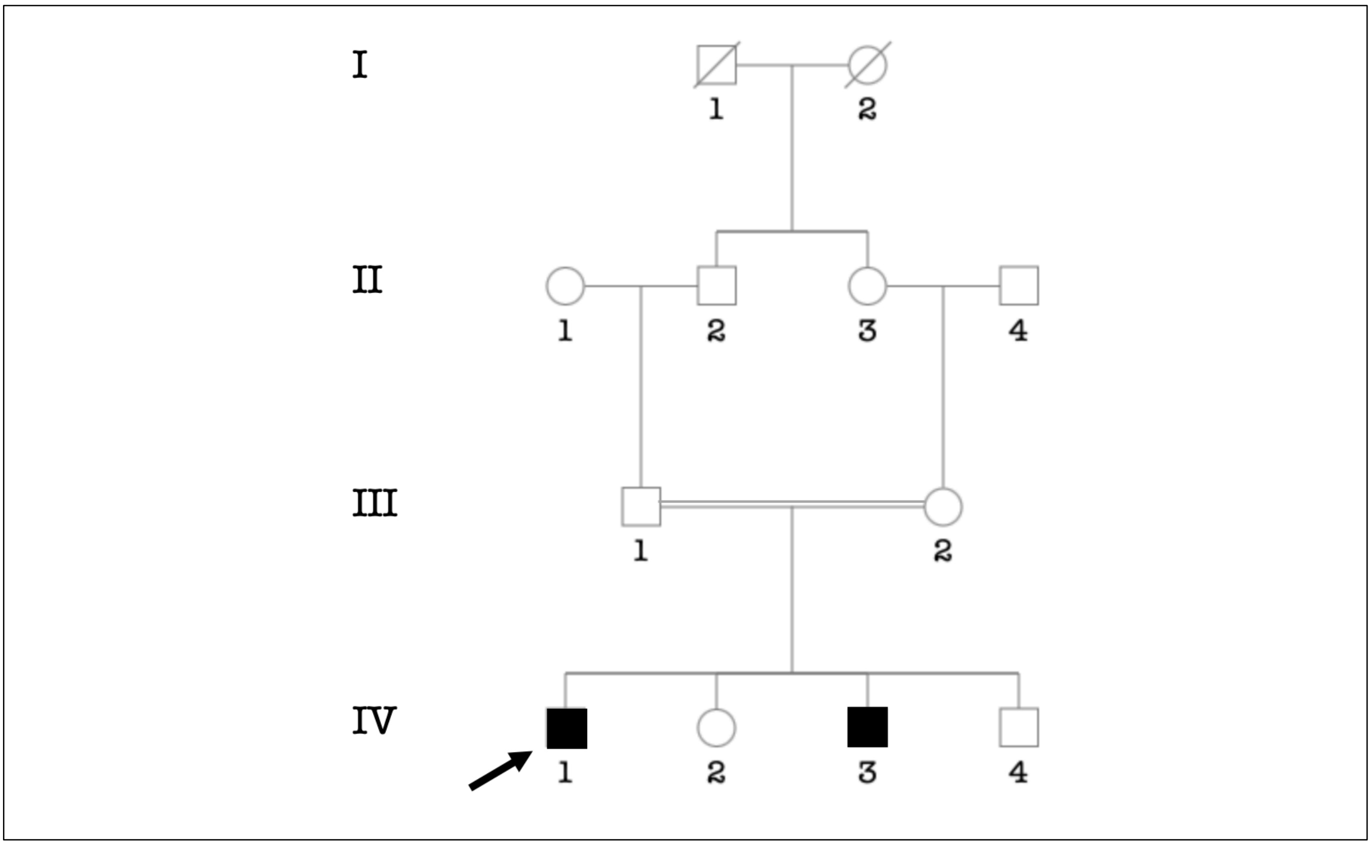

Family81. Pedigree showing two affected boys from a consanguineous marriage segregating autosomal recessive non-syndromic hearing impairment

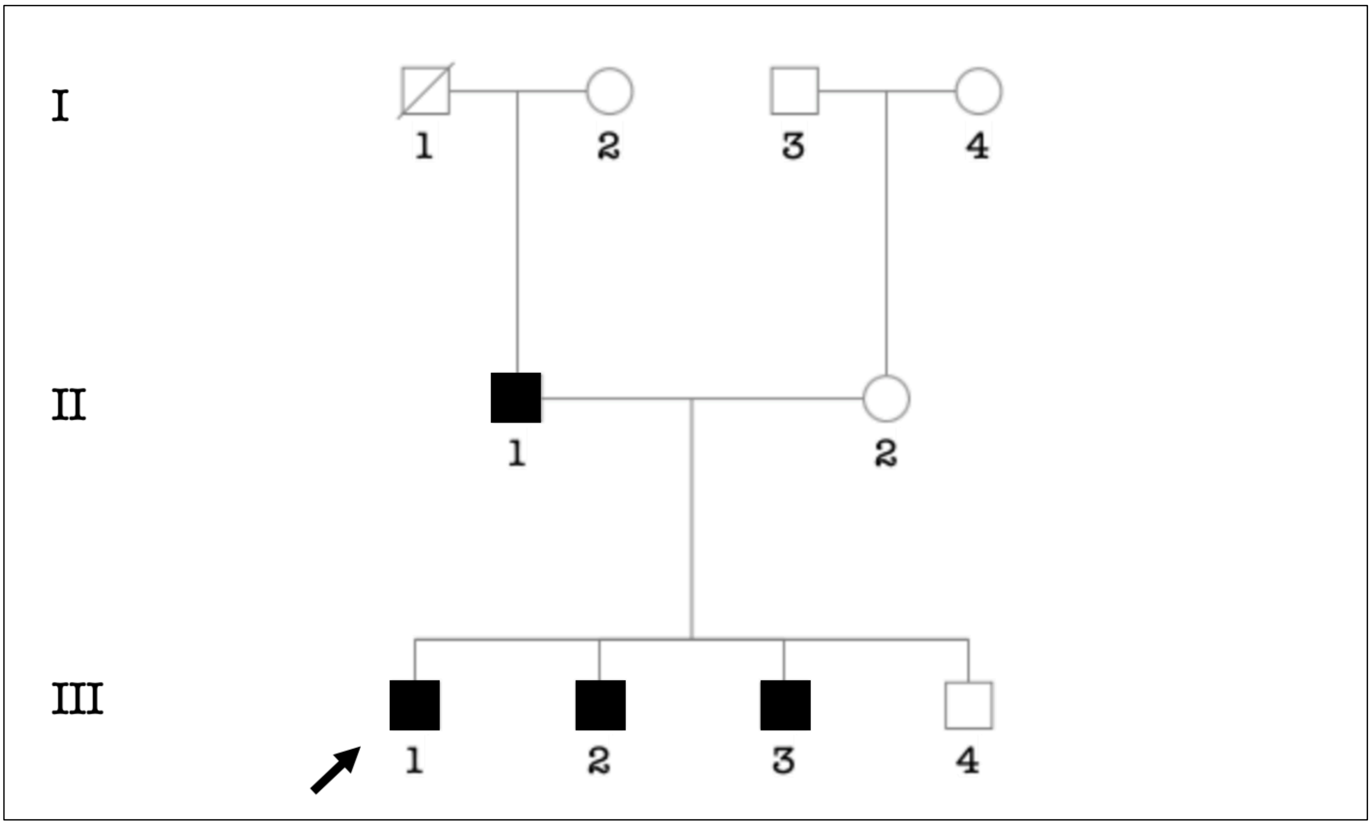

Family82. Pedigree showing four affected individuals segregating autosomal recessive non-syndromic hearing impairment

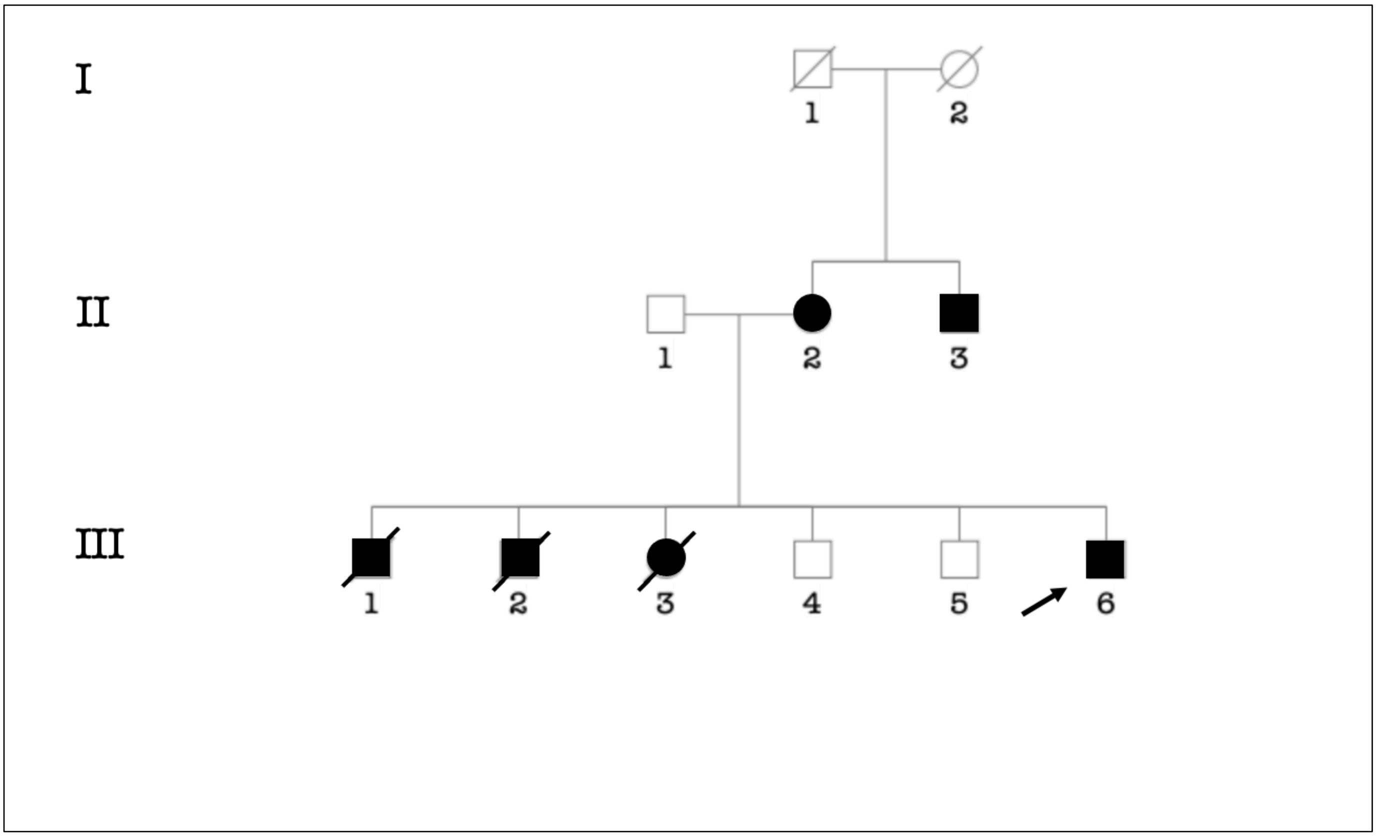

Family83. Pedigree showing six affected individuals segregating Alport syndrome, (Dominant X-linked).

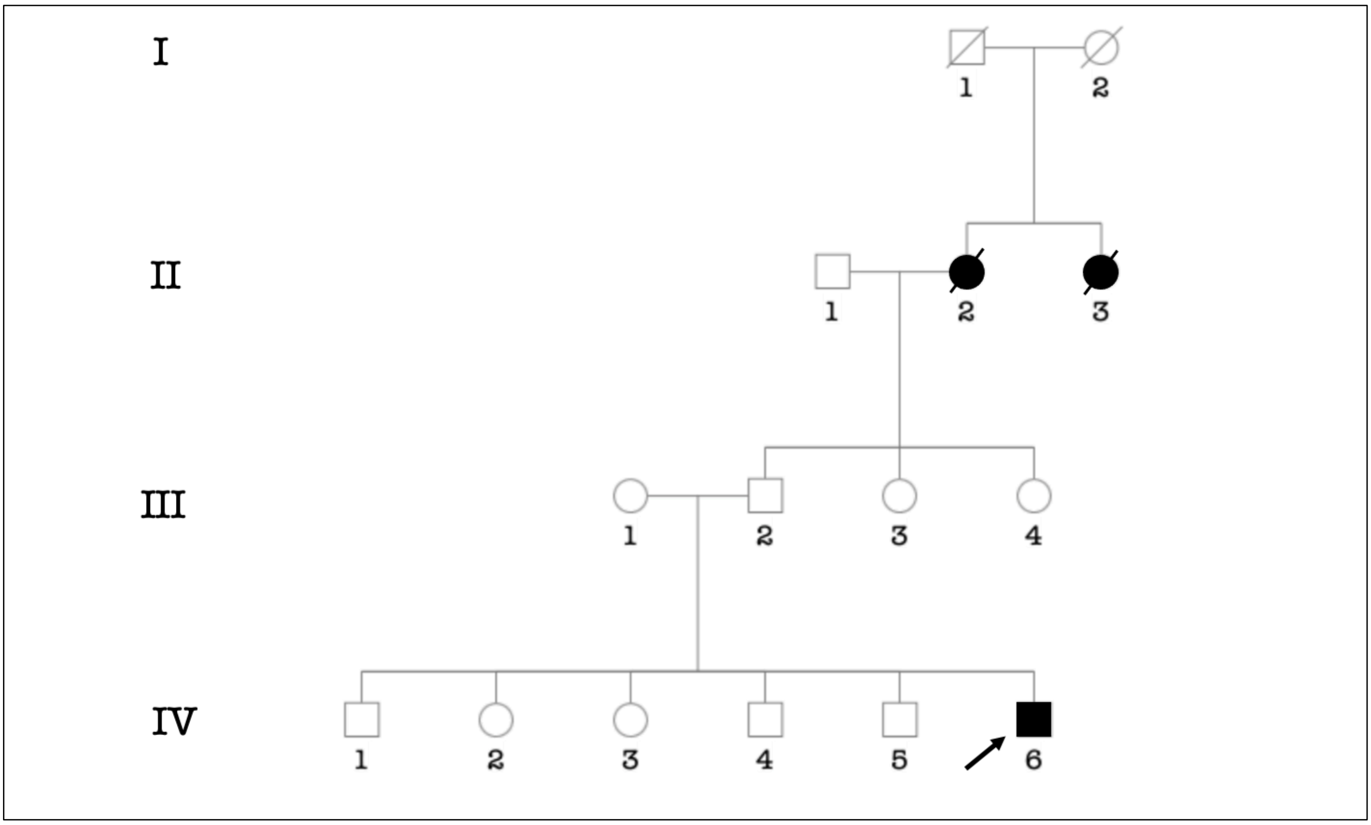

Family84. Pedigree showing a proband indicates by an arrow with non-syndromic hearing impairment

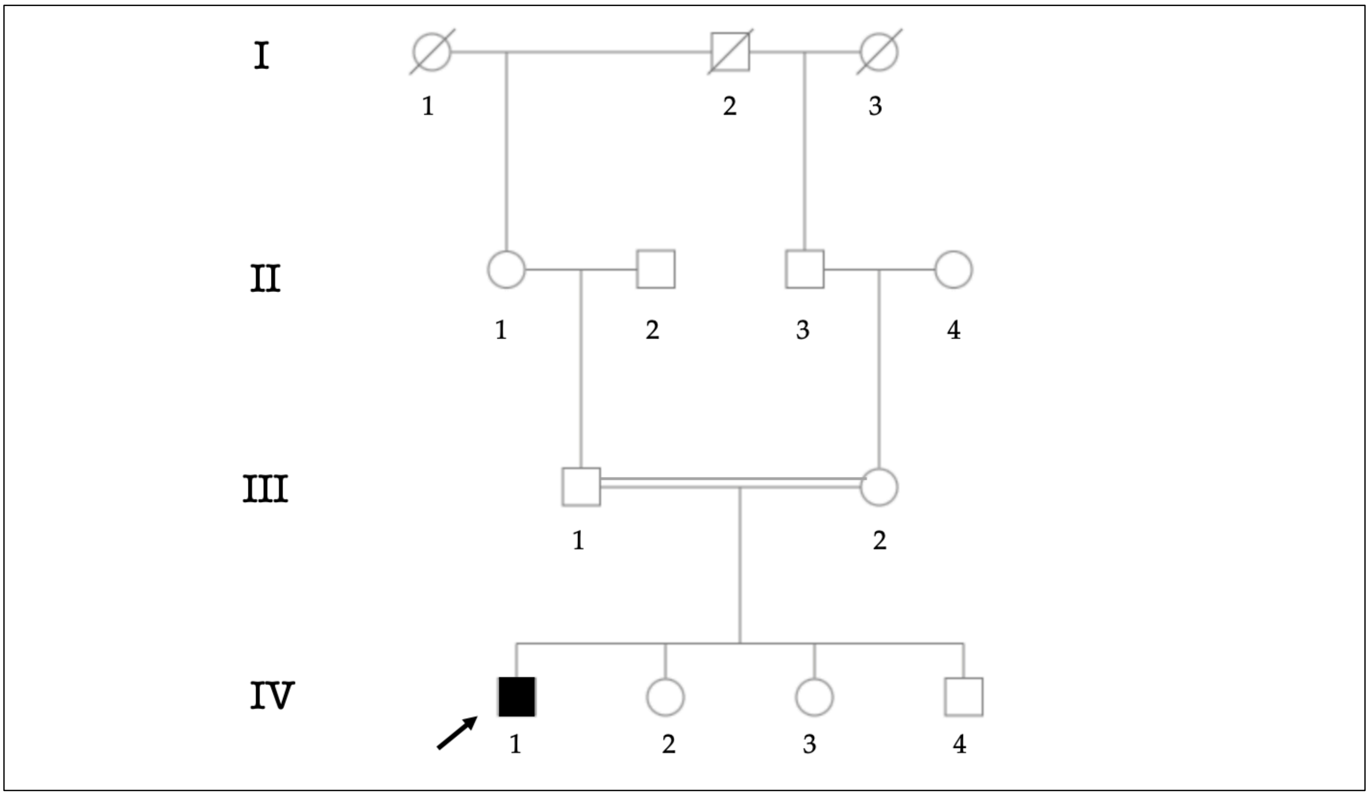

Family85. Pedigree showing a proband indicates by an arrow with non-syndromic hearing impairment

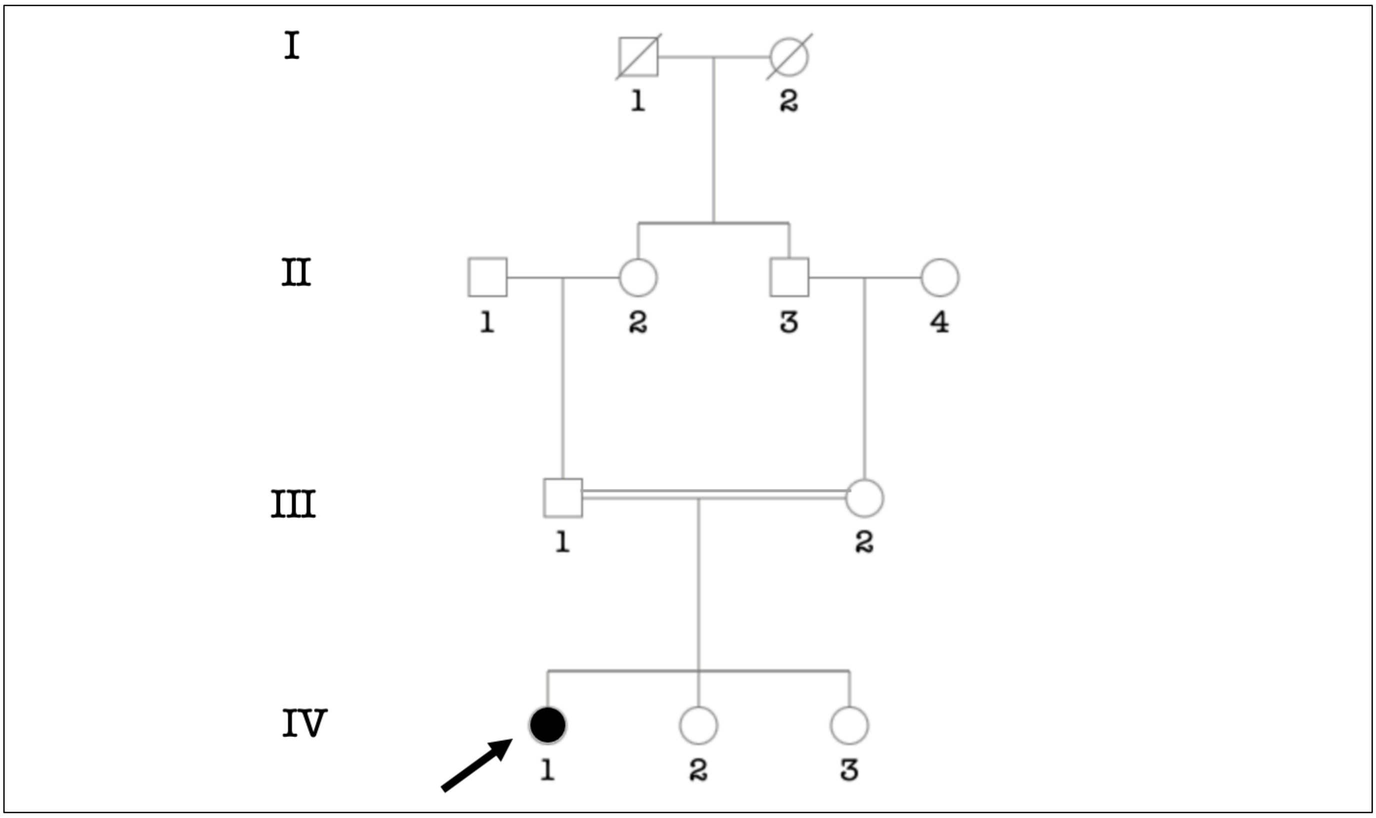

Family86. Pedigree showing a proband indicates by an arrow with non-syndromic hearing impairment

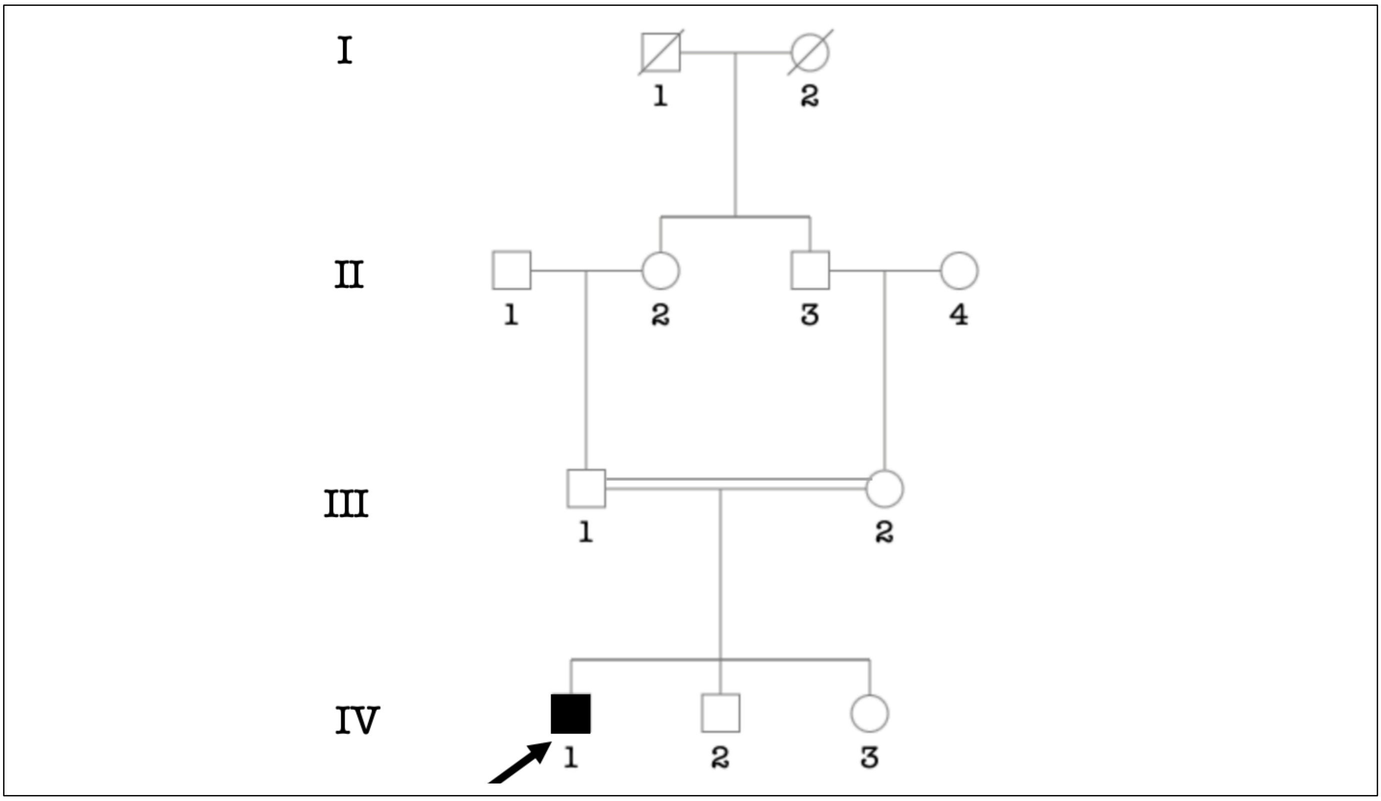

Family87. Pedigree showing a proband indicates by an arrow with non-syndromic hearing impairment

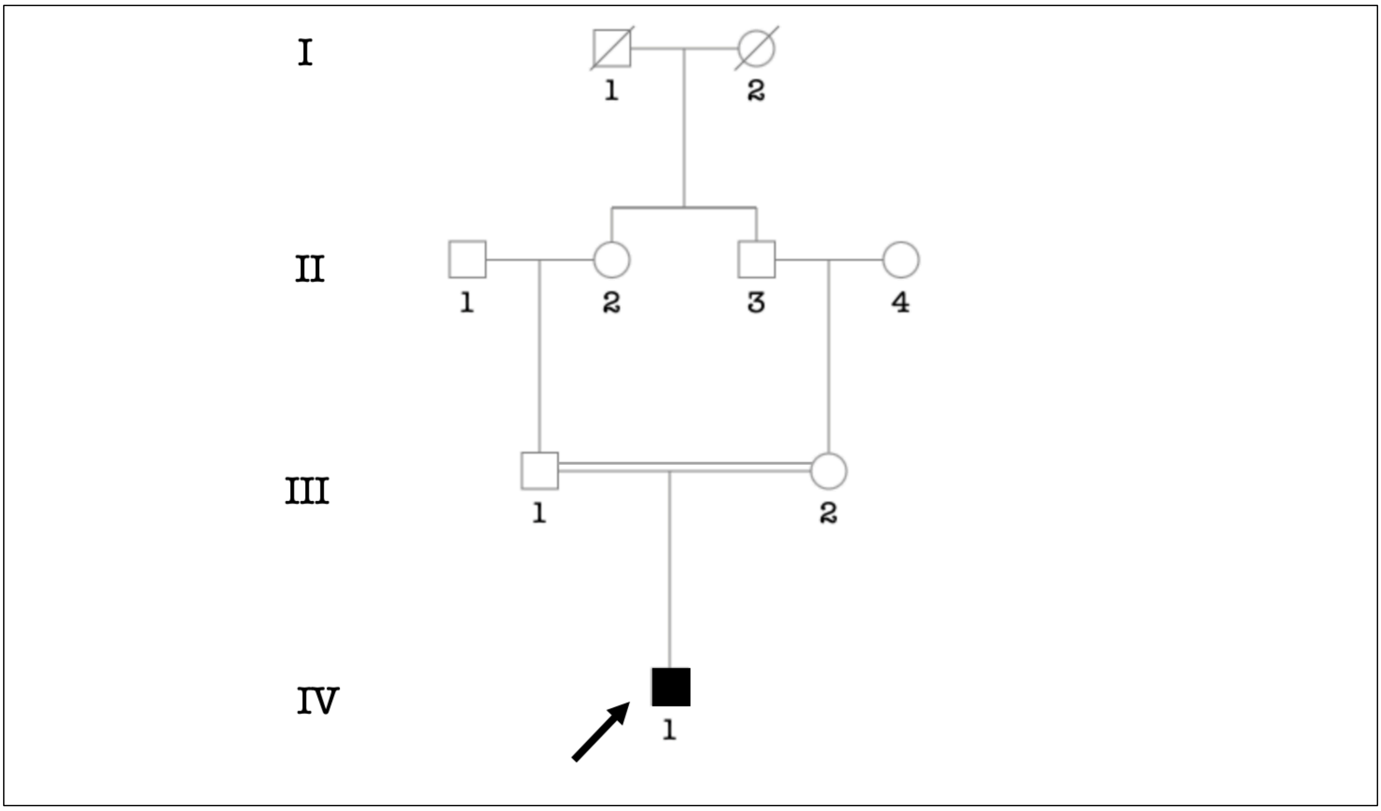

Family88. Pedigree showing a proband indicates by an arrow with non-syndromic hearing impairment

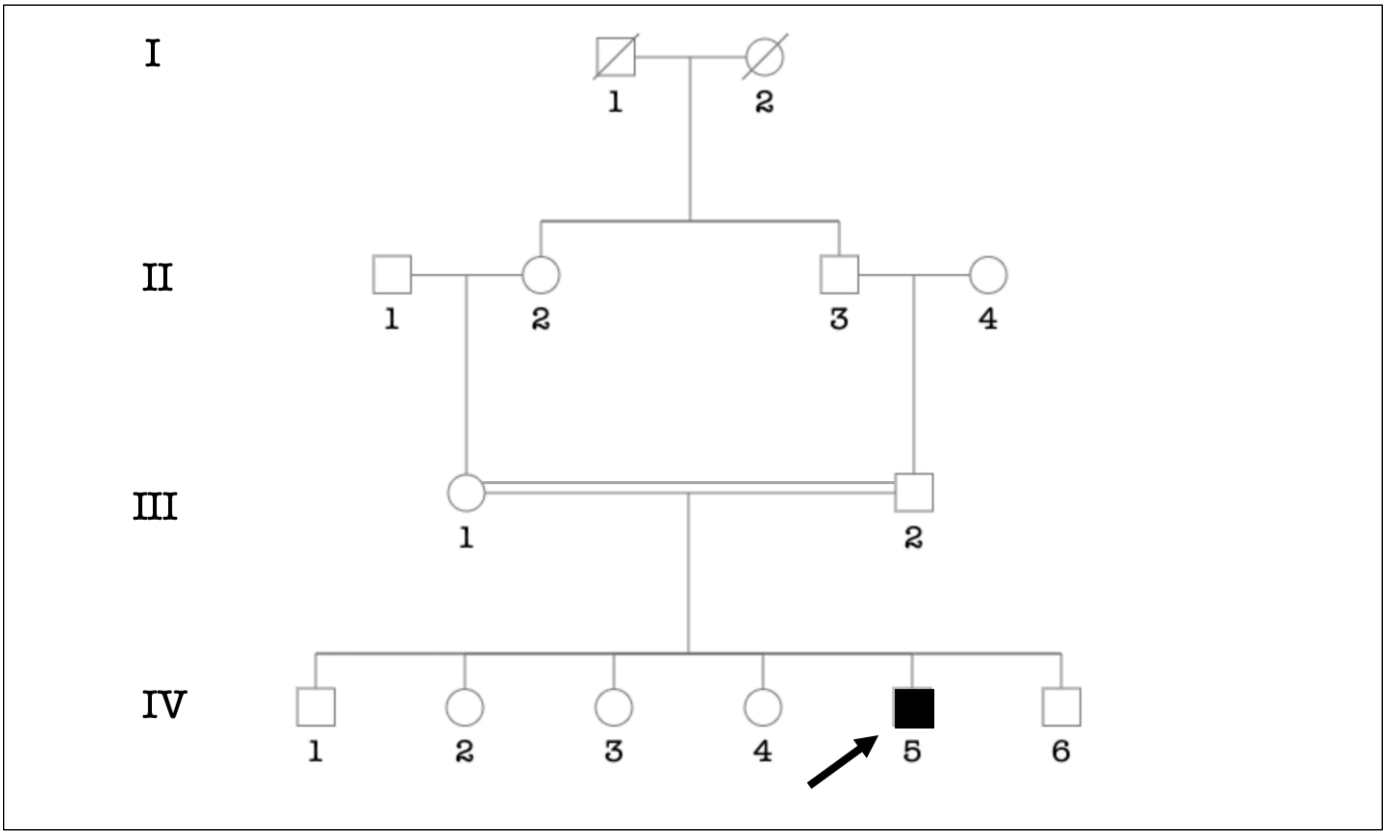

Family89. Pedigree showing a proband indicates by an arrow with non-syndromic hearing impairment

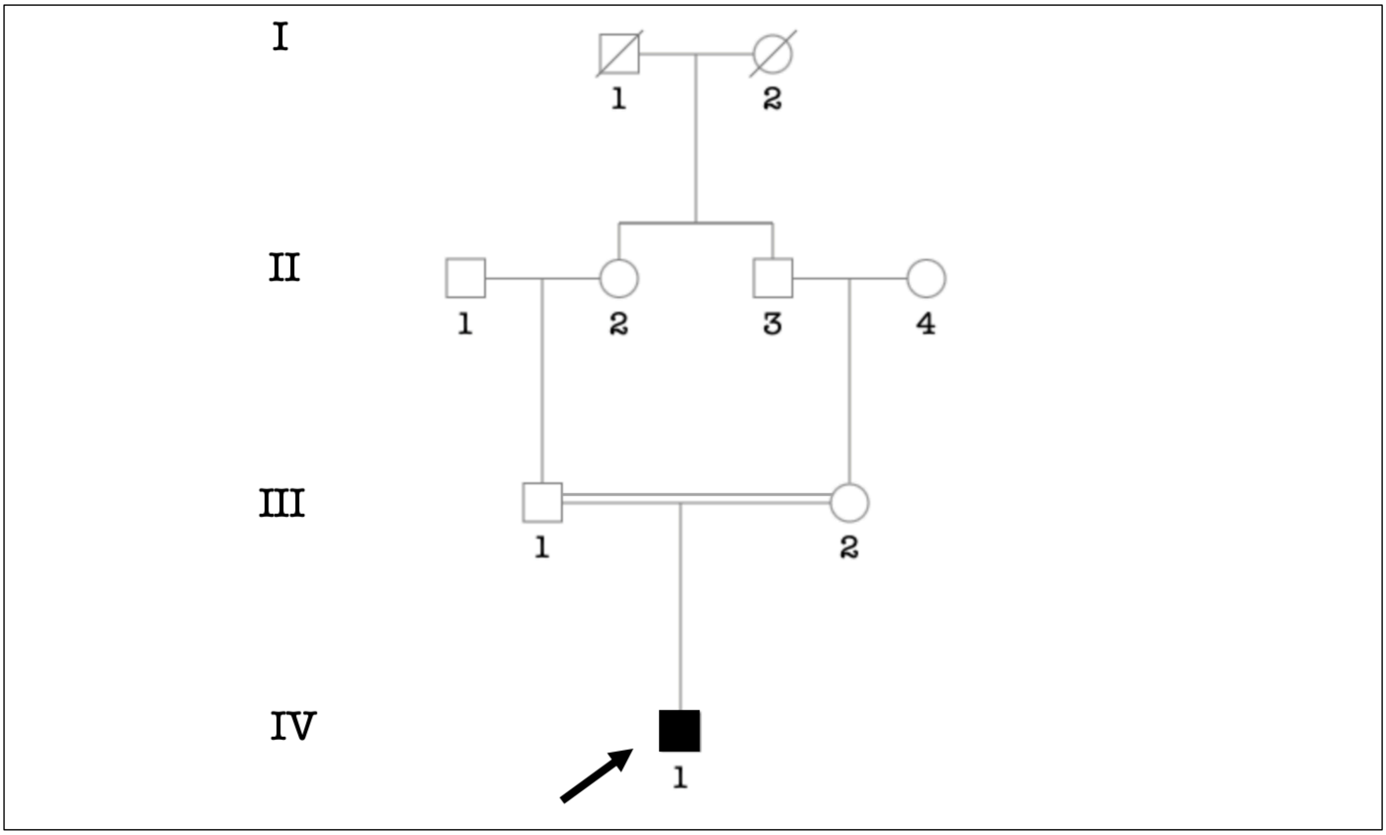

Family90. Pedigree showing a proband indicates by an arrow with non-syndromic hearing impairment

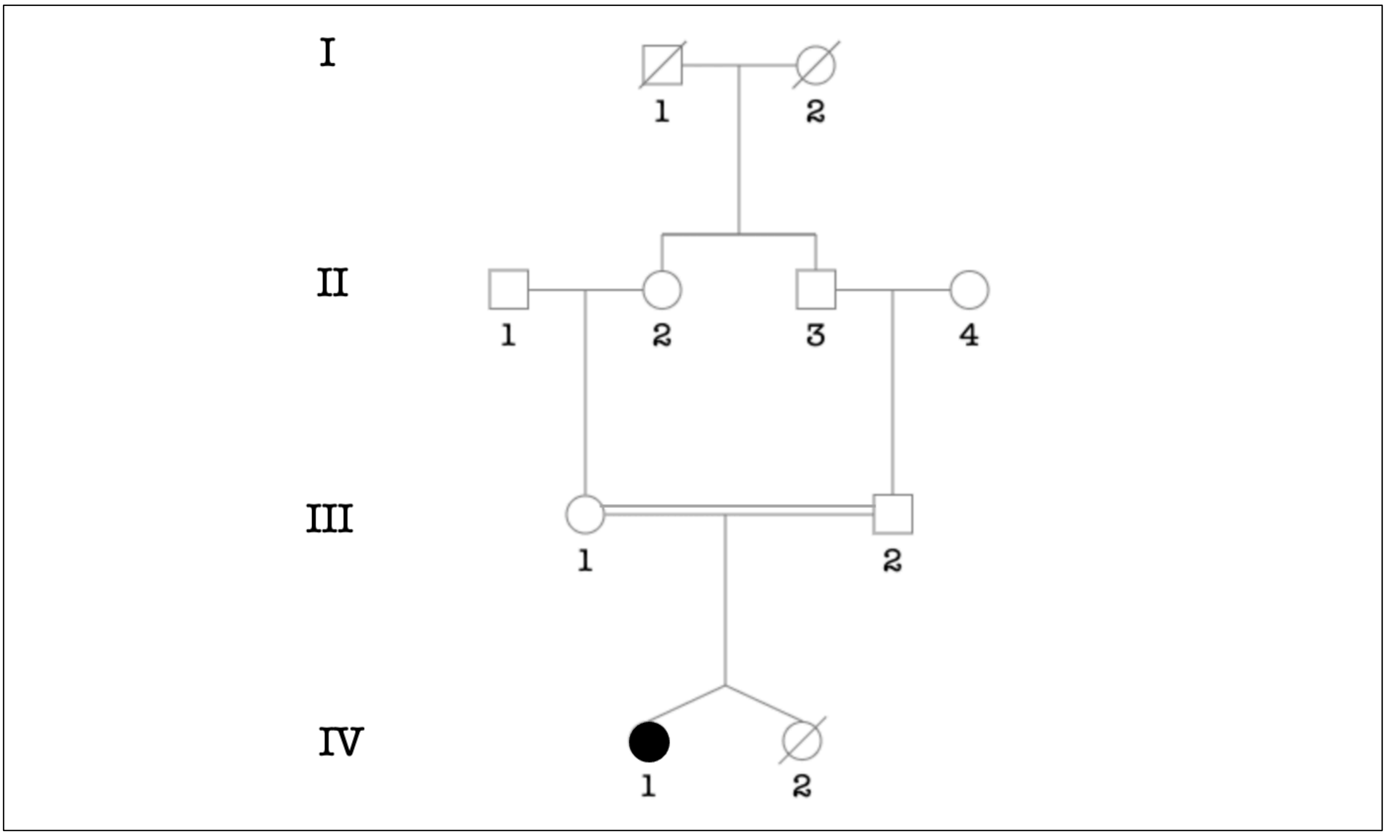

Family91. Pedigree showing a proband indicates by an arrow with non-syndromic hearing impairment

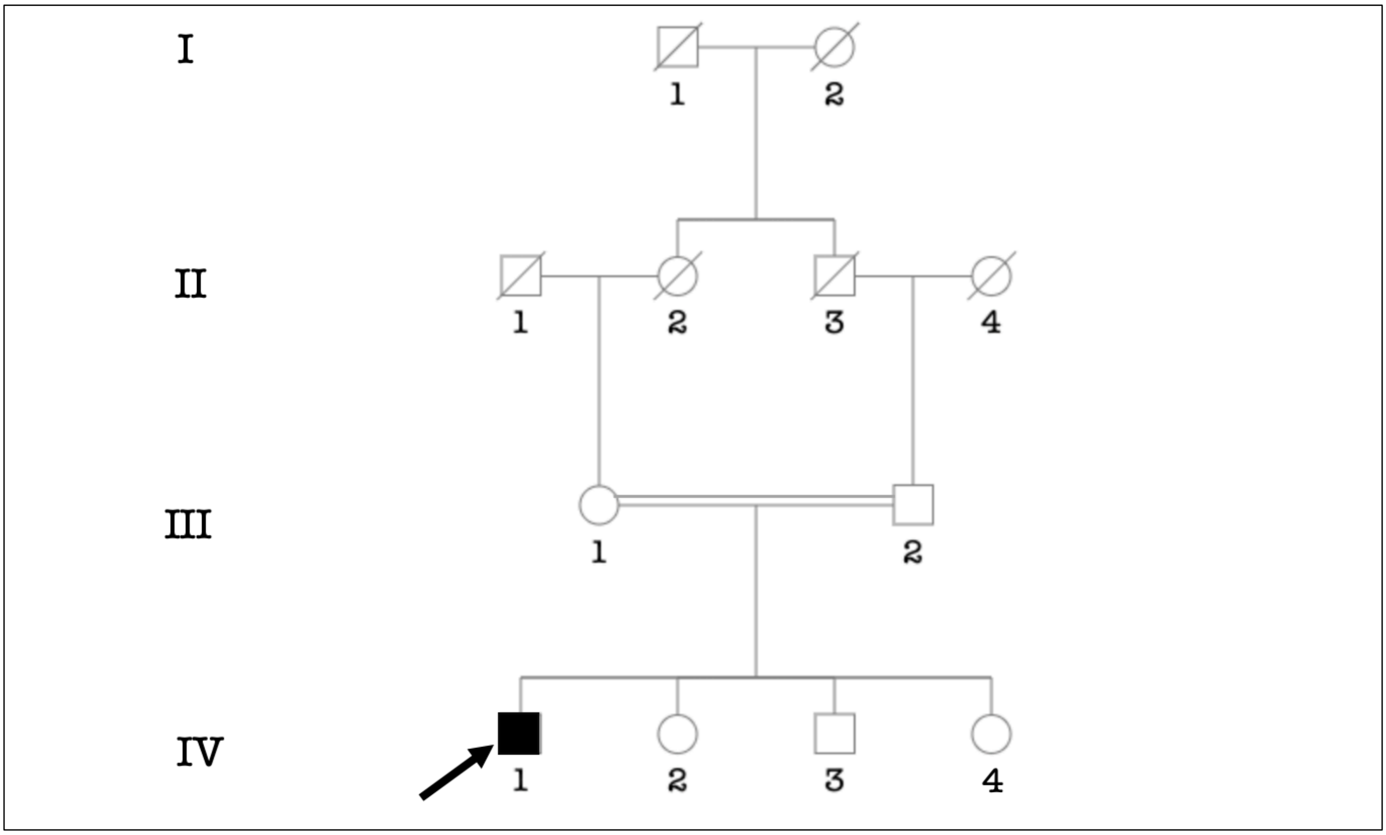

Family92. Pedigree showing a proband indicates by an arrow with non-syndromic hearing impairment

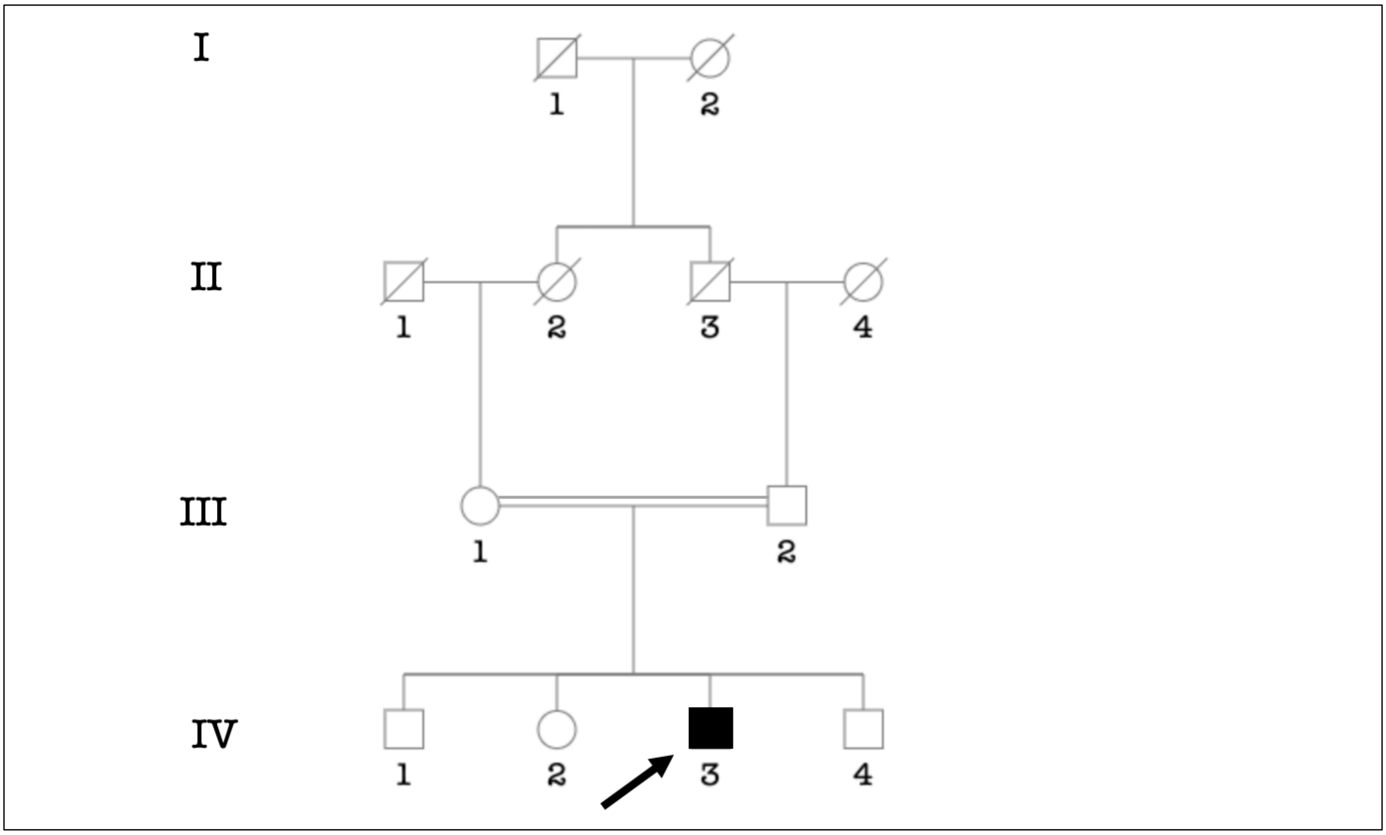

Family93. Pedigree showing a proband indicates by an arrow with non-syndromic hearing impairment

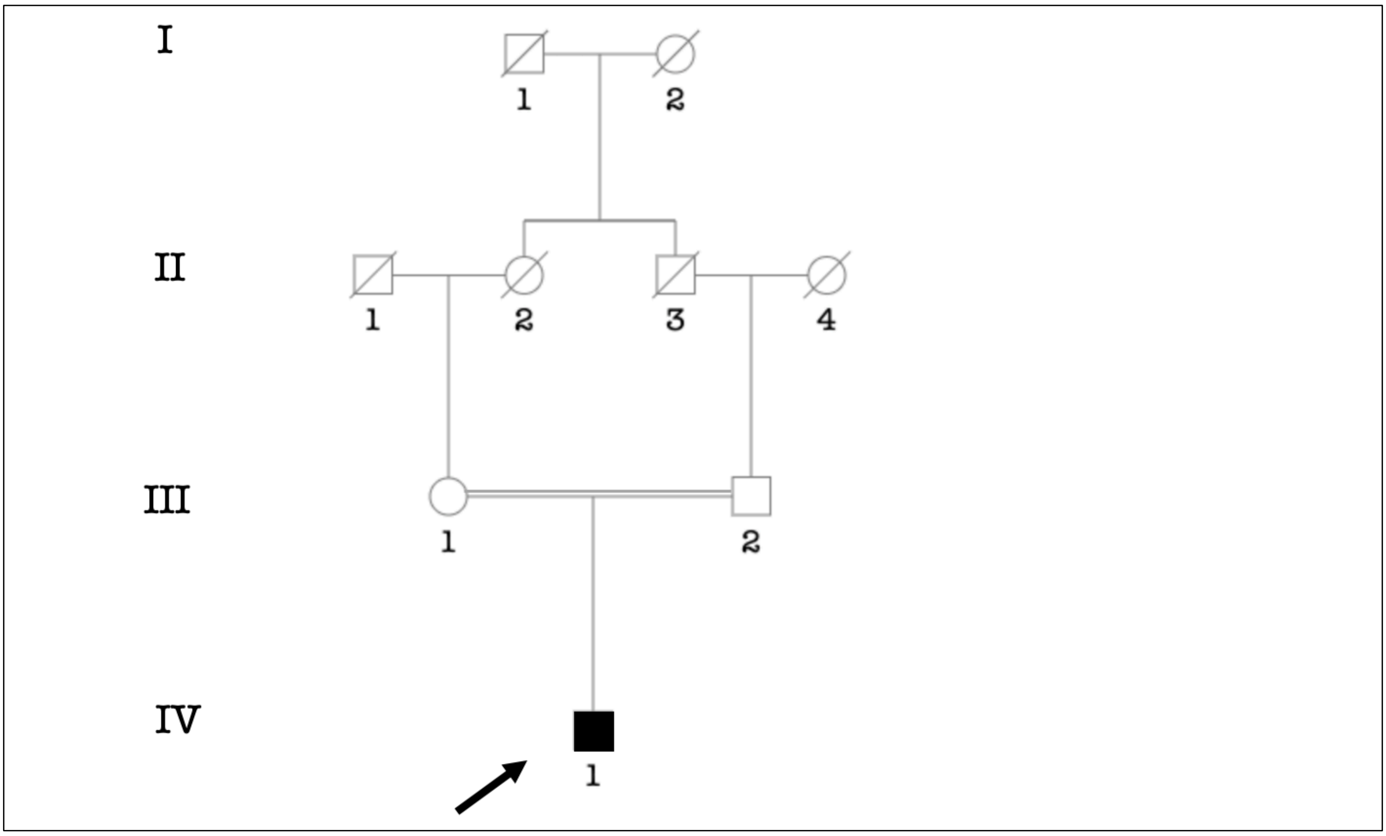

Family94. Pedigree showing a proband indicates by an arrow with non-syndromic hearing impairment

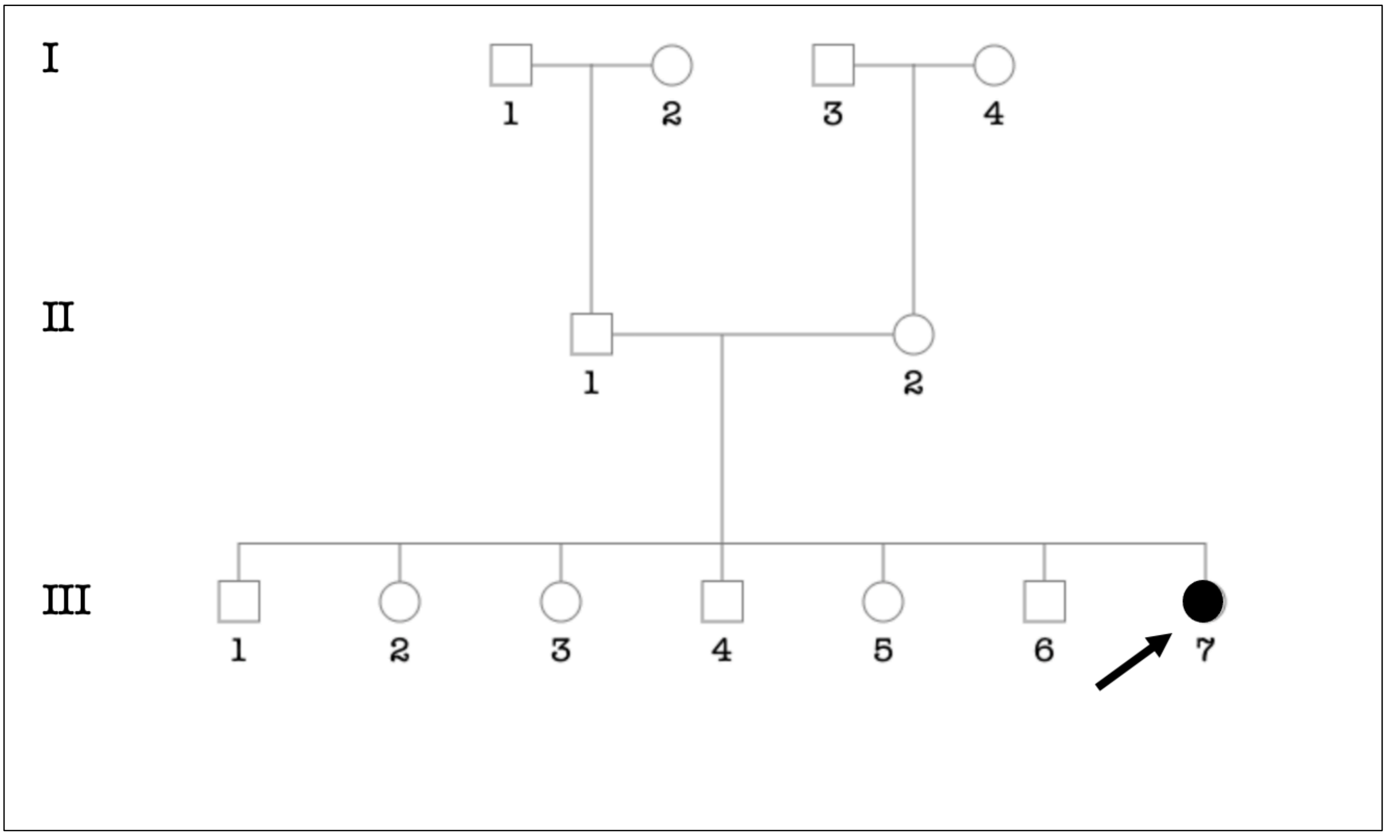

Family95. Pedigree showing a proband indicates by an arrow with Down's syndrome

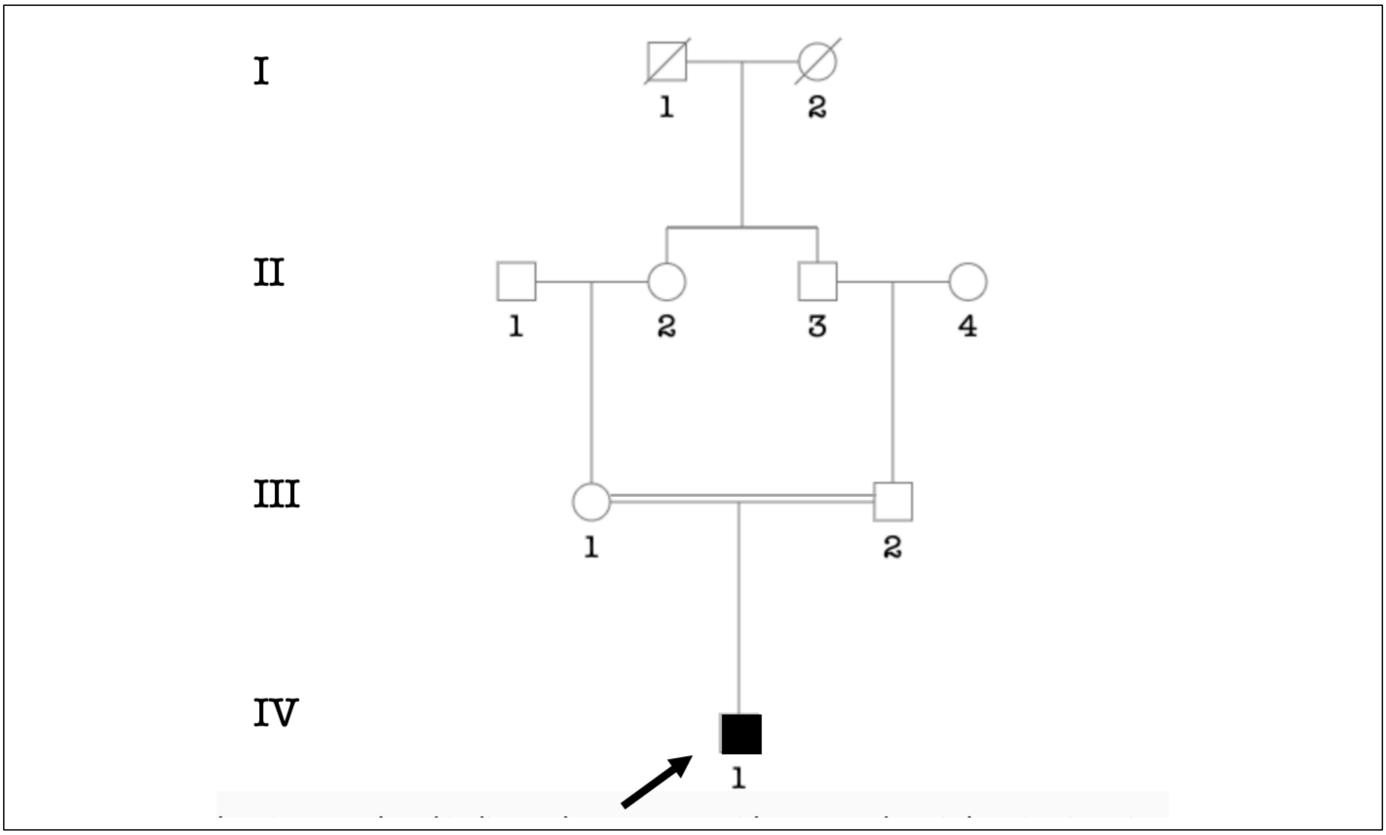

Family96. Pedigree showing a proband indicates by an arrow with non-syndromic hearing impairment

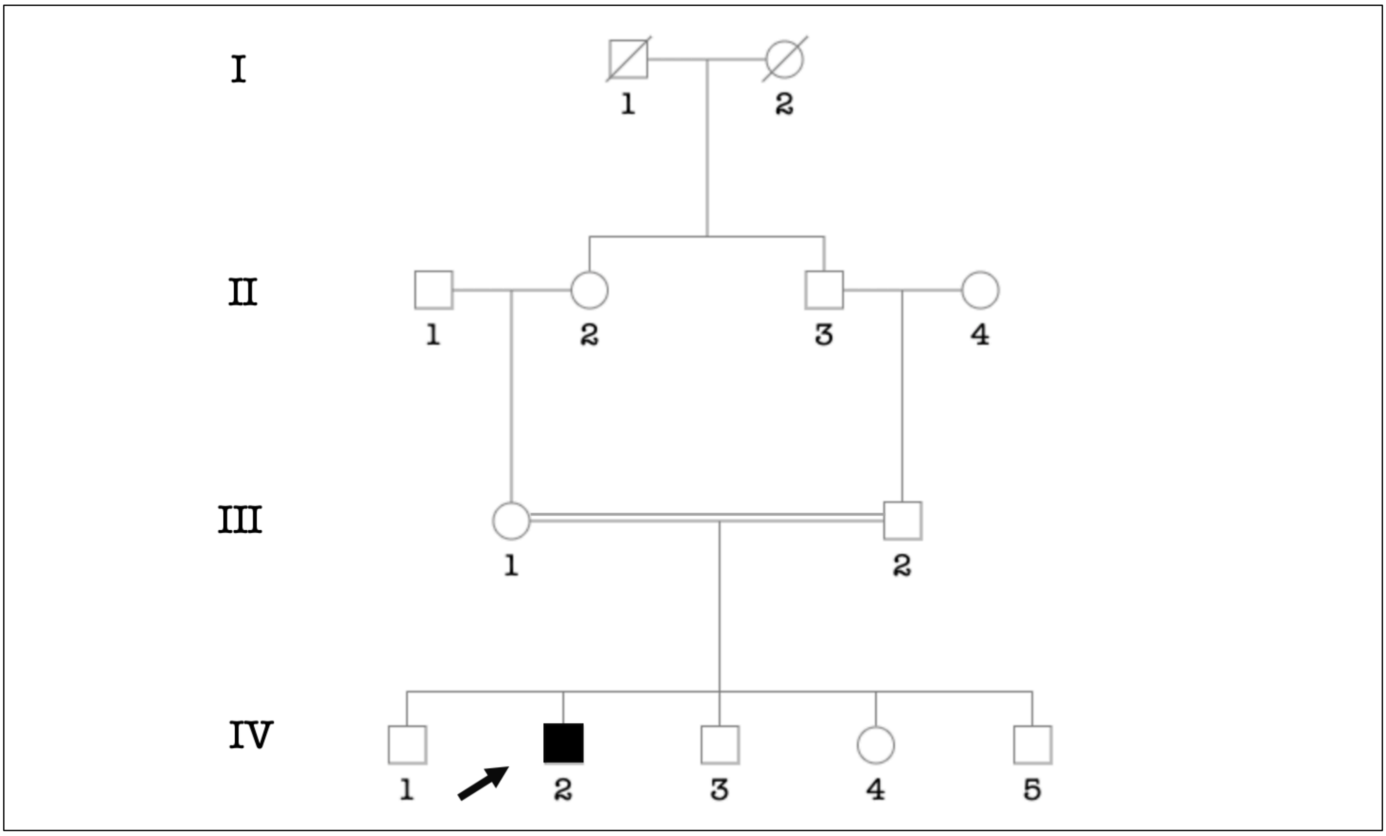

Family97. Pedigree showing a proband indicates by an arrow with non-syndromic hearing impairment

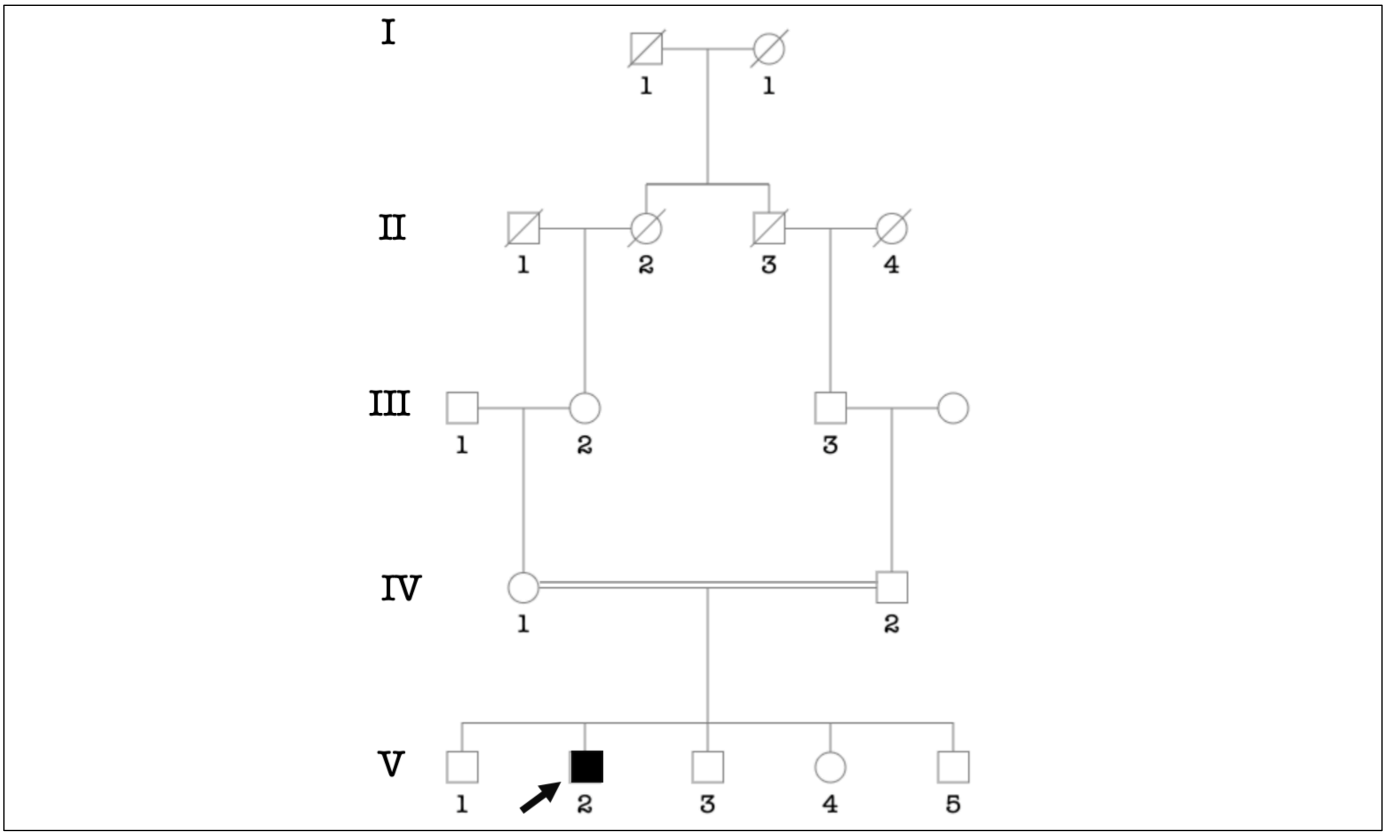

Family98. Pedigree showing a proband indicates by an arrow with non-syndromic hearing impairment

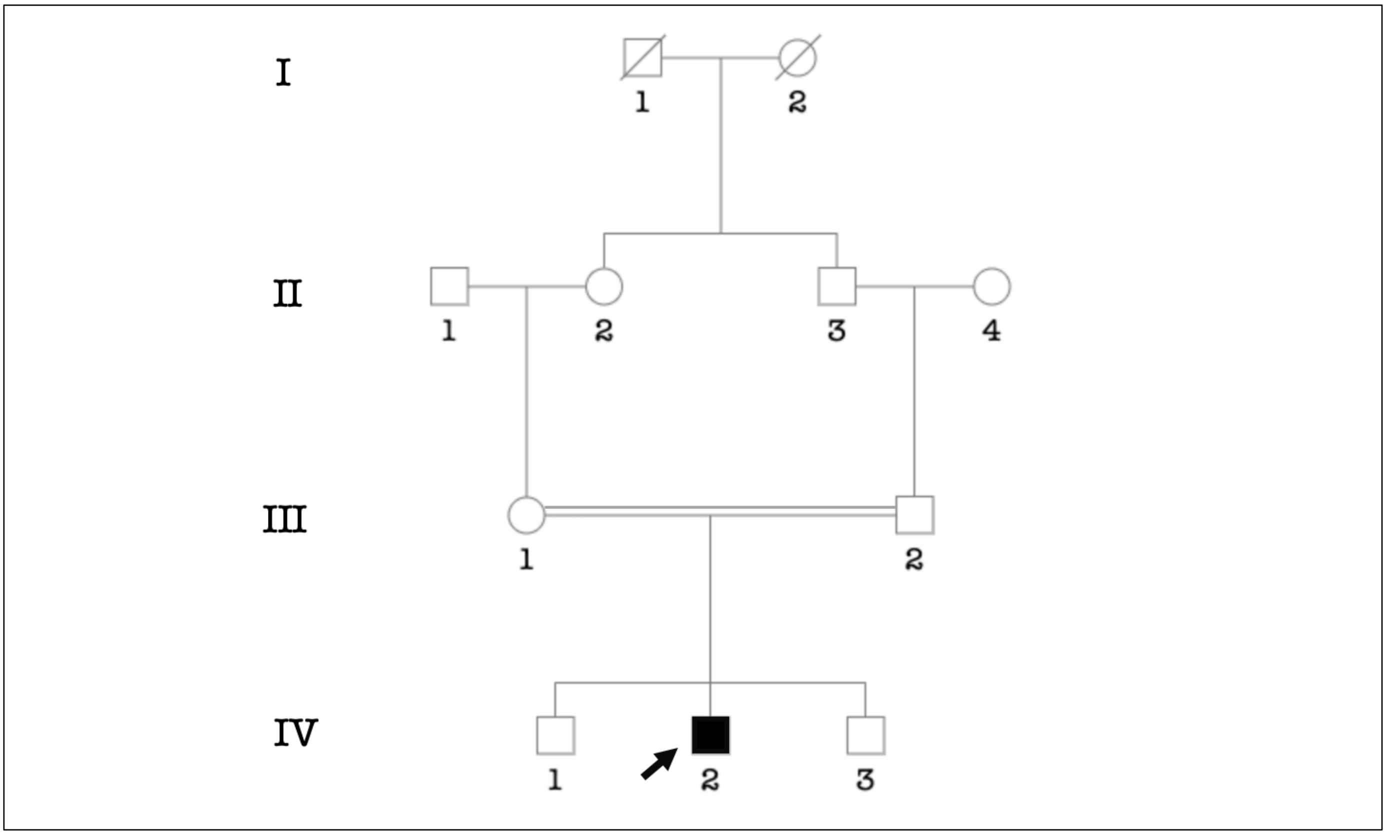

Family99. Pedigree showing a proband indicates by an arrow with non-syndromic hearing impairment

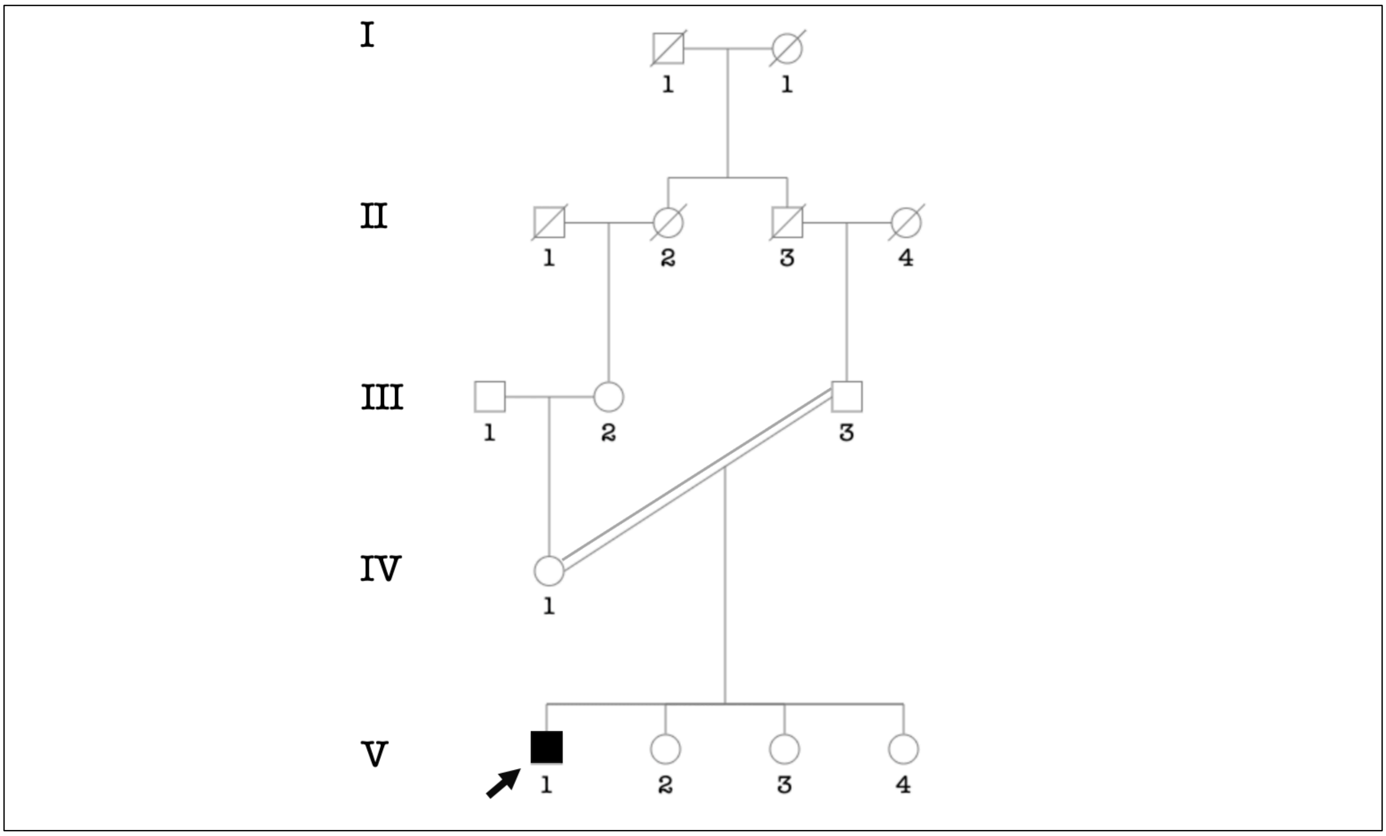

Family100. Pedigree showing a proband indicates by an arrow from a consanguineous marriage with non-syndromic hearing impairment

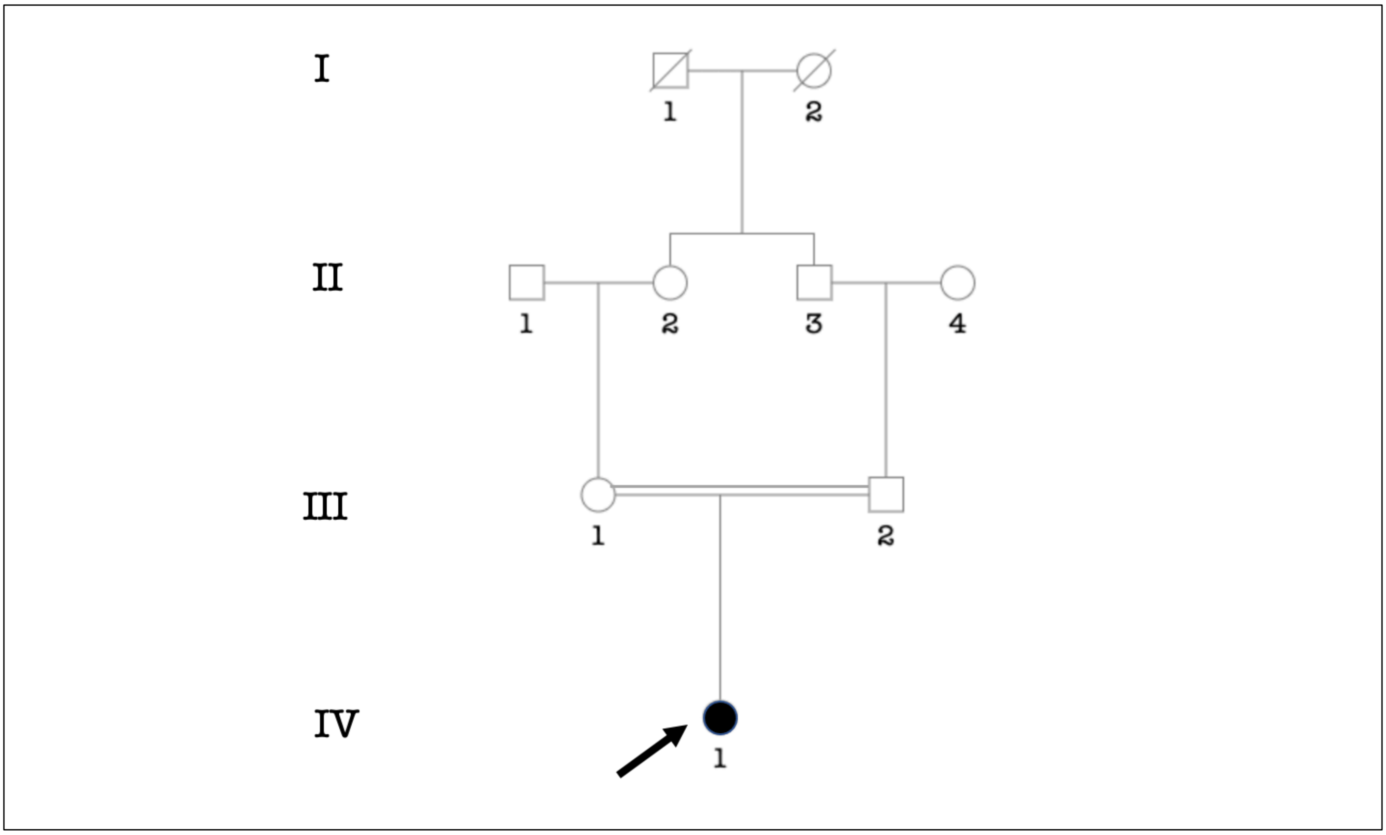

Family101. Pedigree showing a proband indicates by an arrow from a consanguineous marriage with non-syndromic hearing impairment

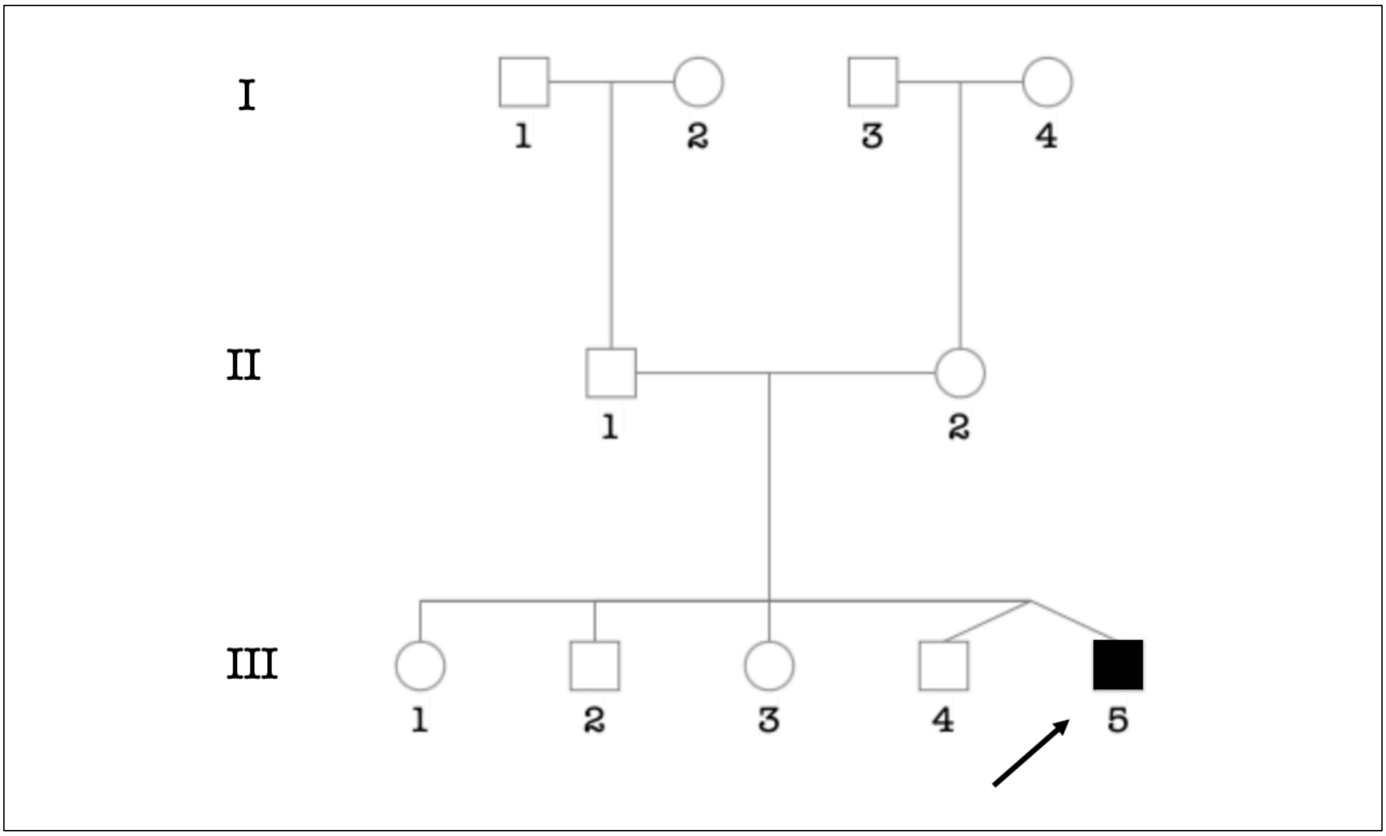

Family102. Pedigree showing a proband indicates by an arrow with type 2 Waardenburg syndrome

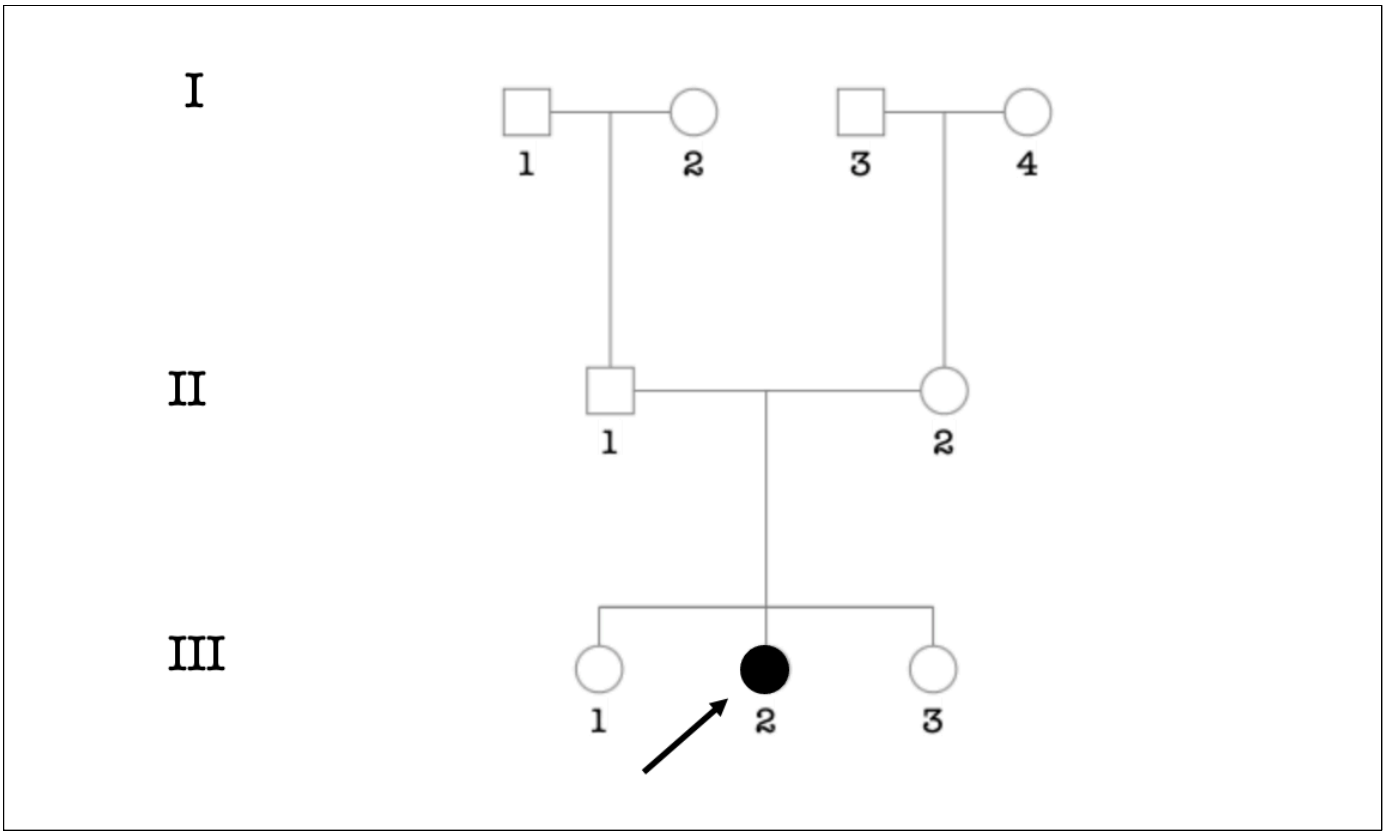

Family103. Pedigree showing a proband indicates by an arrow with suspected LAMM syndrome

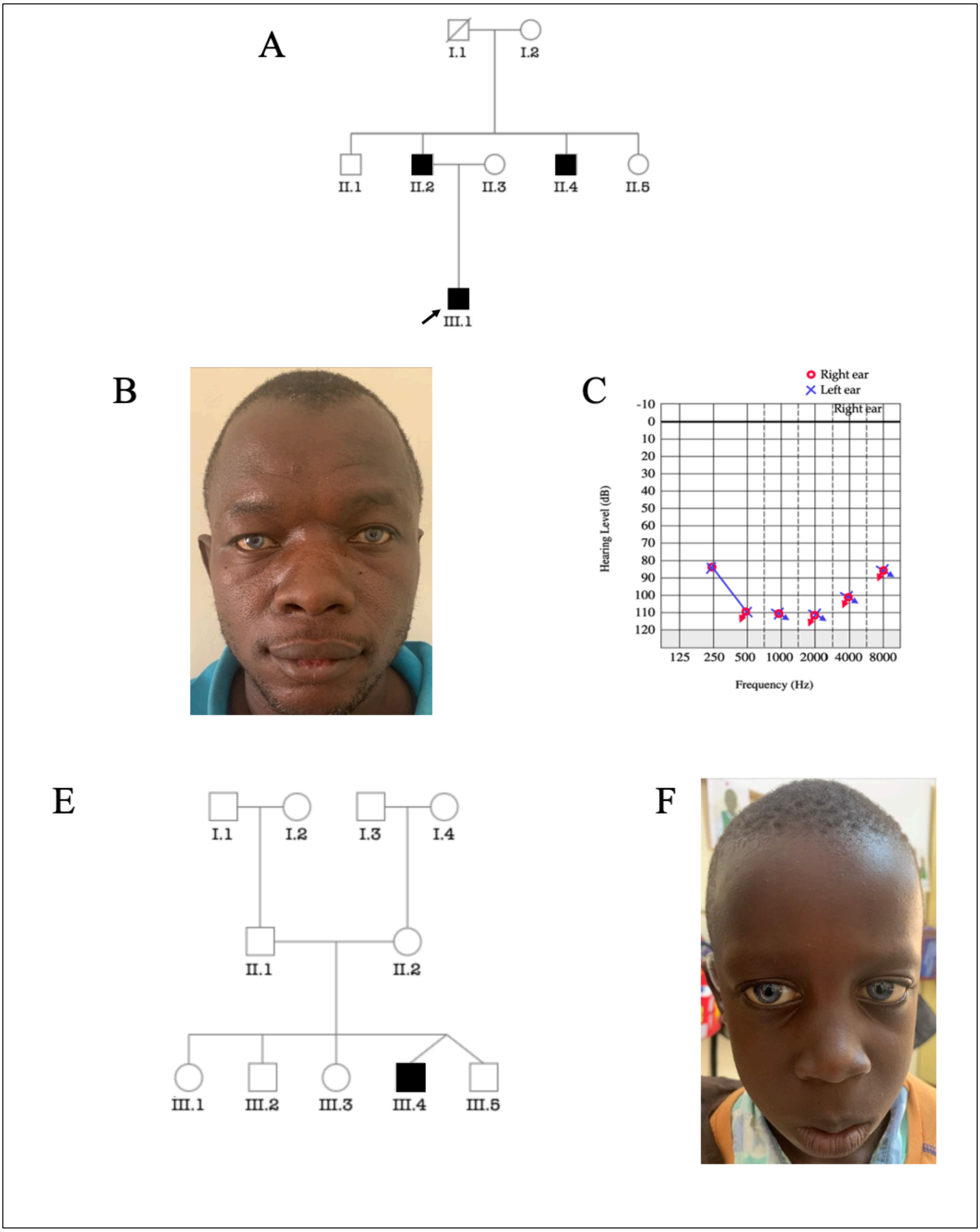

Figure S2. Waardenburg Syndrome type 2 in two Senegalese Families. In the first family (Panel A) the proband (III.1) as well as his father (II.2) exhibited blue sapphire eye. In addition to profound hearing impairment the proband' father showed premature white hair. In the second family (Panel E) the proband (III.4) exhibited blue sapphire eye associated with profound hearing impairment.
